# The global macroeconomic burden of diabetes mellitus

**DOI:** 10.1038/s41591-025-04027-5

**Published:** 2025-12-29

**Authors:** Simiao Chen, Zhong Cao, Wenjin Chen, Jinghan Zhao, Lirui Jiao, Klaus Prettner, Michael Kuhn, An Pan, Till Winfried Bärnighausen, David E. Bloom

**Affiliations:** 1 Heidelberg Institute of Global Health, Faculty of Medicine and University Hospital, Heidelberg University, Heidelberg, Germany; 2 School of Population Medicine and Public Health, Chinese Academy of Medical Sciences and Peking Union Medical College, Beijing, China; 3 University of North Carolina at Chapel Hill, Chapel Hill, NC USA; 4 Department of Economics, Vienna University of Economics and Business (WU), Vienna, Austria; 5 International Institute for Applied Systems Analysis (IIASA), Laxenburg, Austria; 6 Huazhong University of Science and Technology, Wuhan, China; 7 Harvard T.H. Chan School of Public Health, Harvard University, Boston, MA USA

**Keywords:** Type 2 diabetes, Economics

## Abstract

Diabetes mellitus poses a substantial and rising global health and economic burden, affecting more than one in ten adults worldwide. Using a health-augmented macroeconomic model across 204 countries and territories, we estimated the economic impact of diabetes from 2020 to 2050, incorporating losses in effective labor supply due to mortality and morbidity, treatment-related resource diversion and informal caregiving costs. Without informal care, the global burden amounts to $10.2 trillion (2017 international dollars (INT$)), equivalent to 0.22% of annual global gross domestic product. Including informal care, the burden increases dramatically to INT$78.8 trillion, ranging from INT$5.5 trillion to INT$152.1 trillion, depending on the assumptions for care. The absolute costs are highest in the United States, China and India, while relative and per capita burdens are greatest in countries such as American Samoa and Australia. These findings highlight the uneven distribution of diabetes’ economic impact and underscore the urgent need for effective global interventions.

## Abstract (web summary)

An analysis of 204 countries estimates that diabetes will cost the global economy $10.2 trillion between the years 2020 and 2050.

## Main

Diabetes mellitus has been one of the top ten drivers of the growing global health burden over the past 30 years^1,2^, propelled by aging populations and increasing environmental and behavioral risks such as air pollution and obesity. In 2021, more than one in ten adults worldwide (537 million people) had diabetes mellitus, and more than three-quarters of them lived in low- and middle-income countries (LMICs). Almost half (45%) of adults aged 20–79 years with diabetes mellitus were unaware of their condition, and about 90% of these individuals lived in LMICs. By 2045, 783 million adults worldwide are expected to have diabetes mellitus, highlighting the growing challenges^3^. The health burden of diabetes mellitus is unevenly distributed among countries. China is home to the greatest number of individuals aged 20–79 years with diabetes mellitus, followed by India, Pakistan and the United States^3^. Supplementary Figs. 1–3 show the detailed information on the incidence, the mortality rate and the prevalence of diabetes mellitus in 2021, while Supplementary Fig. 4 shows the trend in diabetes mellitus-related death numbers from 1980 to 2050.

The COVID-19 pandemic has increased the burden of diabetes mellitus beyond previous levels. Existing research indicates that diabetes mellitus poses a risk factor for severe complications, hospitalization and death among COVID-19 patients^4–7^. Furthermore, COVID-19 also increases the risk of developing type 2 diabetes^8,9^. For instance, a cohort study revealed that in the postacute phase of illness, individuals with COVID-19 showed an increased risk of incident diabetes (hazard ratio (HR) = 1.40, 95% confidence interval (CI) = 1.36–1.44) in comparison to the control group^8^.

In addition to imposing substantial pain on patients and their relatives and an enormous population-wide health burden, diabetes mellitus also imposes a substantial economic burden. According to the International Diabetes Federation (IDF), diabetes contributed to at least 966 billion (in 2021 US dollars) in global health expenditures (direct costs) in 2021, representing 11.5% of all global health expenditures that year and marking a 316% rise in spending relative to 15 years prior. The IDF has also projected that global diabetes-related health expenditures will rise to 1.05 trillion (in 2021 US dollars) by 2045 (ref. ^3^). The economic burden of diabetes mellitus is unevenly distributed, with high-income countries facing the highest level of diabetes-related health expenditure as a percentage of gross domestic product (GDP; 1.16%) in 2021, followed by middle-income countries (1.08%) and low-income countries (0.51%)^3^. Among individual countries, the highest total health expenditure on diabetes in 2021 was incurred by the United States (379.5 billion in 2021 US dollars), followed by China (165.3 billion in 2021 US dollars) and Brazil (42.9 billion in 2021 US dollars), and the highest diabetes-related expenditure per patient was incurred by Switzerland (12,828 in 2021 US dollars), followed by the United States (11,779 in 2021 US dollars) and Norway (11,166 in 2021 US dollars)^3^. However, data limitations, such as lack of reliable prevalence and mortality estimates, have prevented accurate assessment of the economic burden of diabetes mellitus in LMICs^10^. Reasonable projections of the economic burden of diabetes mellitus and its distribution across countries are urgently needed to inform the design of evidence-based policies for curbing the disease’s impact.

Although several studies on the global or regional economic burden of diabetes mellitus exist, most are based on a summation of the direct and indirect costs of the disease (cost-of-illness approach)^10–13^. One study using this approach estimated that the absolute global economic burden of diabetes mellitus will reach 2.2 trillion in constant 2015 US dollars by 2030, accounting for up to 2.2% of annual global GDP^12^. Another analysis projected that, by 2030, the total economic burden of diabetes in China will reach 460.4 billion (in nominal US dollars), representing up to 1.69% of the nation’s GDP^13^. However, cost-of-illness studies often overlook economic adjustment mechanisms—for example, that jobs do not remain vacant indefinitely because new workers or physical capital (such as machines or robots) replace the lost labor—that could considerably impact their results. In addition, such approaches are static and disregard the effect of diabetes mellitus on human and physical capital accumulation. To address these shortcomings, the World Health Organization established the Economic Projections for Illness and Cost of treatment (EPIC) model for assessing the economic burden of diseases in 2006. EPIC advanced beyond the cost-of-illness method by incorporating economic adjustments and the effects of disease on human and physical capital accumulation. However, it did not account for the dependency of productivity losses on the distribution of education and experience levels, nor the economic effects of morbidity and treatment costs^14^. Finally, both EPIC and other previous approaches have not fully accounted for the macroeconomic loss associated with informal caregiving for diabetes mellitus, despite the evidence that this burden is substantial.

To fill these gaps, we used a theory-grounded, health-augmented macroeconomic model to estimate the macroeconomic burden of diabetes mellitus in 204 countries from 2020 to 2050 and to find the distribution of that burden across world regions. This approach has previously been used to assess the economic burdens of noncommunicable diseases, road injuries, COVID-19 and risk factors like air pollution and tobacco use^15–20^. To avoid underestimating the cost of care for diabetes mellitus, we also considered the effects of informal care, including changes in labor force participation among family members who must care for diabetic patients. To our knowledge, no previous study has produced a comprehensive global estimate of the economic burden of diabetes mellitus by simulating an economy’s productive capacities at the aggregate level and the impact of diabetes mellitus on these capacities.

## Results

### Global macroeconomic burden of diabetes mellitus

We calculated the macroeconomic burden of diabetes mellitus as the difference in total GDP between 2020 and 2050 in the status quo scenario and a counterfactual scenario in which diabetes mellitus was eliminated. When not considering informal care loss, Table 1 shows the results for the 144 countries with complete data, representing 92.7% global population, and the imputed results for the 60 countries with partial data. The lower and upper boundaries computed in the sensitivity analysis using alternative mortality and morbidity data are included in brackets. To make country estimates comparable, all costs were converted to 2017 international dollars (INT$). The United States faces the largest economic burden of diabetes mellitus at INT$2.5 trillion, followed by India at INT$1.6 trillion and China at INT$1.0 trillion. When considering informal care loss, the largest economic burdens are INT$16.5 trillion in the United States, INT$11.4 trillion in India and INT$11.0 trillion in China (Supplementary Table 8). These results demonstrate that the economic burden of informal care for diabetes mellitus is particularly high. The economic burden of diabetes mellitus as a share of GDP ranges from 0.04% in Nigeria to 0.7% in Niue. Among non-island countries, the Czech Republic has the highest GDP share attributable to diabetes at 0.5%, followed by the United States at 0.4% and Germany at 0.4%. Ireland, Monaco and Bermuda face the three largest per capita economic burdens at INT$18,000, INT$12,000 and INT$8,000, respectively. Globally, the macroeconomic burden of diabetes mellitus is estimated to be INT$10.2 trillion at a discount rate of 2%, INT$8.3 trillion at a discount rate of 3% and INT$15.5 trillion without discounting. We provide country-specific estimates for the additional macroeconomic burden of diabetes mellitus due to COVID-19 infection in Supplementary Table 5. This analysis is entirely separate from the main results presented in Table 1. The additional cases and burden shown in Supplementary Table 5 are not included in the baseline projections. The COVID-19 analysis covers only the impact of infections that were reported between 1 January 2020 and 1 September 2022. The results without informal care and with discounting at 0% are presented in Supplementary Table 6; the results without informal care and with discounting at 3% are presented in Supplementary Table 7 and the results with an average weekly loss of four informal care hours (ranging from 0.285 to 8.3 h) are presented in Supplementary Table 8.

**Table 1:** Total macroeconomic burden, per capita economic burden and economic burden as a percentage of GDP in 2020–2050 attributable to diabetes mellitus by country and World Bank region in 2017 international dollars

Region	Country	Economic burden in millions of 2017 INT$	Percentage of total GDP in 2020–2050	Per capita loss in 2017 INT$
East Asia and Pacific	American Samoa^a^	111 (82–152)	0.472 (0.349–0.644)	2,041 (1,508–2,787)
East Asia and Pacific	Australia	72,053 (57,383–91,170)	0.177 (0.141–0.224)	2,458 (1,957–3,110)
East Asia and Pacific	Brunei Darussalam	2,368 (1,548–3,473)	0.315 (0.206–0.462)	4,977 (3,254–7,299)
East Asia and Pacific	Cambodia	8,314 (5,505–12,275)	0.206 (0.137–0.305)	425 (281–628)
East Asia and Pacific	China	1,596,436 (1,252,663–2,041,408)	0.163 (0.128–0.208)	1,103 (866–1,411)
East Asia and Pacific	Fiji	2,052 (1,249–3,270)	0.632 (0.385–1.008)	2,068 (1,259–3,294)
East Asia and Pacific	Guam^a^	516 (369–716)	0.249 (0.178–0.345)	2,804 (2,006–3,891)
East Asia and Pacific	Indonesia	458,610 (309,003–656,025)	0.314 (0.211–0.449)	1,493 (1,006–2,136)
East Asia and Pacific	Japan	251,143 (197,907–316,459)	0.195 (0.154–0.245)	2,150 (1,694–2,709)
East Asia and Pacific	Kiribati^a^	47 (33–66)	0.548 (0.385–0.760)	320 (225–443)
East Asia and Pacific	North Korea^a^	2,253 (1,603–3,144)	0.157 (0.112–0.220)	85 (60–118)
East Asia and Pacific	South Korea	181,335 (143,157–234,091)	0.247 (0.195–0.318)	3,621 (2,859–4,675)
East Asia and Pacific	Lao PDR	5,049 (2,983–7,948)	0.181 (0.107–0.285)	591 (349–931)
East Asia and Pacific	Malaysia	60,918 (40,996–89,078)	0.166 (0.112–0.243)	1,640 (1,103–2,398)
East Asia and Pacific	Marshall Islands^a^	34 (23–48)	0.456 (0.315–0.644)	500 (346–707)
East Asia and Pacific	Micronesia, Fed. Sts.^a^	63 (37–98)	0.595 (0.350–0.928)	483 (284–754)
East Asia and Pacific	Mongolia	1,138 (763–1,681)	0.070 (0.047–0.103)	292 (196–432)
East Asia and Pacific	Myanmar^a^	25,390 (20,625–32,528)	0.241 (0.196–0.309)	427 (347–548)
East Asia and Pacific	Nauru^a^	15 (10–23)	0.355 (0.240–0.541)	1,380 (934–2,105)
East Asia and Pacific	New Zealand	13,803 (10,590–17,657)	0.181 (0.139–0.231)	2,615 (2,006–3,345)
East Asia and Pacific	Northern Mariana Islands^a^	163 (119–222)	0.367 (0.267–0.499)	2,657 (1,938–3,615)
East Asia and Pacific	Palau^a^	50 (35–68)	0.697 (0.494–0.959)	2,714 (1,923–3,734)
East Asia and Pacific	Papua New Guinea^a^	4,174 (3,007–5,847)	0.309 (0.223–0.433)	360 (259–504)
East Asia and Pacific	Philippines	98,422 (67,470–134,048)	0.210 (0.144–0.286)	763 (523–1,040)
East Asia and Pacific	Samoa^a^	125 (90–174)	0.318 (0.228–0.441)	539 (387–749)
East Asia and Pacific	Singapore	32,334 (26,029–41,916)	0.173 (0.140–0.225)	5,141 (4,138–6,664)
East Asia and Pacific	Solomon Islands^a^	247 (183–330)	0.453 (0.337–0.606)	253(188–339)
East Asia and Pacific	Taiwan (Province of China)^a^	99,282 (69,418–144,889)	0.243 (0.170–0.354)	4,206 (2,941–6,138)
East Asia and Pacific	Thailand	66,850 (46,614–94,092)	0.161 (0.113–0.227)	966 (674–1,360)
East Asia and Pacific	Timor-Leste^a^	218 (149–305)	0.161 (0.110–0.225)	130 (89–181)
East Asia and Pacific	Tonga^a^	78 (56–108)	0.384 (0.277–0.531)	646 (465–893)
East Asia and Pacific	Tuvalu^a^	12 (8–18)	0.471 (0.323–0.670)	889 (610–1,264)
East Asia and Pacific	Vanuatu^a^	91 (64–127)	0.306 (0.216–0.429)	213 (150–297)
East Asia and Pacific	Vietnam	120,486 (76,006–181,368)	0.239 (0.151–0.360)	1,144 (722–1,723)
Europe and Central Asia	Albania	1,603 (1,040–2,363)	0.120 (0.078–0.177)	595 (386–877)
Europe and Central Asia	Andorra^a^	192 (138–266)	0.161 (0.116–0.223)	2,478 (1,774–3,424)
Europe and Central Asia	Armenia	3,699 (2,807–4,878)	0.245 (0.186–0.323)	1,265 (960–1,668)
Europe and Central Asia	Austria	19,837 (16,078–24,828)	0.148 (0.120–0.186)	2,166 (1,755–2,710)
Europe and Central Asia	Azerbaijan	6,395 (4,306–9,465)	0.169 (0.114–0.251)	591 (398–875)
Europe and Central Asia	Belarus	3,098 (2,190–4,406)	0.074 (0.052–0.105)	341 (241–485)
Europe and Central Asia	Belgium	25,055 (19,527–32,896)	0.157 (0.122–0.206)	2,091 (1,629–2,745)
Europe and Central Asia	Bosnia and Herzegovina	5,167 (3,656–7,266)	0.312 (0.221–0.439)	1,715 (1,213–2,411)
Europe and Central Asia	Bulgaria	19,645 (14,040–27,563)	0.362 (0.259–0.508)	3,194 (2,282–4,481)
Europe and Central Asia	Croatia	7,008 (5,033–9,824)	0.183 (0.131–0.257)	1,870 (1,343–2,621)
Europe and Central Asia	Cyprus	2,453 (2,035–3,014)	0.170 (0.141–0.209)	1,895 (1,572–2,328)
Europe and Central Asia	Czech Republic	76,846 (58,329–101,304)	0.525 (0.398–0.691)	7,202 (5,466–9,494)
Europe and Central Asia	Denmark	17,385 (13,620–22,273)	0.171 (0.134–0.219)	2,870 (2,249–3,677)
Europe and Central Asia	Estonia	3,811 (2,816–5,217)	0.217 (0.161–0.297)	3,054 (2,257–4,180)
Europe and Central Asia	Finland	16,263 (12,962–20,538)	0.218 (0.174–0.275)	2,929 (2,335–3,700)
Europe and Central Asia	France	85,473 (65,820–111,126)	0.107 (0.083–0.139)	1,277 (983–1,660)
Europe and Central Asia	Georgia	2,714 (2,087–3,547)	0.124 (0.095–0.162)	720 (554–941)
Europe and Central Asia	Germany	479,744 (370,644–616,536)	0.400 (0.309–0.514)	5,821 (4,497–7,481)
Europe and Central Asia	Greece	7,996 (5,910–10,778)	0.103 (0.076–0.139)	823 (608–1,109)
Europe and Central Asia	Greenland^a^	131 (94–183)	0.152 (0.108–0.212)	2,344 (1,670–3,276)
Europe and Central Asia	Hungary	28,614 (20,664–39,480)	0.245 (0.177–0.339)	3,142 (2,269–4,336)
Europe and Central Asia	Iceland	1,147 (809–1,601)	0.165 (0.116–0.230)	3,151 (2,224–4,401)
Europe and Central Asia	Ireland	98,570 (79,332–124,650)	0.254 (0.204–0.321)	18,409 (14,816–23,280)
Europe and Central Asia	Italy	101,235 (80,991–127,353)	0.168 (0.135–0.212)	1,748 (1,398–2,199)
Europe and Central Asia	Kazakhstan	25,682 (18,746–35,080)	0.155 (0.113–0.212)	1,195 (872–1,632)
Europe and Central Asia	Kyrgyz Republic	1,598 (1,109–2,258)	0.140 (0.097–0.197)	203 (141–287)
Europe and Central Asia	Latvia	3,605 (2,523–5,191)	0.184 (0.129–0.265)	2,171 (1,520–3,127)
Europe and Central Asia	Lithuania	5,752 (4,489–7,478)	0.154 (0.120–0.200)	2,404 (1,876–3,125)
Europe and Central Asia	Luxembourg	3,102 (2,351–4,132)	0.130 (0.098–0.173)	4,338 (3,287–5,778)
Europe and Central Asia	Moldova	1,848 (1,342–2,549)	0.152 (0.110–0.209)	493 (358–681)
Europe and Central Asia	Monaco^a^	498 (358–676)	0.181 (0.130–0.246)	11,646 (8,367–15,787)
Europe and Central Asia	Montenegro	1,234 (848–1,765)	0.304 (0.209–0.436)	2,007 (1,379–2,872)
Europe and Central Asia	The Netherlands	52,008 (41,283–66,014)	0.180 (0.143–0.229)	2,994 (2,377–3,801)
Europe and Central Asia	North Macedonia	2,705 (1,817–3,999)	0.246 (0.165–0.363)	1,354 (909–2,001)
Europe and Central Asia	Norway	21,969 (18,412–26,289)	0.217 (0.182–0.260)	3,628 (3,041–4,341)
Europe and Central Asia	Poland	138,843 (105,109–184,207)	0.282 (0.214–0.374)	3,861 (2,923–5,122)
Europe and Central Asia	Portugal	15,798 (12,492–20,305)	0.157 (0.125–0.202)	1,627 (1,287–2,091)
Europe and Central Asia	Romania^a^	41,416 (30,039–57,242)	0.183 (0.133–0.253)	2,328 (1,689–3,218)
Europe and Central Asia	Russian Federation	84,895 (61,901–112,453)	0.092 (0.067–0.122)	601 (439–797)
Europe and Central Asia	San Marino^a^	100 (71–138)	0.175 (0.125–0.242)	2,914 (2,078–4,029)
Europe and Central Asia	Serbia	15,391 (11,069–21,972)	0.320 (0.230–0.457)	1,936 (1,392–2,764)
Europe and Central Asia	Slovak Republic	15,211 (10,784–21,630)	0.262 (0.186–0.372)	2,878 (2,040–4,092)
Europe and Central Asia	Slovenia	4,870 (3,627–6,525)	0.170 (0.127–0.228)	2,405 (1,791–3,222)
Europe and Central Asia	Spain	105,744 (83,054–138,147)	0.199 (0.156–0.260)	2,319 (1,822–3,030)
Europe and Central Asia	Sweden	33,086 (27,968–39,409)	0.195 (0.165–0.232)	3,064 (2,590–3,650)
Europe and Central Asia	Switzerland	38,192 (29,438–49,869)	0.221 (0.170–0.288)	4,093 (3,155–5,344)
Europe and Central Asia	Tajikistan	5,803 (4,020–7,922)	0.288 (0.199–0.393)	455 (315–621)
Europe and Central Asia	Turkey	106,435 (80,423–143,045)	0.105 (0.079–0.141)	1,164 (880–1,564)
Europe and Central Asia	Turkmenistan^a^	4,625 (3,104–6,615)	0.182 (0.122–0.261)	654 (439–936)
Europe and Central Asia	Ukraine	1,122 (750–1,597)	0.039 (0.026–0.055)	28 (19–40)
Europe and Central Asia	United Kingdom	232,114 (181,449–295,839)	0.278 (0.218–0.355)	3,253 (2,543–4,146)
Europe and Central Asia	Uzbekistan	40,162 (25,680–59,209)	0.307 (0.196–0.452)	1,035 (662–1,526)
Latin America and Caribbean	Antigua and Barbuda^a^	210 (160–277)	0.344 (0.262–0.453)	1,975 (1,507–2,603)
Latin America and Caribbean	Argentina	37,302 (30,000–46,708)	0.163 (0.131–0.204)	739 (594–925)
Latin America and Caribbean	The Bahamas	1,090 (713–1,591)	0.334 (0.219–0.488)	2,502 (1,637–3,651)
Latin America and Caribbean	Barbados	447 (282–666)	0.464 (0.292–0.691)	1,561 (984–2,326)
Latin America and Caribbean	Belize	237 (163–335)	0.350 (0.240–0.494)	481 (330–678)
Latin America and Caribbean	Bolivia	6,719 (4,357–9,841)	0.201 (0.130–0.294)	484 (314–708)
Latin America and Caribbean	Brazil	96,068 (80,238–114,454)	0.132 (0.110–0.157)	427 (357–509)
Latin America and Caribbean	Chile	29,117 (23,713–36,369)	0.213 (0.174–0.266)	1,471 (1,198–1,837)
Latin America and Caribbean	Colombia	53,481 (38,509–74,911)	0.227 (0.163–0.317)	989 (712–1,385)
Latin America and Caribbean	Costa Rica	9,671 (6,878–13,461)	0.262 (0.186–0.364)	1,746 (1,242–2,430)
Latin America and Caribbean	Cuba^a^	13,223 (9,559–18,070)	0.270 (0.195–0.368)	1,214 (877–1,658)
Latin America and Caribbean	Dominica^a^	88 (64–121)	0.424 (0.306–0.583)	1,218 (877–1,674)
Latin America and Caribbean	Dominican Republic	32,941 (18,917–53,114)	0.344 (0.197–0.554)	2,741 (1,574–4,419)
Latin America and Caribbean	Ecuador	7,329 (5,591–9,855)	0.152 (0.116–0.205)	354 (270–476)
Latin America and Caribbean	El Salvador	4,209 (2,449–6,836)	0.258 (0.150–0.419)	618 (359–1,003)
Latin America and Caribbean	Grenada^a^	218 (169–280)	0.390 (0.302–0.502)	1,882 (1,458–2,422)
Latin America and Caribbean	Guatemala	13,117 (9,240–18,471)	0.242 (0.171–0.341)	579 (408–816)
Latin America and Caribbean	Guyana^a^	6,405 (4,315–9,403)	0.393 (0.265–0.577)	7,793 (5,249–11,440)
Latin America and Caribbean	Haiti^a^	2,082 (1,433–3,046)	0.238 (0.164–0.349)	157 (108–229)
Latin America and Caribbean	Honduras	3,977 (2,786–5,693)	0.203 (0.142–0.291)	330 (231–473)
Latin America and Caribbean	Jamaica	2,649 (1,627–3,973)	0.388 (0.238–0.582)	876 (538–1,314)
Latin America and Caribbean	Mexico	225,614 (169,697–291,183)	0.342 (0.257–0.442)	1,562 (1,175–2,016)
Latin America and Caribbean	Nicaragua^a^	2,401 (1,706–3,421)	0.217 (0.154–0.310)	313 (222–446)
Latin America and Caribbean	Panama	14,214 (10,676–18,999)	0.312 (0.234–0.417)	2,756 (2,070–3,684)
Latin America and Caribbean	Paraguay	7,048 (4,668–10,377)	0.228 (0.151–0.336)	856 (567–1,260)
Latin America and Caribbean	Peru	13,826 (9,986–19,597)	0.108 (0.078–0.153)	372 (269–528)
Latin America and Caribbean	Puerto Rico^a^	8,990 (6,364–12,901)	0.404 (0.286–0.580)	3,270 (2,315–4,692)
Latin America and Caribbean	St. Kitts and Nevis^a^	135 (98–183)	0.354 (0.257–0.480)	2,423 (1,759–3,283)
Latin America and Caribbean	St. Lucia^a^	288 (220–385)	0.465 (0.355–0.621)	1,542 (1,177–2,060)
Latin America and Caribbean	St. Vincent and the Grenadines^a^	212 (164–275)	0.500 (0.386–0.649)	1,894 (1,462–2,457)
Latin America and Caribbean	Suriname	663 (458–939)	0.335 (0.231–0.474)	1,030 (712–1,457)
Latin America and Caribbean	Trinidad and Tobago^a^	4,693 (2,800–6,990)	0.652 (0.389–0.970)	3,366 (2,008–5,013)
Latin America and Caribbean	Uruguay	2,274 (1,788–2,905)	0.109 (0.086–0.140)	634 (499–810)
Latin America and Caribbean	Venezuela, RB^a^	27,437 (17,167–40,978)	0.285 (0.178–0.425)	804 (503–1,201)
Latin America and Caribbean	Virgin Islands (US)^a^	609 (439–827)	0.439 (0.317–0.597)	6,280 (4,529–8,533)
Middle East and North Africa	Algeria^a^	25,050 (17,756–34,485)	0.181 (0.128–0.249)	474 (336–652)
Middle East and North Africa	Bahrain	5,032 (4,065–6,333)	0.218 (0.176–0.274)	2,420 (1,955–3,046)
Middle East and North Africa	Djibouti	399 (230–631)	0.127 (0.073–0.202)	344 (198–544)
Middle East and North Africa	Egypt, Arab Rep.	110,879 (76,605–159,894)	0.188 (0.130–0.272)	848 (586–1,222)
Middle East and North Africa	Iran, Islamic Rep.^a^	63,455 (47,662–80,305)	0.199 (0.150–0.252)	667 (501–845)
Middle East and North Africa	Iraq	23,533 (18,602–29,938)	0.168 (0.133–0.214)	424 (335–540)
Middle East and North Africa	Israel	30,378 (22,952–40,495)	0.213 (0.161–0.284)	2,848 (2,152–3,796)
Middle East and North Africa	Jordan	3,467 (2,547–4,879)	0.109 (0.080–0.154)	305 (224–429)
Middle East and North Africa	Kuwait	12,110 (10,383–14,470)	0.245 (0.210–0.293)	2,462 (2,111–2,942)
Middle East and North Africa	Lebanon	355 (211–550)	0.080 (0.047–0.124)	56 (33–86)
Middle East and North Africa	Libya^a^	20,081 (13,555–29,134)	0.196 (0.132–0.284)	2,564 (1,731–3,720)
Middle East and North Africa	Malta	3,548 (2,966–4,357)	0.327 (0.274–0.402)	8,035 (6,717–9,866)
Middle East and North Africa	Morocco	12,921 (9,843–17,504)	0.144 (0.110–0.195)	306 (233–415)
Middle East and North Africa	Oman	9,062 (5,798–13,439)	0.193 (0.123–0.286)	1,476 (944–2,189)
Middle East and North Africa	Qatar	10,650 (8,839–13,204)	0.147 (0.122–0.183)	3,089 (2,564–3,830)
Middle East and North Africa	Saudi Arabia	104,733 (86,956–129,349)	0.220 (0.182–0.271)	2,581 (2,143–3,188)
Middle East and North Africa	Syrian Arab Republic^a^	3,170 (2,190–4,591)	0.176 (0.122–0.255)	115 (80–167)
Middle East and North Africa	Tunisia	6,557 (5,078–8,452)	0.197 (0.153–0.254)	505 (391–651)
Middle East and North Africa	United Arab Emirates^a^	38,436 (25,303–57,270)	0.179 (0.118–0.267)	3,659 (2,409–5,452)
Middle East and North Africa	Yemen, Rep.^a^	2,935 (1,987–4,262)	0.111 (0.075–0.162)	75 (50–108)
North America	Bermuda^a^	489 (363–663)	0.261 (0.194–0.354)	8,338 (6,188–11,299)
North America	Canada	88,666 (68,587–114,225)	0.172 (0.133–0.221)	2,110 (1,632–2,718)
North America	United States	2,505,656 (2,148,139–2,934,496)	0.403 (0.346–0.472)	7,013 (6,012–8,213)
South Asia	Afghanistan^a^	2,720 (1,731–3,991)	0.152 (0.097–0.223)	52 (33–76)
South Asia	Bangladesh	68,195 (42,329–105,684)	0.125 (0.078–0.194)	374 (232–580)
South Asia	Bhutan	666 (400–1,047)	0.178 (0.107–0.279)	778 (467–1,222)
South Asia	India	1,010,578 (710,498–1,392,033)	0.201 (0.141–0.277)	657 (462–905)
South Asia	Maldives	438 (303–629)	0.132 (0.091–0.189)	802 (555–1,151)
South Asia	Nepal	8,582 (5,800–12,365)	0.162 (0.109–0.233)	255 (173–368)
South Asia	Pakistan	89,631 (57,645–132,205)	0.204 (0.131–0.301)	318 (205–469)
South Asia	Sri Lanka	36,990 (21,781–62,906)	0.379 (0.223–0.645)	1,684 (991–2,863)
Sub-Saharan Africa	Angola	5,143 (3,026–8,134)	0.104 (0.061–0.164)	97 (57–153)
Sub-Saharan Africa	Benin	1,767 (1,058–2,829)	0.085 (0.051–0.137)	99 (59–158)
Sub-Saharan Africa	Botswana	1,914 (1,432–2,576)	0.156 (0.117–0.210)	646 (484–870)
Sub-Saharan Africa	Burkina Faso	3,038 (1,917–4,524)	0.129 (0.081–0.192)	96 (61–144)
Sub-Saharan Africa	Burundi	180 (117–269)	0.071 (0.046–0.106)	10 (6–15)
Sub-Saharan Africa	Cabo Verde	146 (98–212)	0.116 (0.078–0.168)	232 (156–337)
Sub-Saharan Africa	Cameroon	3,987 (2,407–6,429)	0.095 (0.057–0.153)	105 (63–169)
Sub-Saharan Africa	Central African Republic^a^	274 (193–396)	0.152 (0.107–0.220)	42 (29–60)
Sub-Saharan Africa	Chad^a^	692 (476–992)	0.105 (0.072–0.151)	28 (19–40)
Sub-Saharan Africa	Comoros	105 (60–167)	0.113 (0.064–0.179)	90 (51–143)
Sub-Saharan Africa	Congo, Dem. Rep.	6,344 (3,911–9,776)	0.130 (0.080–0.200)	46 (28–70)
Sub-Saharan Africa	Congo, Rep.	437 (261–696)	0.126 (0.075–0.200)	55 (33–87)
Sub-Saharan Africa	Cote d’Ivoire	6,716 (4,168–10,299)	0.076 (0.047–0.116)	176 (109–270)
Sub-Saharan Africa	Equatorial Guinea^a^	438 (300–626)	0.139 (0.095–0.199)	208 (142–297)
Sub-Saharan Africa	Eritrea^a^	455 (313–659)	0.131 (0.090–0.190)	97 (66–140)
Sub-Saharan Africa	Eswatini	440 (263–681)	0.149 (0.089–0.230)	311 (186–482)
Sub-Saharan Africa	Ethiopia	15,176 (9,962–22,439)	0.071 (0.046–0.105)	95 (62–140)
Sub-Saharan Africa	Gabon	1,271 (873–1,869)	0.124 (0.085–0.182)	422 (290–621)
Sub-Saharan Africa	The Gambia	206 (124–330)	0.082 (0.049–0.132)	57 (34–92)
Sub-Saharan Africa	Ghana	12,670 (7,648–20,297)	0.153 (0.092–0.245)	306 (185–490)
Sub-Saharan Africa	Guinea	2,120 (1,281–3,355)	0.094 (0.057–0.149)	110 (66–174)
Sub-Saharan Africa	Guinea-Bissau	198 (128–296)	0.120 (0.077–0.179)	72 (47–108)
Sub-Saharan Africa	Kenya	7,944 (5,583–11,085)	0.072 (0.050–0.100)	109 (77–152)
Sub-Saharan Africa	Lesotho	274 (163–429)	0.232 (0.138–0.362)	114 (68–178)
Sub-Saharan Africa	Liberia^a^	250 (170–365)	0.118 (0.080–0.173)	35 (24–51)
Sub-Saharan Africa	Madagascar	1,367 (832–2,091)	0.088 (0.053–0.134)	34 (21–52)
Sub-Saharan Africa	Malawi^a^	1,342 (931–1,924)	0.113 (0.078–0.162)	48 (33–68)
Sub-Saharan Africa	Mali	1,430 (858–2,314)	0.069 (0.041–0.111)	46 (27–74)
Sub-Saharan Africa	Mauritania	568 (354–896)	0.053 (0.033–0.084)	84 (52–133)
Sub-Saharan Africa	Mauritius	4,245 (3,271–5,625)	0.512 (0.394–0.678)	3,394 (2,615–4,497)
Sub-Saharan Africa	Mozambique	2,388 (1,648–3,530)	0.131 (0.090–0.193)	50 (35–75)
Sub-Saharan Africa	Namibia	555 (375–828)	0.096 (0.065–0.144)	171 (115–254)
Sub-Saharan Africa	Niger	1,163 (754–1,767)	0.065 (0.042–0.099)	27 (18–41)
Sub-Saharan Africa	Nigeria	11,593 (7,897–16,880)	0.038 (0.026–0.056)	39 (26–57)
Sub-Saharan Africa	Rwanda	2,150 (1,274–3,397)	0.119 (0.070–0.188)	120 (71–189)
Sub-Saharan Africa	São Tomé and Principe^a^	36 (25–52)	0.110 (0.076–0.157)	120 (83–173)
Sub-Saharan Africa	Senegal	3,761 (2,582–5,450)	0.116 (0.079–0.168)	154 (105–222)
Sub-Saharan Africa	Seychelles^a^	296 (215–405)	0.297 (0.215–0.406)	2,876 (2,085–3,927)
Sub-Saharan Africa	Sierra Leone	170 (102–268)	0.046 (0.028–0.073)	16 (10–26)
Sub-Saharan Africa	Somalia^a^	849 (588–1,231)	0.119 (0.083–0.173)	34 (24–50)
Sub-Saharan Africa	South Africa	28,946 (23,201–35,902)	0.147 (0.118–0.182)	424 (340–525)
Sub-Saharan Africa	South Sudan^a^	828 (569–1,190)	0.116 (0.079–0.166)	54 (37–77)
Sub-Saharan Africa	Sudan	5,916 (4,128–8,490)	0.113 (0.079–0.162)	96 (67–137)
Sub-Saharan Africa	Tanzania	11,962 (6,883–19,256)	0.130 (0.075–0.209)	130 (75–210)
Sub-Saharan Africa	Togo	979 (598–1,545)	0.108 (0.066–0.170)	84 (51–132)
Sub-Saharan Africa	Uganda	6,371 (3,848–10,034)	0.121 (0.073–0.191)	95 (57–149)
Sub-Saharan Africa	Zambia	2,589 (1,538–4,120)	0.118 (0.070–0.188)	92 (55–147)
Sub-Saharan Africa	Zimbabwe	1,506 (964–2,257)	0.102 (0.066–0.154)	78 (50–117)
Others	Cook Islands^a^	64 (48–85)	0.593 (0.444–0.795)	3,654 (2,735–4,898)
Others	Niue^a^	3 (2–3)	0.711 (0.508–0.965)	1,505 (1,075–2,041)
Others	Palestine^a^	1,964 (1,501–2,589)	0.212 (0.162–0.279)	282 (215–372)
Others	Tokelau^a^	1 (1–2)	0.426 (0.294–0.589)	798 (550–1,104)

^a^Results for countries were imputed due to missing data.

Uncertainty intervals in parentheses were calculated based on the lower and upper bounds of 95% uncertainty intervals for GBD mortality and morbidity data.

**Fig. 1:**
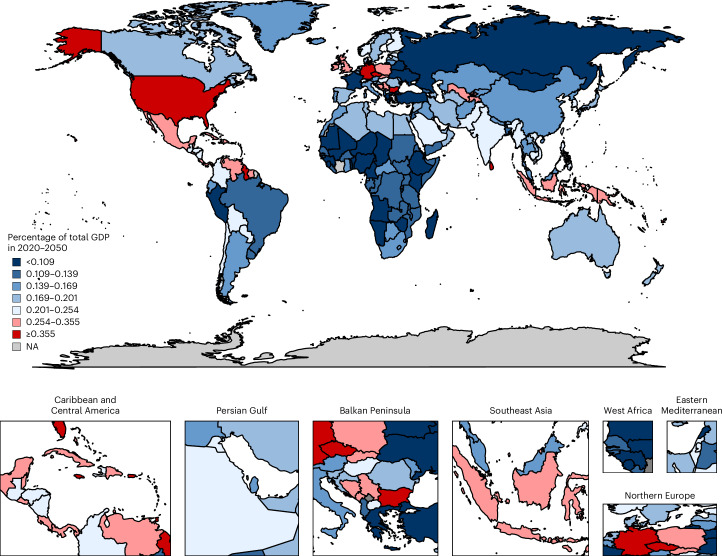
Macroeconomic burden of diabetes mellitus as a percentage of total GDP in 2020–2050. The map shows the projected macroeconomic burden of diabetes mellitus expressed as a percentage of GDP for 204 countries and territories from 2020 to 2050, based on a health-augmented macroeconomic model. Burden estimates reflect losses in effective labor supply due to mortality and morbidity and the diversion of treatment resources from savings and investment. Countries are shaded according to burden levels, with darker colors indicating higher relative losses. The highest relative burdens (≥0.355% of GDP) are concentrated in small island states and high-income countries such as American Samoa and Australia, whereas much of Africa and South Asia show lower relative burdens (<0.109%). Regional labels (for example, Caribbean and Central America, Persian Gulf, Balkan Peninsula, West Africa and Northern Europe) are included for orientation. NA indicates countries or territories without sufficient data. This figure illustrates the unequal distribution of diabetes-related economic losses across world regions. Source data

**Fig. 2:**
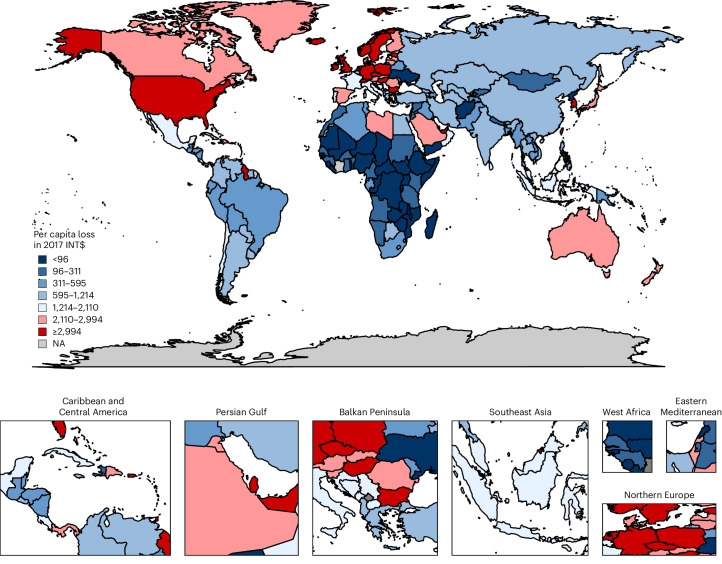
Per capita economic burden of diabetes mellitus in 2020–2050 (in 2017 INT$). The map displays the projected per capita economic burden of diabetes mellitus in 204 countries and territories from 2020 to 2050, expressed in 2017 international dollars adjusted for purchasing power. Darker shades indicate higher per capita losses. The highest burdens (≥INT$2,994 per person) are observed in countries including American Samoa, Australia and Brunei Darussalam, highlighting the substantial impact on individuals in high-income and island economies. Intermediate burden levels (INT$595–INT$2,110 per person) are observed across parts of Europe, the Middle East and Southeast Asia, while many low-income countries, particularly in Africa, fall below INT$96 per person. Regional labels provide geographic orientation, and NA indicates countries or territories with missing data. By showing the burden on a per-person basis, this figure underscores stark inequalities in the economic consequences of diabetes across populations. Source data

Figures 1 and 2 depict the macroeconomic burden for all 204 countries as maps without informal care. Figure 1 shows the burden for each country as a percentage of GDP. Figure 2 shows the absolute macroeconomic burden for each country. The deeper a country’s hue on the map, the greater is its economic burden in terms of the specific measure shown. Supplementary Figs. 5 and 6 display the results for 134 countries of the additional diabetes mellitus burden due to the effects of the COVID-19 pandemic.

Table 2 shows the aggregated results for World Bank regions and income groups. The economic burden of diabetes mellitus is equivalent to an annual tax of 0.22% on global GDP, or INT$1,157 per capita, in 2020–2050. North America faces the highest total economic burden among all World Bank regions, amounting to a yearly tax of 0.385% in 2020–2050. Latin America and Caribbean has the second-highest economic burden, accounting for 0.229% of the annual adjusted GDP projection over the period, followed by Europe and Central Asia at 0.221%. Diabetes mellitus imposes a substantial total macroeconomic impact in all World Bank regions. East Asia and Pacific has the highest overall burden of INT$3.1 trillion, followed by North America with a second-highest burden of INT$2.6 trillion and Europe and Central Asia rank third with a total loss of INT$2.0 trillion. The per capita burden of diabetes mellitus ranges from INT$99 in Sub-Saharan Africa to INT$6,497 in North America. Supplementary Tables 9–11 show more aggregated results for World Bank regions and income groups with discount rates of 0% in Supplementary Table 9 and 3% in Supplementary Table 10 and different informal care hours in Supplementary Table 11.

**Table 2:** Total macroeconomic burden, economic burden as a percentage of total GDP in 2020–2050 and per capita economic burden attributable to diabetes mellitus mortality and morbidity by World Bank region and by World Bank income group in 2017 INT$

Region/income group	Economic burden in billions of 2017 INT$	Percentage of total GDP in 2020–2050	Per capita loss in 2017 INT$
By region			
East Asia and Pacific	3,104 (2,336–4,115)	0.190 (0.143–0.252)	1,261 (949–1,672)
Europe and Central Asia	2,018 (1,549–2,633)	0.211 (0.162–0.275)	2,168 (1,665–2,829)
Latin America and Caribbean	629 (467–837)	0.229 (0.170–0.305)	876 (651–1,166)
Middle East and North Africa	487 (364–654)	0.192 (0.144–0.258)	866 (647–1,163)
North America	2,595 (2,217–3,049)	0.385 (0.329–0.453)	6,497 (5,551–7,635)
South Asia	1,218 (840–1,711)	0.197 (0.136–0.276)	577 (398–811)
Sub-Saharan Africa	163 (109–239)	0.097 (0.065–0.142)	99 (67–146)
By income group			
Low income	65 (42–97)	0.101 (0.066–0.152)	67 (44–101)
Lower-middle income	2,296 (1,571–3,251)	0.206 (0.141–0.292)	583 (399–826)
Upper-middle income	2,673 (2,047–3,490)	0.166 (0.127–0.217)	1,009 (772–1,317)
High income	5,152 (4,206–6,360)	0.289 (0.236–0.357)	4,159 (3,395–5,133)
Total	10,216 (7,884–13,241)	0.223 (0.172–0.289)	1,157 (893–1,499)

Uncertainty intervals in parentheses were calculated based on the lower and upper bounds of 95% uncertainty intervals for GBD mortality and morbidity data.

### Comparison with health burden measured in disability-adjusted life years

Table 3 compares the global distribution of economic losses and the lifetime disease burden of diabetes mellitus in disability-adjusted life years (DALYs). East Asia and Pacific shoulders both the largest economic burden and the largest disease burden in terms of DALYs, accounting for 30.39% of the total global economic loss and 28.81% of DALYs in 2020. South Asia is projected to have the largest disease burden, accounting for approximately 30.22% of total DALYs in 2050. North America has the largest per capita economic loss, accounting for 25.40% of the total economic burden, despite being home to only 4.52% of the annual adjusted global population between 2020 and 2050. The per capita economic burden of diabetes mellitus (column 2 divided by column 6) and the DALYs rate (column 3 divided by column 6) are much higher in high-income countries than in other countries. In low and lower-middle-income countries, increasing DALYs from diabetes mellitus will cause a high economic burden in the future.

**Table 3:** Comparison of macroeconomic loss (measured in 2017 INT$) and lifetime disease burden (measured in DALYs) by World Bank region and World Bank income group

Region/income group	Economic cost in billions of 2017 INT$	DALYs in millions in 2020	DALYs in millions in 2050	Annual GDP in billions 2020–2050	Annual population in millions 2020–2050
By region					
East Asia and Pacific	3,104 (30.39%)	21 (28.81%)	38 (23.25%)	52,763 (35.70%)	2,461 (27.87%)
Europe and Central Asia	2,017 (19.75%)	9 (12.75%)	15 (9.06%)	30,825 (20.86%)	930 (10.54%)
Latin America and Caribbean	629 (6.16%)	9 (12.59%)	22 (13.66%)	8,869 (6.00%)	718 (8.13%)
Middle East and North Africa	487 (4.77%)	4 (5.65%)	14 (8.70%)	8,163 (5.52%)	562 (6.36%)
North America	2,594 (25.40%)	5 (6.64%)	7 (4.16%)	21,720 (14.70%)	399 (4.52%)
South Asia	1,218 (11.92%)	18 (24.53%)	49 (30.22%)	19,983 (13.52%)	2,111 (23.90%)
Sub-Saharan Africa	163 (1.60%)	7 (8.96%)	18 (10.78%)	5,420 (3.67%)	1,642 (18.59%)
By income group					
Low income	65 (0.64%)	4 (4.99%)	12 (7.07%)	2,068 (1.40%)	962 (10.90%)
Lower-middle income	2,296 (22.48%)	32 (44.04%)	89 (54.92%)	35,950 (24.32%)	3,938 (44.59%)
Upper-middle income	2,672 (26.17%)	23 (31.68%)	40 (24.82%)	51,967 (35.16%)	2,650 (30.01%)
High income	5,151 (50.43%)	14 (18.64%)	20 (12.29%)	57,446 (38.87%)	1,238 (14.03%)
Sum	10,214 (100.00%)	73 (100.00%)	163 (100.00%)	147,771 (99.98%)	8,830 (99.99%)

Each country is classified into a World Bank region as in Table 1. The seven World Bank regions do not include the Cook Islands, Niue, Palestine and Tokelau. Uncertainty intervals in parentheses were calculated based on the lower and upper bounds of 95% uncertainty intervals for GBD mortality and morbidity data.

### Contribution of treatment costs and human capital losses

**Fig. 3:**
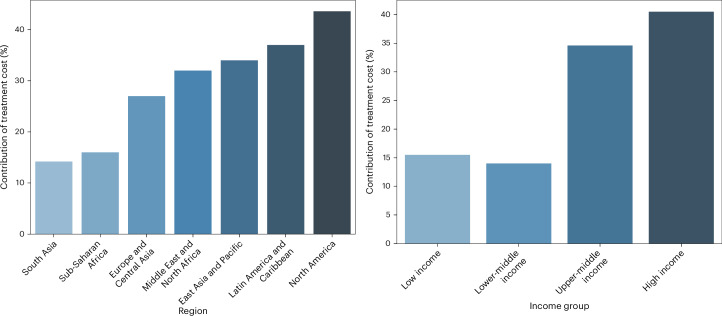
Contribution of treatment costs to the economic loss from diabetes mellitus by World Bank region and income group. The proportion of the total economic burden of diabetes mellitus attributable to direct treatment costs, based on a health-augmented macroeconomic model. Left, regional variation across seven global regions (South Asia, Sub-Saharan Africa, Europe and Central Asia, Middle East and North Africa, East Asia and Pacific, Latin America and Caribbean and North America). North America and Latin America and Caribbean show the highest contributions of treatment costs, while South Asia and Sub-Saharan Africa show much lower shares. Right, variation by World Bank income group (low-income, lower-middle-income, upper-middle-income and high-income countries). Treatment costs account for 40.5% of the total burden in high-income countries, compared with 14.0% in lower-middle-income countries. Together, these panels highlight structural disparities in healthcare financing, with direct medical expenditures weighing more heavily in wealthier countries, while labor productivity losses and informal caregiving dominate in lower-income settings. Source data

Figure 3 illustrates the decomposition of the economic burden of diabetes mellitus, isolating the contribution of treatment costs (physical capital). The residual burden, after accounting for treatment costs, reflects losses in human capital due to diabetes-related morbidity and mortality. Informal care costs are excluded from this analysis. Our results show that treatment costs have a more important role in high-income countries than in low-income countries. In high-income countries, the drag on physical capital accumulation resulting from the diversion of savings to finance treatment accounts for approximately 40.5% of the total economic burden due to diabetes mellitus. This number declines to 34.6% for upper-middle-income countries, 15.5% for low-income countries and 14.0% for lower-middle-income countries. The treatment cost share of the total economic burden is highest in North America at 43.6%, whereas the share is 14.2% in South Asia.

### Impact of informal care on the economic burden

We also explored the importance of informal care in the economic burden of diabetes mellitus, as shown in Supplementary Fig. 7. Our results show that informal care has a large role in all regions and countries. When considering informal labor, its share of the economic burden ranges from 84.6% in Sub-Saharan Africa to 90.8% in South Asia. Informal care accounts for 86.8% of the economic burden in the high-income group, 86.2% in the upper-middle-income group, 88.5% in the lower-middle-income group and 84.5% in the low-income group. That informal care accounts for such a high share of the total economic burden of diabetes mellitus globally reflects the fact that diabetes mellitus prevalence exceeds mortality by a factor of 30–50, implying the existence of a large population with long-term chronic care needs. While our primary model does not explicitly simulate a scenario in which all undiagnosed individuals are instantaneously and costless diagnosed, we conducted a sensitivity analysis assuming that these individuals would incur the same treatment cost as those already diagnosed, without any immediate productivity gains. This scenario led to an increase in the estimated macroeconomic burden by 5–21% in Supplementary Fig. 8, depending on the prevalence of undiagnosed diabetes in each region^21^.

## Discussion

This study comprehensively considers economic adjustment mechanisms; productivity loss among people with different education and experience levels; and the effects of morbidity, informal care and treatment costs to estimate the global economic burden of diabetes mellitus. This approach was applied consistently to 204 countries and territories, allowing for comparisons across regions, income groups and countries. Our findings fill several knowledge gaps. First, our results suggest that, between 2020 and 2050, diabetes mellitus will cost the global economy 10.2 trillion (in 2017 INT$, with a discount rate of 2%), which is equivalent to an annual tax of 0.22% on global GDP or a per capita loss of $1,157. When considering the substantial labor loss due to informal care for diabetes mellitus, the total economic burden amounts to INT$78.8 trillion, which is equivalent to an annual tax of 1.72% on global GDP and more than six times the cost without considering informal care. Informal care has a major role in the economic burden of diabetes mellitus in all regions and countries. These results suggest that policymakers should pay particular attention to the enormous economic burden of diabetes mellitus caregiving. Second, this study provides an estimate of the macroeconomic burden of diabetes in all countries worldwide based on a rigorous methodology that accounts for economic adjustment mechanisms and reflects the fact that healthcare expenditures would otherwise be saved/invested. Third, our study shows that the health and economic burdens of diabetes mellitus are unevenly distributed across countries and regions.

In previous studies using the same macroeconomic model, the global economic burden of various diseases has been quantified, allowing a comparison with diabetes mellitus. For instance, the global burden of Alzheimer’s disease and other dementias is estimated at INT$14.5 trillion (INT$, 2020) between 2020 and 2050, accounting for 0.42% of annual global GDP. A substantial portion of this burden comes from informal caregiving, with lower-middle-income countries having 85.45% of the total burden attributed to caregiving^22^. In contrast, our study shows that the economic burden of diabetes mellitus is INT$10.2 trillion (INT$, 2017) during the same period (0.22% of GDP annually without informal care), and rises to INT$78.8 trillion when informal care is included, highlighting its wide-reaching impact globally. Similarly, the economic burden of road injuries between 2015 and 2030 is estimated at 1.8 trillion in 2010 US dollars, or 0.12% of annual GDP, with high-income countries bearing a large share through physical capital losses and healthcare costs^18^. This figure is far lower than the projected burden of diabetes mellitus, which combines the direct and indirect costs of a chronic condition with long-term social and economic consequences. For chronic obstructive pulmonary disease, the global economic burden from 2020 to 2050 is estimated at INT$5.8 trillion (INT$, 2017), equivalent to 0.12% of annual GDP^23^. While the treatment costs dominate in middle-income and high-income countries, productivity losses are more substantial in low-income countries. Diabetes mellitus imposes nearly double the economic burden of chronic obstructive pulmonary disease, further amplified when informal care is considered. Finally, the economic burden of 29 cancers worldwide is projected to be INT$25.2 trillion (INT$, 2017) from 2020 to 2050, equivalent to 0.45% of annual global GDP^24^. Although cancers encompass a broader range of conditions, diabetes mellitus, as a single chronic disease, accounts for nearly half of this burden when informal caregiving is excluded. This underscores the substantial and often underestimated macroeconomic impact of diabetes on global economies. The comparison across these diseases highlights the unique position of diabetes mellitus as a chronic disease with substantial global economic consequences. Its burden, driven by both direct costs and the often-overlooked informal caregiving component, underscores the urgent need for global collaborative efforts to mitigate its impact.

The global prevalence of diabetes mellitus and DALYs from type 2 diabetes mellitus are currently high and projected to rise in all regions and most countries, albeit with varying rates of increase and underlying causes. Across regions, East Asia and Pacific faced the highest health burden from diabetes mellitus in recent years, but this is predicted to shift to South Asia by 2050. Across income levels, middle-income countries, particularly those in the lower-middle-income category, have carried the highest health burden and face a noticeably increasing trend from 2020 to 2050. Studies have shown that modifiable risk factors such as high body mass index and dietary risks account for the greatest portion of attributable deaths and DALYs from diabetes among all risk factors included in the 2017 Global Burden of Disease (GBD) study^25^. In addition to countries with a high prevalence of risk factors, countries lacking quality healthcare—including health promotion, prevention, diagnosis, control and treatment—also tend to undergo greater health burdens of diabetes^3,26^. Type 2 diabetes is manageable and preventable, as suggested by the fact that the incidence of diabetes mellitus is declining in several countries^27,28^. Nevertheless, the prevalence of diabetes is still increasing even in these countries, and its increasing prevalence around the world presents considerable health and economic challenges, primarily due to the costs associated with long-term care and management^3^.

The results of this study underestimate the economic burden of diabetes mellitus because there are many undiagnosed patients. The IDF has estimated that 240 million people were living with undiagnosed diabetes globally in 2021, meaning that nearly half of adults with diabetes were unaware of their condition; notably, 90% individuals believed to be going through undiagnosed diabetes live in LMICs^3^. Moreover, many health systems in Sub-Saharan Africa continue to face high infectious disease burdens and are unable to cope with the growing burden of diabetes^11^. If these LMICs do not intervene with respect to risk factors for diabetes mellitus and improve their medical care, the growing diabetes mellitus epidemic may overwhelm their already struggling health systems^11^.

Regionally, North America, Latin America and Caribbean, and Europe and Central Asia show the largest economic burdens as a share of GDP in 2020–2050 due to having the highest DALY rates from diabetes mellitus. Among countries, American Samoa, Australia and Brunei Darussalam show the highest diabetes-related GDP burden globally, highlighting that both small economies and high-income countries can be especially vulnerable to chronic disease impacts. This pattern aligns with their elevated diabetes-related DALY rates and higher levels of productivity loss per health-adjusted life year. East Asia and Pacific, and North America face the greatest absolute economic burdens of diabetes mellitus. These are primarily driven by the size of their economies and populations—particularly China and the United States. The reasons for the high economic burdens observed in these countries differ. For China, the high economic burden of diabetes mellitus is mainly attributable to its large affected population; in 2021, China had the largest number of adults with diabetes mellitus, followed by India^3^. In contrast, the large economic burden of diabetes mellitus in the United States is primarily due to high treatment costs and high levels of physical capital diversion. In terms of income groups, although LMICs, which account for 86.0% of global population, bear a high health burden from diabetes, they account for only 49.57% of the global economic burden of the disease, reflecting lower average wages, less productive labor losses and constrained healthcare expenditures. In contrast, high-income countries bear a high economic burden which is disproportionate to their population size and disease burden. This may be due to their higher levels of education and productivity in the workforce—for the same loss of DALYs, the reduction in income is therefore greater. In addition, higher-income countries offer more comprehensive medical care for diabetes mellitus and have more advanced health systems, which implies higher input costs.

The share of informal care in the total economic burden of diabetes is high in all regions and countries, especially in LMICs, although its precise magnitude remains subject to substantial uncertainty. Because diabetes mellitus results in chronic morbidity for many patients, informal caregivers spend substantial time assisting with treatment, care (for example, glucose monitoring, diet and medication adherence), and support for functional limitations due to diabetes complications^29,30^. As the population ages, the number of people requiring daily help is expected to increase dramatically, potentially amplifying a rapid rise in the economic burden associated with informal caregiving^29,31^. While our results highlight the substantial macroeconomic impact of informal care, the estimates remain highly sensitive to key assumptions. The literature shows considerable variation in caregiving time specifically attributable to diabetes, as patients often require assistance due to age or other conditions. In addition, caregiver costs vary by country, sex and age due to differences in wage levels. Although our model adjusts for these factors—using subgroup-specific labor loss and including only the additional time due to diabetes—uncertainty persists. Still, even under conservative assumptions, such as four extra caregiving hours per week, informal care accounts for a substantial share of the total economic burden. Despite the high cost of informal care for diabetes mellitus, its associated economic burden has not been fully incorporated into economic assessments in previous studies, thereby underestimating the economic benefits of disease interventions^31^.

Our findings suggest that strengthening public health interventions to reduce the burden of diabetes is essential to protect global health and economic well-being. The World Health Organization has launched the Global Diabetes Compact, an initiative to improve diabetes prevention and care sustainably, with a focus on supporting LMICs with high numbers of diabetes deaths^32,33^. In addition to such initiatives, we recommend the following public health interventions to reduce the burden of diabetes. First, we need to strengthen lifestyle interventions. Studies show that 90% of type 2 diabetes cases could be avoided through adherence to lifestyle factors such as increased physical activity, consuming a healthy diet, maintaining a body mass index below 25 kg m^−2^ and avoiding smoking^34,35^. Second, we need to enhance cost-effective diabetes screening—for example, screening for prediabetes and undiagnosed diabetes in the general population^33^—and providing regular screening in diabetic patients for damage to the eyes, kidneys and feet to promote early treatment^36^. Third, we need to strengthen early diagnosis of symptomatic individuals and those with known risk factors. People with diabetes are often treated too late, and lifestyle interventions can be more effective if the disease is detected early^37^. Fourth, we need to focus on social causes of disease beyond patients’ control, such as humanitarian crises and food insecurity. In addition to instituting changes in policies that currently limit people’s access to healthy food and healthcare, the government should also provide social support and psychological services to help patients manage their symptoms and the stress of the disease^33^. Fifth, as access to new medications improves, particularly GLP-1 receptor agonists (such as Ozempic), the economic burden of diabetes may decrease. These drugs have demonstrated effectiveness in improving glycemic control and reducing cardiovascular risks. While currently more accessible in high-income settings, their broader adoption—especially as costs decrease—could lead to substantial public health and macroeconomic benefits globally.

Our model has several limitations. First, we used the diabetes mellitus-related health expenditure data provided in ref. ^38^, which may overestimate or underestimate the cost of diabetes mellitus treatment. Second, due to the lack of data, we used linear regression to impute the economic burden of diabetes mellitus for 60 of 204 countries and territories. However, because the countries for which we imputed costs represent only 7.3% of the global population, this does not substantially affect our results. Third, we did not include the burden of undiagnosed diabetes mellitus cases, which are estimated to represent about 44.7% of total diabetes mellitus cases^3^. Fourth, we did not account for mortality resulting indirectly from diabetes mellitus. Because diabetes mellitus is a cause of other conditions, such as cardiovascular diseases, we consequently underestimate the economic burden of diabetes mellitus. For a more detailed discussion of the strengths and limitations of this study, see Supplementary Table 12.

The worldwide macroeconomic burden of diabetes mellitus is substantial, amounting to 0.22% of GDP annually or 1.72% of GDP if informal care is considered. The economic and health burdens of diabetes mellitus are distributed unequally. Across regions, North America bears the largest economic burden, at 0.39% of GDP, followed by Latin America and the Caribbean at 0.23%, and Europe and Central Asia at 0.21%. Our study emphasizes the critical need for investment in global efforts to prevent and mitigate diabetes mellitus.

## Methods

This study complies with all relevant ethical regulations. The analyses were conducted using aggregated, publicly available data from international repositories and previously published sources. No individual-level human or animal data were collected, and therefore, ethical approval from an institutional review board or ethics committee was not required.

### Model description

We estimated the macroeconomic burden of diabetes mellitus for 204 countries. The definition of diabetes mellitus followed the GBD study’s diabetes mellitus category^39^. Of the 204 studied countries, data from 144 were completed for our projections. We directly calculated the macroeconomic burden of diabetes mellitus for these 144 countries using the health macroeconomic model described in detail in the previous studies^15–20^. In this model, diabetes mellitus affects the economy through three main pathways. First, it reduces effective labor supply through mortality and morbidity. Diabetes mellitus deaths shrink the population, including working-age individuals, while diabetes mellitus morbidity reduces productivity and increases absenteeism. We adjust labor loss using age-specific and sex-specific labor force participation rates, reducing the potential for overestimation. Second, diabetes-related treatment costs reduce aggregate savings and investment by reallocating resources from capital accumulation to healthcare consumption. While reductions in such costs may boost investment, some resources may be redirected to other diseases, slightly overstating the net economic gains. Third, we estimate only the excess informal caregiving time caused by diabetes mellitus, excluding care related to coexisting conditions. This avoids overstating the informal care burden.

We estimated the additional cost associated with the rise in diabetes mellitus cases and increased mortality among patients with diabetes mellitus attributable to COVID-19. The number of COVID-19 cases was based on daily counts of individuals infected with COVID-19, as estimated by the Institute for Health Metrics and Evaluation^40^. We analyzed the long-term (2020–2050) impact of infections during the first 3 years of the pandemic—1 January 2020 to 1 September 2022—according to updated COVID-19 infection projections from the Institute for Health Metrics and Evaluation. To do so, we first derived the number of additional cases of diabetes based on the increased risk of incident diabetes in COVID-19 patients; a cohort study of 181,280 participants between 1 March 2020 and 30 September 2021 found an HR of 1.40 (95% CI = 1.36–1.44) for incident diabetes in people who survived the first 30 days of severe acute respiratory syndrome coronavirus 2 (SARS‑CoV‑2) infection relative to those who had not contracted SARS-CoV-2 (ref. ^8^). Then, we calculated the increased mortality rate among diabetic patients due to the increased risk of death from COVID-19 infection; a cohort study of 6,014 inpatients with diabetes—either COVID-19 positive (*n* = 698) or negative (*n* = 5,316)—revealed that diabetic patients hospitalized with COVID-19 were 3.6 times more likely to die than those not infected^7^. Finally, we estimated the macroeconomic cost associated with the increased mortality and morbidity of diabetes due to COVID-19. The projected long-term burden (2020–2050) reflects the elevated diabetes risk among individuals with prior COVID-19 infection from 2020 to 2022, who had a 40% higher incidence (HR = 1.40, 95% CI = 1.36–1.44) compared to controls.

Providing informal or unpaid care—which constitutes a substantial proportion of diabetes mellitus care—reduces the formal labor hours of caregivers. We considered the labor impact of informal care related to diabetes mellitus by subtracting the following estimate of effective labor from the labor supply for each diabetes mellitus patient. Specifically, we assumed informal care time as 4.0 h for each diabetes patient for each week, based on the estimation provided in ref. ^29^, and assumed that full-time employees work an average of 35.9 h per week, as reported by the International Labour Organization^41^. Consequently, for each patient with diabetes mellitus, the labor supply is reduced by 0.11 (4.0 divided by 35.9) units of labor due to informal caregiving. We also considered the detailed age distribution of informal caregivers to estimate the impact of informal labor loss on the macroeconomic burden. For sensitivity analyses, we revised our estimates of weekly informal caregiving hours. We set the lower bound at 0.285 h per week, calculated by multiplying the lowest reported disability prevalence among diabetic adults (15%^42^) by the conservative weekly caregiving time (1.9 h per week^29^) for individuals with mild diabetes. The upper bound remained at 8.3 h per week^29^, reflecting the higher caregiving needs observed among older populations with more severe diabetes. Formal caregiving is not considered an economic loss, as it involves paid labor and generates economic value. It is treated as part of the overall economy in our accounting framework.

To quantify the macroeconomic burden of diabetes mellitus, we compared aggregate output (using GDP) across three scenarios over the period 2020–2050: (1) the status quo scenario, in which no interventions are implemented that could reduce the mortality, morbidity, or prevalence of diabetes mellitus relative to current and projected rates; (2) a counterfactual scenario, in which we assumed the complete elimination of diabetes mellitus at zero cost; and (3) a COVID-19 scenario, in which we estimated the increased mortality and morbidity of diabetes mellitus due to COVID-19 between 1 January 2020 and 1 September 2022. The macroeconomic burden of diabetes mellitus was calculated as the cumulative difference in projected GDP between scenarios (1) and (2), which served as the baseline. Furthermore, because COVID-19 increases the incidence of, and mortality from, diabetes mellitus, we calculated the additional macroeconomic burden attributable to COVID-19 as the cumulative difference due to the increased diabetes mellitus cases between scenarios (2) and (3) during this period. We describe our counterfactual assumptions in detail below.

In the counterfactual scenario, we assume the complete elimination of diabetes mellitus starting in 2020, consistent with the comparative risk assessment framework adopted by the GBD study. In this scenario, all diabetes-related mortality and morbidity are fully averted, while risks from other causes remain unchanged. This approach facilitates consistent cause-specific attribution of economic burden but may overestimate benefits, especially among older adults with substantial competing mortality risks. In translating this health shock into economic outcomes, our health macroeconomic model assumes that eliminating diabetes would increase the effective labor supply by reducing disease-related absenteeism, presenteeism and premature mortality. It would also reduce healthcare expenditures for diabetes treatment, thereby boosting aggregate savings and physical capital accumulation through increased investment. These health-induced changes then generate downstream effects on GDP growth over time. We do not model general equilibrium feedbacks such as changes in wages, labor force participation preferences or government budget reallocation across sectors. Instead, we apply a partial equilibrium framework with fixed labor participation rates and savings behaviors, where changes stem only from shifts in the disease burden. As such, we provide a structured yet conservative estimate of the macroeconomic burden of diabetes mellitus. These estimates are based on a simulation model and should not be interpreted as precise causal effects; rather, they are indicative projections based on clearly defined and transparent assumptions.

### Data

We considered data for 204 countries and a set of World Bank regions. GDP projections for the status quo scenario, saving rates and health expenditures were taken from the World Bank’s database^43–45^. The mortality and morbidity data (years of life lost due to premature mortality and years lost due to disability) were obtained from the recently updated GBD 2021 (refs. ^39,46^). We relied on the International Labour Organization for age–sex-specific labor force projections^47^ and the Barro–Lee education database for age–sex-specific data on average years of schooling^48^. We obtained the age–sex-specific population from the Department of Economic and Social Affairs population dynamics database^49^. Using these data sources, we calculated human capital according to the Mincer equation^50^ and inferred the experience-related human capital component by relying on the corresponding estimates discussed in ref. ^51^. The physical capital data were taken from the Penn World Table projections^52^, and we followed standard economic estimates for the value of the output elasticity of physical capital (that is, the percentage change in output for a 1% change in the physical capital stock)^53^.

We used data to calculate treatment costs (ref. ^38^); these data include both inpatient and outpatient medical costs of diabetes mellitus. Supplementary Table 1 shows country-specific treatment data and Supplementary Table 2 shows other parameter values and data sources used in the macroeconomic model. To make country estimates comparable, all costs were converted to 2017 international dollars (2017 INT$). For 60 countries, some data—mostly on education, physical capital and the saving rate—were incomplete (see Supplementary Table 3 for details); reliable data on GDP and the prevalence rate of diabetes mellitus were available for these countries. Similar to the previous research^18^, we used a linear projection to approximate the economic burden of diabetes mellitus for these countries, which is shown in detail in Supplementary Table 4.

### Modeling details

The goal was to calculate the economic effect of diabetes mellitus due to healthcare expenses and productivity losses from death, morbidity and informal care. For each country, we performed the following analysis:

In step 1, we identified the disease burden of diabetes mellitus (in terms of mortality, morbidity and treatment costs).

In step 2, we constructed economic projections for the following two scenarios: a status quo scenario, in which GDP is projected to grow based on current estimates and projections of disease prevalence, and a counterfactual scenario, in which diabetes mellitus prevalence is eliminated from the beginning of the time frame. The economic projections use a macroeconomic production function and can be further decomposed into the following two parts:

1. Projections of effective labor supply; and
2. Projections of physical capital accumulation.

In step 3, we calculated the economic loss as the cumulative difference in projected annual GDP between these two scenarios for various discount rates.

In the counterfactual scenario where diabetes mellitus is eliminated, we assume that diabetes-related morbidity and mortality are fully averted, while the risks of morbidity and mortality from other causes remain unchanged. This assumption follows the GBD comparative risk assessment framework, allowing for consistent estimation across causes. However, it may overestimate the benefits of eliminating diabetes, particularly in older populations, due to unmodeled competing risks. This detailed model description follows our previous contributions, in which we applied the framework to estimate the economic burden of noncommunicable diseases in China, Japan and South Korea^15^, as well as in the United States and European countries^17,54^, and the economic burden of noncommunicable diseases and other risk factors^18^.

### Production function

Consider an economy in which time $t=\mathrm{1,2},\ldots ,\infty$ evolves discretely. Building upon the details in ref. ^55^, we considered the following production function for this economy:

$${Y}_{t}={A}_{t}{K}_{t}^{\alpha }{H}_{t}^{1-\alpha },$$

where ${Y}_{t}$ is aggregate output; ${A}_{t}$ is the technological level at time $t$, which we assumed evolves exogenously; ${K}_{t}$ is the physical capital stock (that is, machines, factory buildings, and so on); and ${H}_{t}$ represents aggregate human capital. The parameter $\alpha$ is the elasticity of final output with respect to physical capital. The aggregate production function recognizes that output is not only produced with physical capital and ‘raw labor’ as in the framework discussed in ref. ^56^, on which the original EPIC model is based^57^, but with ‘effective labor’, of which health is a crucial determinant.

Physical capital evolves according to

$${K}_{t+1}=\left(1-\delta \right){K}_{t}+{Y}_{t}-{C}_{t}-{\rm{TC}}_{t}=\left(1-\delta \right){K}_{t}+{s}_{t}{Y}_{t},$$

where $\delta$ refers to the depreciation rate, ${s}_{t}$ refers to the saving rate, ${\rm{TC}}_{t}$ refers to the costs of the ongoing treatment of diabetes mellitus and ${C}_{t}$ refers to the amount of consumption. From the above Equation, it follows that the saving rate is defined as

$${s}_{t}=1-\frac{{C}_{t}+{\rm{TC}}_{t}}{{Y}_{t}}.$$

Of note, aggregate output ${Y}_{t}$ is used for the following three purposes: (1) to pay treatment costs ${\rm{TC}}_{t}$ (hospitalization, medication, and so on), (2) to consume the amount ${C}_{t}$ and (3) to save.

Individuals of age group $a$ are endowed with ${h}_{t}^{(a)}$ units of human capital and supply ${{\mathcal{l}}}_{t}^{(a)}$ units of labor from the age of 15 up to their retirement at age $R$, that is, for $a\in [15,R]$. Children younger than 15 years of age and retirees older than $R$ do not work. *R* varies by country and could correspond to a high age (for example, some people aged above 80 years could also be working). In the theoretical derivations, *R* indicates the upper bound of the summation. In our simulations, we used labor projections data from the International Labour Organization, and positive values for the labor force exist for cohorts above the age of 65 years. Aggregate human capital in the production function (1) is then defined as the sum over the age-specific effective labor supply of each age group:

$${H}_{t}=\mathop{\sum }\limits_{a=15}^{R}{h}_{t}^{\left(a\right)}{{\mathcal{l}}}_{t}^{\left(a\right)}{n}_{t}^{\left(a\right)},$$

where ${n}_{t}^{a}$ denotes the number of individuals in age group $a$. Of note, aggregate human capital increases with the number of working-age individuals who live in the economy (that is, with a higher ${n}_{t}={\sum }_{a=15}^{R}{n}_{t}^{(a)}$), with individual human capital endowment (that is, with a higher ${h}_{t}^{(a)}$ for at least one $a$), and with labor supply (that is, with a higher ${{\mathcal{l}}}_{t}^{(a)}$ for at least one $a$).

We followed ref. ^50^ and constructed the average human capital of the cohort aged $a$ according to an exponential function of education and work experience:

$${h}_{t}^{\left(a\right)}=\exp \left[{\eta }_{1}\left(y{s}_{t}^{\left(a\right)}\right)+{\eta }_{2}\left(a-y{s}_{t}^{\left(a\right)}-5\right)+{\eta }_{3}{\left(a-y{s}_{t}^{\left(a\right)}-5\right)}^{2}\right],$$

where ${\eta }_{1}$ is the semi-elasticity of human capital with respect to average years of education as given by $y{s}_{t}^{\left(a\right)}$, and ${\eta }_{2}$ and ${\eta }_{3}$ are the semi-elasticities of human capital with respect to the experience of the workforce $\left(a-y{s}_{t}^{\left(a\right)}-5\right)$ and the experience of the workforce squared ${\left(a-y{s}_{t}^{\left(a\right)}-5\right)}^{2}$, respectively. Here we assumed a school entry age of 5 years throughout.

### Impact of diabetes mellitus on labor supply

Following refs. ^15,17,18^, the evolution of labor supply in the status quo scenario is given by

$${L}_{t}^{\left(a\right)}={{\mathcal{l}}}_{t}^{\left(a\right)}{n}_{t}^{\left(a\right)}\,\mathrm{with}\,{n}_{t}^{\left(a\right)}=\left[1-{\sigma }_{t-1}^{\left(a-1\right)}\right]{n}_{t-1}^{\left(a-1\right)},$$

where ${\sigma }_{t}^{\left(a\right)}$ is the overall mortality rate of age group $a$ in time $t$. Mortality and morbidity reduce effective labor supply.

Let ${\sigma }_{r,t}^{\left(a\right)}$ denote the mortality rate of people in age group $a$ due to diabetes mellitus, and let ${\sigma }_{-r,t}^{\left(a\right)}$ be the overall mortality rate due to the causes other than diabetes mellitus. Then we have

$$\left(1-{\sigma }_{t}^{\left(a\right)}\right)=(1-{\sigma }_{r,t}^{\left(a\right)})(1-{\sigma }_{-r,t}^{\left(a\right)}).$$

Mortality from diabetes mellitus reduces the labor supply by reducing the population ${n}_{t}^{\left(a\right)}$ (through ${\sigma }_{r,t}^{\left(a\right)}$). In the counterfactual case, in which diabetes mellitus is eliminated from time $t=0$ onward, the evolution of labor supply is defined similarly to the evolution of labor supply equation, but with a different overall mortality rate (${\sigma }_{-r,t}^{\left(a\right)}$ instead of ${\sigma }_{t}^{\left(a\right)}$). For simplicity, we assumed that the number of births is the same in both cases at each point in time $t$.

In the counterfactual scenario, the size of the cohort aged $a$ at time $t({\bar{n}}_{t}^{(a)})$ evolves according to

$${\bar{n}}_{t}^{(a)}=\left[1-{\sigma }_{-r,t-1}^{\left(a-1\right)}\right]{\bar{n}}_{t-1}^{(a-1)},\,{\bar{n}}_{0}^{(a)}={n}_{0}^{\left(a\right)},\,{\bar{n}}_{t}^{(0)}={n}_{t}^{\left(0\right)},$$

Following ref. ^15^, the loss of labor due to mortality accumulates over the years according to

$${\bar{n}}_{t}^{(a)}={n}_{t}^{\left(a\right)}/\mathop{\prod }\limits_{\tau =0}^{\min \left\{t,a\right\}-1}\left[1-{\sigma }_{r,t-1-\tau }^{\left(a-1-\tau \right)}\right].$$

The morbidity effect is captured by a reduction in the labor participation rate ${{\mathcal{l}}}_{t}^{\left(a\right)}$ because people with an illness typically reduce their labor supply, either by reducing their working hours or by leaving the workforce. Following ref. ^15^, the labor participation rate in the counterfactual scenario ${\bar{{\mathcal{l}}}}_{t}^{(a)}$ can be calculated as

$${\bar{{\mathcal{l}}}}_{t}^{(a)}\approx {{\mathcal{l}}}_{t}^{\left(a\right)}/\mathop{\prod }\limits_{\tau =0}^{\min \left\{t,a\right\}-1}\left[1-{p}^{\tau }{\sigma }_{r,t-1-\tau }^{\left(a-1-\tau \right)}{\xi }^{\,\left(a-1-\tau \right)}\right],$$

where ${\xi }^{\,\left(a\right)}$ measures the size of the morbidity effect relative to the relevant mortality rate, and where ${p}^{\tau }$ is the probability that a patient died from diabetes mellitus before time $t$.

Because the impact of morbidity is hard to estimate directly, we first defined

$${\xi }^{\,\left(a\right)}=\frac{\mathrm{loss}\,\mathrm{of}\,\mathrm{labor}\,\mathrm{due}\,\mathrm{to}\,\mathrm{morbidity}\,\mathrm{in}\,\mathrm{age}\,\mathrm{group}\,a}{\mathrm{loss}\,\mathrm{of}\,\mathrm{labor}\,\mathrm{due}\,\mathrm{to}\,\mathrm{mortality}\,\mathrm{in}\,\mathrm{age}\,\mathrm{group}\,a}.$$

Next, we assumed that the following holds in any given year for each age group $a$:

$${\xi }^{\,\left(a\right)}=\frac{{\mathrm{YLD}}^{\left(a\right)}}{{\mathrm{YLL}}^{\left(a\right)}},$$

where ${\mathrm{YLD}}^{\left(a\right)}$ represents the years lived with diabetes mellitus and ${\mathrm{YLL}}^{\left(a\right)}$ represents the years of life lost due to diabetes mellitus. Of note, ${\xi }^{\,\left(a\right)}$ can be calculated from the corresponding DALY data reported by the GBD study^58^.

In sum, as a result of the elimination of diabetes mellitus, the ‘counterfactual scenario’ is associated with an increase in labor supply as compared with the status quo scenario. We approximated the change in labor supply (at time $t$ for age group $a$) by

$$\Delta {L}_{t}^{\left(a\right)}\approx {{\mathcal{l}}}_{t}^{\left(a\right)}{n}_{t}^{\left(a\right)}\mathop{\sum }\limits_{\tau =0}^{\min \left\{t,a\right\}-1}{\sigma }_{r,t-1-\tau }^{\left(a-1-\tau \right)}\left[1+{p}^{\tau }{\xi }^{\,\left(a-1-\tau \right)}\right].$$

For the more general case of a partial reduction in the prevalence of diabetes mellitus by a factor $\rho$, we obtained the loss of labor for age group $a$ at time $t$ as

$$\Delta {L}_{t}^{\left(a\right)}\left(\rho \right)\approx {{\mathcal{l}}}_{t}^{\left(a\right)}{n}_{t}^{\left(a\right)}\mathop{\sum }\limits_{\tau =0}^{\min \left\{t,a\right\}-1}{\rho \sigma }_{r,t-1-\tau }^{\left(a-1-\tau \right)}\left[1+{p}^{\tau }{\xi }^{\,\left(a-1-\tau \right)}\right].$$

The details in ref. ^15^ showed the mathematical proof.

For the modeling of informal care labor, we simply subtract the labor loss associated with informal care (defined as a fraction of diabetes mellitus prevalence) from the effective labor supply.

### Impact of diabetes mellitus on physical capital accumulation

Diabetes mellitus also impedes the accumulation of physical capital because savings finance part of the treatment costs. Following refs. ^15,17^, physical capital accumulation in the counterfactual scenario can be written as

$${\bar{K}}_{t+1}={\bar{s}}_{t}{\bar{Y}}_{t}+\left(1-\delta \right){\bar{K}}_{t},$$

$${\bar{s}}_{t}{\bar{Y}}_{t}={\bar{I}}_{t}={\bar{Y}}_{t}-{\bar{C}}_{t}={{\rm{s}}}_{t}{\bar{Y}}_{t}+\chi{\rm{TC}}_{t},$$

where an overbar indicates the counterfactual scenario and where $\chi$ is the fraction of the treatment cost that is diverted to savings. The counterfactual saving rate is thus defined by

$${\bar{s}}_{t}=\frac{{s}_{t}{\bar{Y}}_{t}+\chi{\rm{TC}}_{t}}{{\bar{Y}}_{t}}.$$

For more details, see refs. ^15,17^.

Because diabetes mellitus is assumed to be eliminated in the counterfactual scenario, the resources that were devoted to its treatment can now be used for savings or consumption. Of note, this creates an income effect that, in reality, could affect the division of households’ income between savings and consumption. For tractability, we assumed that aggregate investment consists of two parts in the counterfactual scenario, which are as follows: a fixed share ${{\rm{s}}}_{t}$ of total output and an additional part from ${\rm{TC}}_{t}$ that would otherwise have been used to pay to treat diabetes mellitus:

$${\bar{I}}_{t}={{\rm{s}}}_{t}{\bar{Y}}_{t}+\chi{\rm{TC}}_{t},$$

Similarly, for the case of a partial reduction in diabetes mellitus prevalence by $\rho$, we have

$${\bar{I}}_{t}={{\rm{s}}}_{t}{\bar{Y}}_{t}+\rho \chi{\rm{TC}}_{t}.$$

The intuition is that if diabetes mellitus were partially eliminated, the treatment cost that is diverted to savings should be added back proportionally.

### Sensitivity analyses

We conducted several sensitivity analyses. First, we varied the mortality and morbidity rates. The baseline estimates were calculated with the mean mortality and morbidity data from GBD. In the sensitivity analyses, best-case and worst-case estimates were calculated based on the lower and upper bounds of GBD mortality and morbidity data. Table 1 presents the results of this sensitivity analysis in parentheses next to the baseline estimates. Second, we varied the discount rate. In the main analysis, we generated our estimates using a discount rate of 2%. We present estimates for each country by World Bank region and World Bank income group using discount rates of 0% in Supplementary Table 6 and 3% in Supplementary Table 7. Finally, we conducted sensitivity analyses by varying the weekly informal care hours from 0.285 to 8.3, with 4.0 as the median value (Supplementary Table 8).

### Reporting summary

Further information on research design is available in the Nature Portfolio Reporting Summary linked to this article.

## Online content

Any methods, additional references, Nature Portfolio reporting summaries, source data, extended data, supplementary information, acknowledgements, peer review information; details of author contributions and competing interests; and statements of data and code availability are available at 10.1038/s41591-025-04027-5.

## Supplementary information

Supplementary Information Supplementary Note, Figs. 1–6 and Tables 1–12.

Reporting Summary

## Source data

Source Data Fig. 1 Statistical source data.

Source Data Fig. 2 Statistical source data.

Source Data Fig. 3 Statistical source data.

## Data Availability

All data used in this study are publicly available from existing repositories and databases. Detailed descriptions of the data sources, access links and processing procedures are provided in the Methods. No new datasets were generated for this study. Source data underlying the figures are provided with the paper. Source data are provided with this paper.

## Code availability

All the code used for the descriptive tables and the analysis of the primary and safety endpoints is publicly available at https://github.com/caozhong14/HMM_diabetes.
