# Supplementary material for: The global macroeconomic burden of diabetes mellitus
Source: Nat Med. 2025 Dec 29;32(1):126–38. doi: 10.1038/s41591-025-04027-5 (PMC12823416; doi:10.1038/s41591-025-04027-5)
Supplement: Supplementary file 1 — Supplementary Note, Figs. 1–6 and Tables 1–12. [file 41591_2025_4027_MOESM1_ESM.pdf]

nature medicine

Supplementary information

https://doi.org/10.1038/s41591-025-04027-5

The global macroeconomic burden of diabetes mellitus

In the format provided by the authors and unedited

SI Appendix

In this supporting information (SI) appendix to the paper "The global macroeconomic cost of diabetes mellitus" we provide additional details related to our study, including the mathematical formulation of our model and detailed data sources.

SI Appendix 1
A: Global health burden of diabetes mellitus and additional economic burden due to COVID-19 1
B: Data description 4
C: Imputation 13
D: Sensitivity analyses 14
E: Contribution of treatment costs and informal labor costs 37
F: Strengths and limitations 39
Strengths 39
Limitations 39

A: Global health burden of diabetes mellitus and additional economic burden due to COVID-19

Figures S1–S3 show the health burden of diabetes mellitus. The numbers are based on the Global Burden of Disease Study 2021.[1,2]

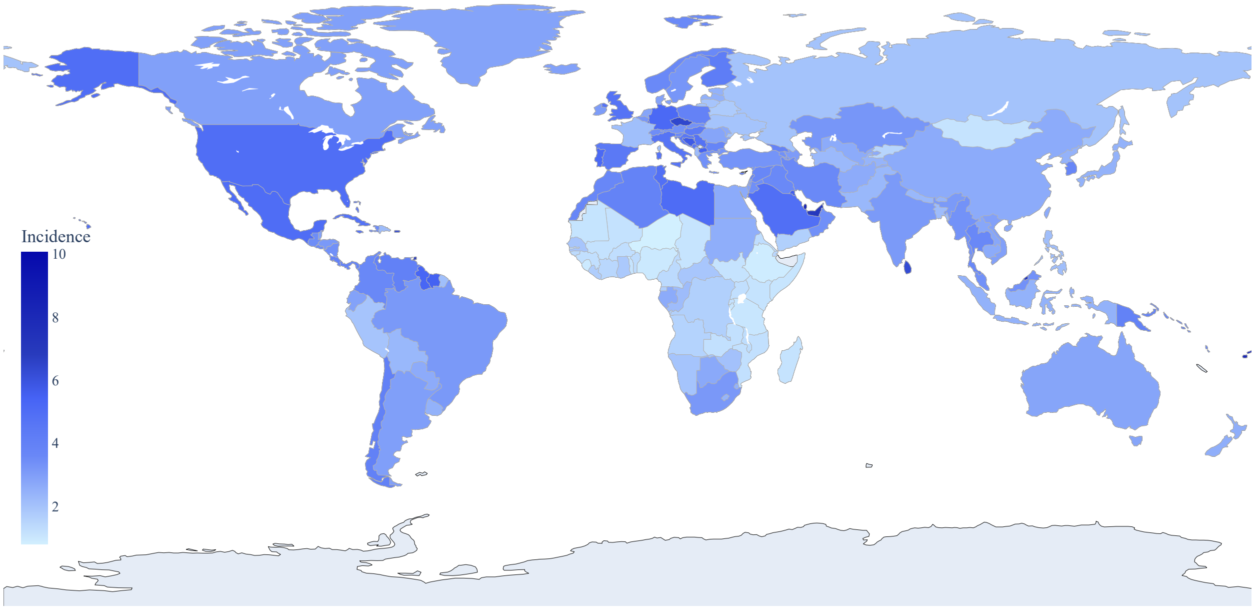

Figure S1. Diabetes mellitus incidence rate (per 1,000) in 2021

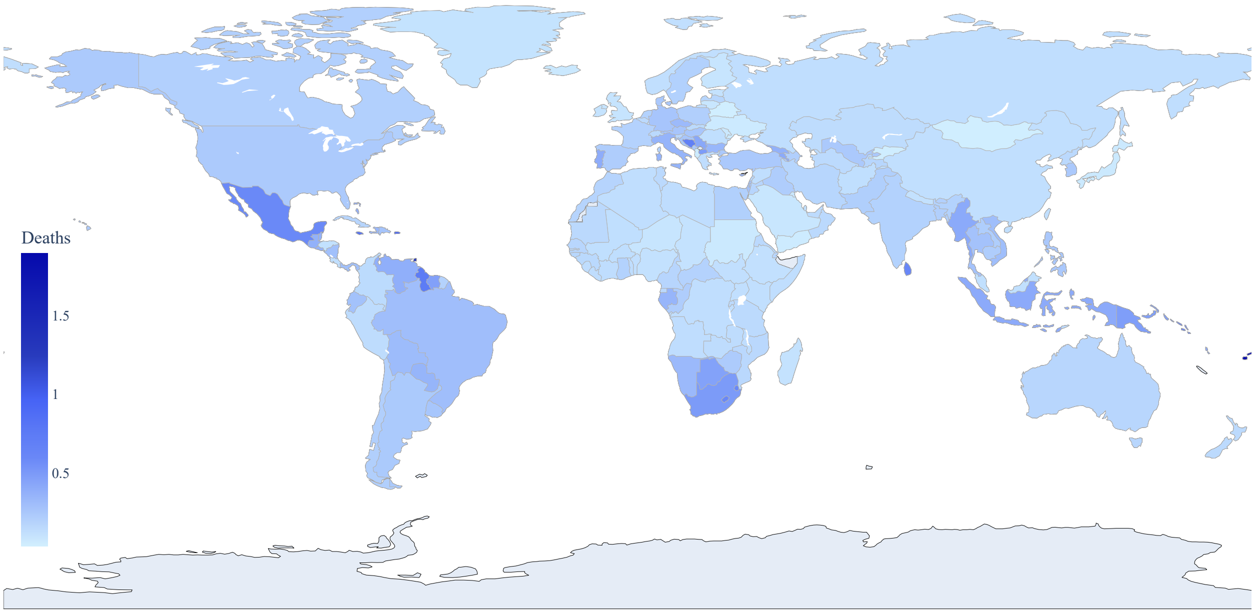

Figure S2. Diabetes mellitus mortality rate (per 1,000) in 2021

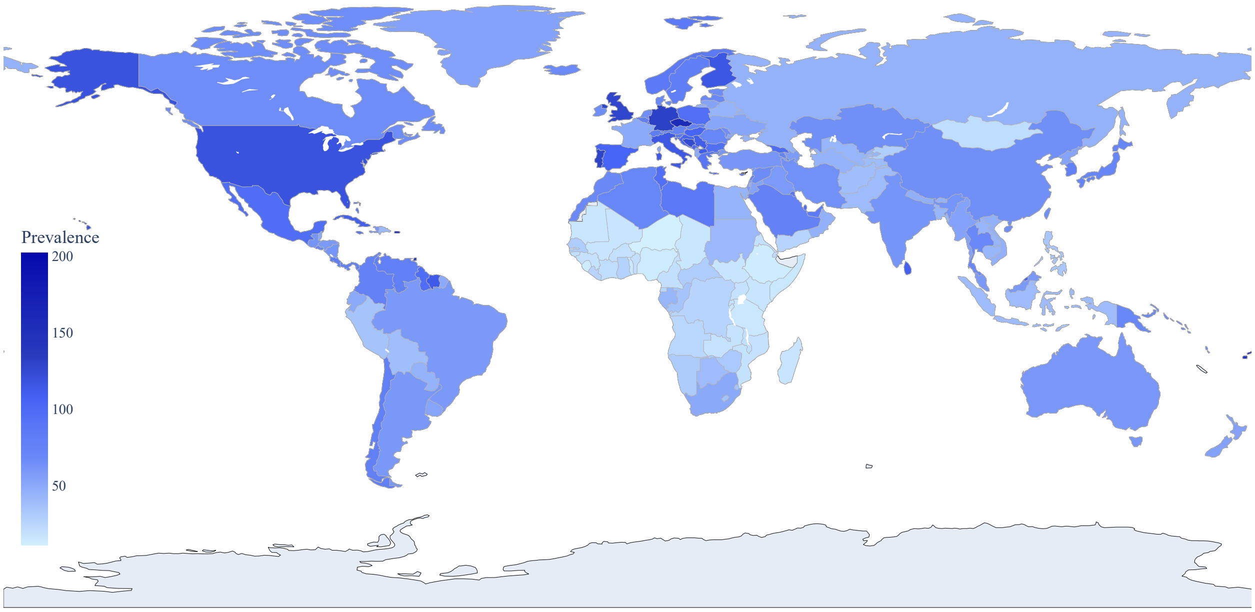

Figure S3. Diabetes mellitus prevalence rate (per 1,000) in 2021

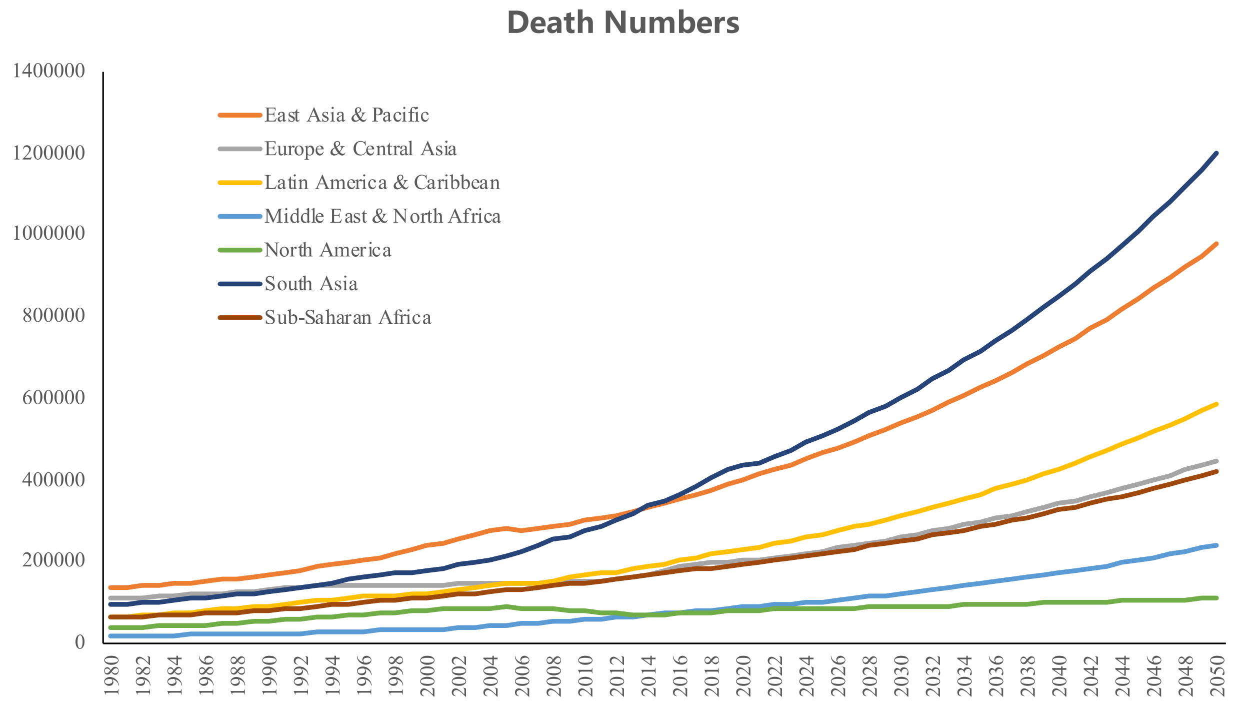

Figure S4. Number of deceased from diabetes mellitus from 1980 to 2050.

Figures S5–S6 show the macroeconomic burden of diabetes mellitus due to COVID-19. Due to a lack of data for some countries, we only calculated macroeconomic costs of diabetes mellitus due to COVID-19 for 134 countries, representing more than 90% of global population.

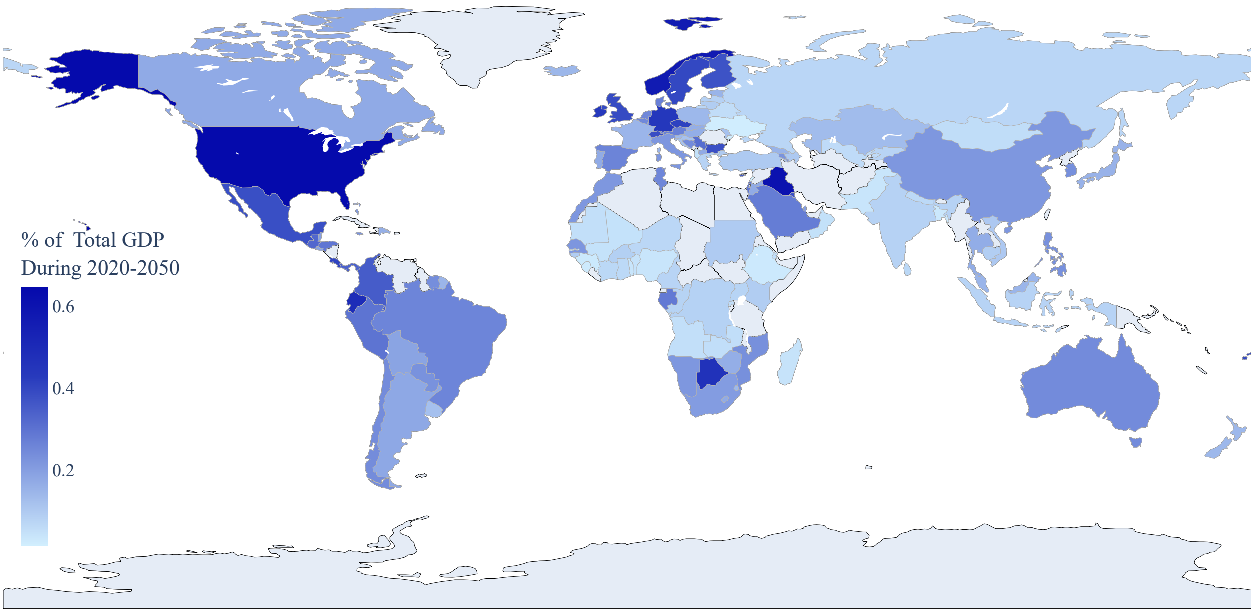

Figure S5. Macroeconomic cost of diabetes mellitus due to COVID-19 as a percentage of total GDP
White regions represent countries with insufficient data.

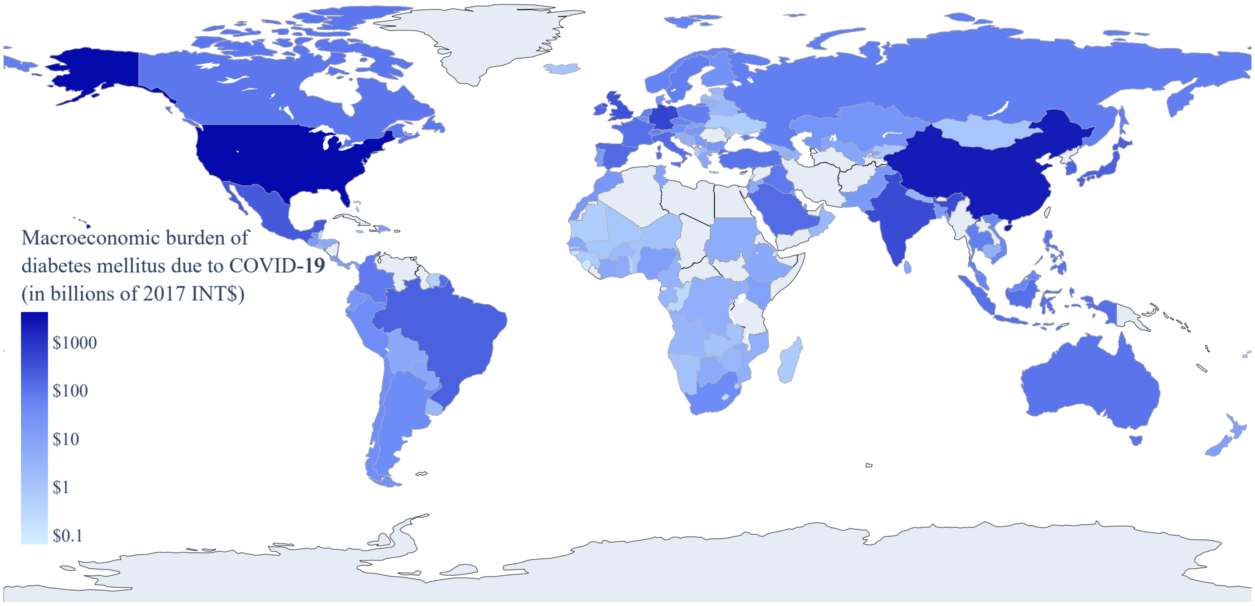

Figure S6. Macroeconomic cost of diabetes mellitus due to COVID-19 in billions of 2017 international dollars (2017 INT$)
White regions represent countries with insufficient data.

B: Data description

Education

Age-specific educational attainment data were obtained from the Barro-Lee Educational Attainment Database,[3] which provides educational attainment data by five-year age groups up to 2010. For 2010–2030, no age-specific data are available, but the database provides projections for the population aged 15–64. We approximated the age-specific estimates by assuming that educational attainment for each age group grows at the same rate. Because the Barro-Lee database presents data in five-year intervals, linear interpolation was adopted to extend the estimates for each year.

Mortality/morbidity

The mortality and morbidity (measured in years of life lost, YLLs, and years lost to disability, YLDs) of diabetes mellitus up to 2021 were obtained from the recently updated GBD estimates.[2,4] To extend the estimates beyond 2021, we assumed that the mortality rate of diabetes mellitus grows at the same rate as in 2010–2021 for each country. Morbidity estimates were obtained similarly.

GDP projection

GDP estimates (in 2017 INT$) up to 2020 were obtained from the World Bank database.[5] The GDP growth rates for 2021 to 2027 are from the International Monetary Fund's World Economic Outlook as of April 2020.[6] To extend the estimates to 2050, we assumed that growth beyond 2027 will be the same as in 2015–2019. The average GDP growth rate was 3.3% from 2010 to 2019 and is projected at 2.13% from 2020 to 2027.[6] For 17 countries without World Bank data, we used 2.1% as the average growth rate from 2020 to 2027, as estimated by the International Monetary Fund, and 3.3% as the average growth rate from 2028 to 2050; we then used the latest year of data from the Central Intelligence Agency as the base of projection to calculate total GDP from 2020 to 2050.[7]

Physical capital

For each country, the physical capital stock (in 2017 INT$) was obtained from the Penn World Table projections.[8] We used data from 2019, the last year for which physical capital data were available.

Labor
For each country, the labor participation rates by five-year age groups were obtained from the International Labour Organization database for 2010–2020.[9] For estimates beyond 2020, we first estimated the percentage growth rate of the labor participation rate in 2010–2020 using ordinary least squares. Then we assumed the percentage growth rate will remain the same as in 2010–2020 in estimating the labor participation rates for the five-year age groups from 2020 to 2050.

Population
For each country, the population by five-year age group was obtained from the population dynamics database built by the United Nations Department of Economic and Social Affairs.[10] For the 16 countries without five-year age group data, we used the total population from the Department of Economic and Social Affairs to calculate the per capita economic burden of diabetes mellitus.

Saving rate and health expenditure
We obtained country-specific saving rates and health expenditures from the World Bank database.[11] For the projection, we assumed that the saving rate would remain constant (at the average from 2010 to 2019) and that health expenditures (as a percentage of GDP) would grow at the same rate as in 2000–2019.

Informal caregiver
We obtained informal caregiver hours based on the estimate of caregiver weekly hours provided by Langa et al.,[12] which are as follows: 2.4–4.4 weekly hours of informal care attributable to diabetes mellitus without medication, 1.9–4.0 weekly hours of informal care attributable to diabetes mellitus with oral medication, and 4.0–8.3 weekly hours for diabetes mellitus with insulin. The International Labour Organization reported an average of 35.9 weekly hours per employed person.[13] Thus, for each diabetes patient, 0.05–0.23 units of working labor are lost due to informal caregiving.

Treatment costs
We used data from Dieleman et al. (2020) to calculate the treatment cost.[14] We calculated the per-case costs for the countries with data and extrapolated costs for countries without data, under the assumption that the per-case costs are proportional to per capita health expenditure, as in previous studies.[15-17] Specifically, we calculated the diabetes mellitus treatment cost per case for each country with data using GBD disease prevalence data and extrapolated costs to other countries using a scaling factor (defined as the ratio of health expenditure per capita between the country of interest and the United States). We then calculated the diabetes mellitus treatment cost per capita for each country by multiplying the treatment cost per case by the prevalence rate of diabetes mellitus. For years after 2020, we assumed that the treatment cost of diabetes mellitus would grow at the same rate as per capita health expenditures for each country. We also provided treatment costs of diabetes mellitus from the International Diabetes Federation (IDF) for comparison.[18,19] We double-checked the data we calculated based on Dieleman against the primary data from IDF. Please refer to **Table S1** for detailed results for each country. The treatment costs from IDF (the second-to-last column in **Table S1**) are 1.14-9.96 times the values we calculated and used in our work (the last column in **Table S1**). Thus, we may have underestimated the burden of diabetes mellitus.

Table S1. Treatment cost per capita (in 2017 INT$) for each available country by data source

Region	Country	For all diseases	For diabetes mellitus (IDF)	For diabetes mellitus (Dieleman)
**East Asia & Pacific**	Australia	4918.49578	359.2463534	113.5346189
**East Asia & Pacific**	Brunei Darussalam	1343.73412	97.81977882	50.89836755
**East Asia & Pacific**	Cambodia	293.145432	11.26394667	3.934447593
**East Asia & Pacific**	China	866.951791	75.88462962	21.46764881
**East Asia & Pacific**	Fiji	438.25497	77.30825513	22.67452105
**East Asia & Pacific**	Indonesia	332.469074	12.9037096	4.4701269
**East Asia & Pacific**	Japan	4445.0507	235.9481456	108.7070397

Region	Country	For all diseases	For diabetes mellitus (IDF)	For diabetes mellitus (Dieleman)
**East Asia & Pacific**	Kiribati	227.310408	48.02770298	7.852965716
**East Asia & Pacific**	Korea, Rep.	3620.79099	201.7166061	96.58499862
**East Asia & Pacific**	Lao PDR	202.963793	8.859612415	2.981271522
**East Asia & Pacific**	Malaysia	1035.7384	64.2586347	22.16361449
**East Asia & Pacific**	Marshall Islands	642.584361	232.7149613	34.41716894
**East Asia & Pacific**	Micronesia, Fed. Sts.	390.870213	101.3249749	13.6102096
**East Asia & Pacific**	Mongolia	440.441904	12.47506347	3.286244188
**East Asia & Pacific**	Myanmar	239.799084	9.098086418	4.416802702
**East Asia & Pacific**	Nauru	1284.24435	70.87735894	30.02637725
**East Asia & Pacific**	New Zealand	4457.15294	216.9681058	78.75187308
**East Asia & Pacific**	Palau	2344.27338	352.0375773	138.8243832
**East Asia & Pacific**	Papua New Guinea	93.4805052	11.6581416	2.293534118
**East Asia & Pacific**	Philippines	330.506718	15.1129093	3.630628062
**East Asia & Pacific**	Samoa	417.194707	48.83104108	12.95428716
**East Asia & Pacific**	Singapore	3772.10931	202.3468135	109.1138009
**East Asia & Pacific**	Solomon Islands	114.437937	16.8187281	2.732572119
**East Asia & Pacific**	Thailand	663.414876	45.08409191	13.27036719
**East Asia & Pacific**	Timor-Leste	295.54647	10.47386418	3.22577782
**East Asia & Pacific**	Tonga	319.033941	48.48993769	9.576192042
**East Asia & Pacific**	Tuvalu	1056.95632	177.9505417	40.357608
**East Asia & Pacific**	Vanuatu	96.5043391	21.76596782	2.451790591
**East Asia & Pacific**	Vietnam	438.021396	19.25695739	6.621052003
**Europe & Central Asia**	Albania	663.325998	33.55389769	10.80113537
**Europe & Central Asia**	Andorra	-	227.8827123	-
**Europe & Central Asia**	Armenia	1504.54483	87.90304574	40.35174105
**Europe & Central Asia**	Austria	5399.25195	496.3764109	143.4699313
**Europe & Central Asia**	Azerbaijan	568.473856	27.99707785	11.46971985
**Europe & Central Asia**	Belarus	1114.96972	45.23512902	17.77716901
**Europe & Central Asia**	Belgium	5264.80424	504.4568074	142.6243013
**Europe & Central Asia**	Bosnia and Herzegovina	1323.63279	140.4486679	56.68603284
**Europe & Central Asia**	Bulgaria	1608.59217	179.4448471	56.20978467
**Europe & Central Asia**	Croatia	1865.56897	128.2842099	68.35667538
**Europe & Central Asia**	Cyprus	2054.12505	237.342309	66.39452163
**Europe & Central Asia**	Czech Republic	3062.79223	341.5725341	163.0131027
**Europe & Central Asia**	Denmark	5669.05428	542.4486024	137.1963393
**Europe & Central Asia**	Estonia	2429.8468	120.9911615	58.68045918
**Europe & Central Asia**	Finland	4384.55934	637.759521	172.0781255

Region	Country	For all diseases	For diabetes mellitus (IDF)	For diabetes mellitus (Dieleman)
**Europe & Central Asia**	France	4886.66241	288.6463145	86.47758706
**Europe & Central Asia**	Georgia	868.468083	86.12581536	31.37100276
**Europe & Central Asia**	Germany	6024.41069	872.6465314	271.9142266
**Europe & Central Asia**	Greece	2185.32398	157.0954058	68.16509277
**Europe & Central Asia**	Hungary	1984.38428	155.7043309	72.26926675
**Europe & Central Asia**	Iceland	4818.93916	566.4893657	110.0864516
**Europe & Central Asia**	Ireland	6251.21352	519.7540173	130.1413077
**Europe & Central Asia**	Italy	3358.81086	376.9022836	127.7772446
**Europe & Central Asia**	Kazakhstan	706.148915	49.58312439	17.68897695
**Europe & Central Asia**	Kyrgyz Republic	216.788252	6.756060165	2.232390401
**Europe & Central Asia**	Latvia	1996.7185	91.2322573	49.70498671
**Europe & Central Asia**	Lithuania	2690.31817	71.85678961	51.78171994
**Europe & Central Asia**	Luxembourg	5986.2547	839.9206197	201.5602583
**Europe & Central Asia**	Moldova	509.117192	32.38582152	12.30543324
**Europe & Central Asia**	Monaco	-	314.4161943	-
**Europe & Central Asia**	Montenegro	1510.71921	-	53.28686064
**Europe & Central Asia**	Netherlands	5692.20706	425.6952404	129.4455155
**Europe & Central Asia**	North Macedonia	1126.64267	109.0866766	43.82781381
**Europe & Central Asia**	Norway	6724.317	944.6601789	179.6668537
**Europe & Central Asia**	Poland	2109.8615	95.45612259	70.92376405
**Europe & Central Asia**	Portugal	3087.7083	295.5630514	138.0586089
**Europe & Central Asia**	Romania	1665.42958	65.43030072	40.04097109
**Europe & Central Asia**	Russian Federation	1504.61794	79.20146905	24.16639078
**Europe & Central Asia**	San Marino	4007.53671	313.8595397	112.0011986
**Europe & Central Asia**	Serbia	1261.64986	158.6777016	49.17328036
**Europe & Central Asia**	Slovak Republic	2154.39595	116.840797	55.11706059
**Europe & Central Asia**	Slovenia	3218.01404	213.470029	93.69636794
**Europe & Central Asia**	Spain	3414.37372	320.1171016	122.0212272
**Europe & Central Asia**	Sweden	5828.65085	614.377021	165.8459057
**Europe & Central Asia**	Switzerland	7826.93798	1045.749485	227.1533203
**Europe & Central Asia**	Tajikistan	267.399983	6.777192712	3.706767374
**Europe & Central Asia**	Turkey	1217.61134	63.55617918	27.69259491
**Europe & Central Asia**	Turkmenistan	975.28169	75.32427384	15.24181919
**Europe & Central Asia**	Ukraine	854.440959	32.57068883	16.14435187
**Europe & Central Asia**	United Kingdom	4369.85418	752.1139625	200.8467427
**Europe & Central Asia**	Uzbekistan	420.849727	9.01977907	6.276438685
**Latin America & Caribbean**	Antigua and Barbuda	774.034283	103.2935677	26.63123454

Region	Country	For all diseases	For diabetes mellitus (IDF)	For diabetes mellitus (Dieleman)
Latin America & Caribbean	Argentina	1924.20174	85.34197601	38.39220123
Latin America & Caribbean	Bahamas, The	1827.93974	219.3194256	54.43168333
Latin America & Caribbean	Barbados	805.331113	132.1065313	32.90695642
Latin America & Caribbean	Belize	373.014559	42.40041583	6.490587395
Latin America & Caribbean	Bolivia	554.990792	34.90393712	7.521095661
Latin America & Caribbean	Brazil	1353.31655	161.2434852	28.71097554
Latin America & Caribbean	Chile	2208.8789	124.7822889	57.98105171
Latin America & Caribbean	Colombia	1055.29175	134.6356997	27.1339087
Latin America & Caribbean	Costa Rica	1463.33482	235.4786222	39.7834682
Latin America & Caribbean	Cuba	-	342.3449703	-
Latin America & Caribbean	Dominica	568.147949	173.3745924	25.92122284
Latin America & Caribbean	Dominican Republic	1032.75798	61.75528506	14.53694093
Latin America & Caribbean	Ecuador	835.911435	114.2459929	14.66437497
Latin America & Caribbean	El Salvador	567.164586	76.25365203	13.85433777
Latin America & Caribbean	Grenada	714.943015	113.3995973	26.22286801
Latin America & Caribbean	Guatemala	490.124589	55.17983444	10.65806986
Latin America & Caribbean	Guyana	933.748534	67.9881698	32.46473848
Latin America & Caribbean	Haiti	143.144917	14.77161773	3.020670715
Latin America & Caribbean	Honduras	378.541597	44.05582273	7.66997613
Latin America & Caribbean	Jamaica	539.646338	81.74004513	16.99299703
Latin America & Caribbean	Mexico	972.523115	136.748444	33.22650078
Latin America & Caribbean	Nicaragua	455.089785	35.56292558	9.488955235
Latin America & Caribbean	Panama	1930.26459	114.3037096	52.1337717
Latin America & Caribbean	Paraguay	908.036869	60.09527534	13.75157828
Latin America & Caribbean	Peru	590.99191	47.35766716	7.064403068
Latin America & Caribbean	St. Kitts and Nevis	1326.50307	106.5593074	44.63961352
Latin America & Caribbean	St. Lucia	518.432133	127.4064111	25.53703172
Latin America & Caribbean	St. Vincent and the Grenadines	576.347255	111.1466281	24.11704037
Latin America & Caribbean	Suriname	1546.8016	144.7659882	63.03639335

Region	Country	For all diseases	For diabetes mellitus (IDF)	For diabetes mellitus (Dieleman)
**Latin America & Caribbean**	Trinidad and Tobago	1717.33258	177.2818855	85.38651298
**Latin America & Caribbean**	Uruguay	2012.51002	89.4460345	33.16316025
**Middle East & North Africa**	Algeria	705.768685	60.96994851	16.70049878
**Middle East & North Africa**	Bahrain	1700.4139	177.8370818	70.27421189
**Middle East & North Africa**	Djibouti	98.565125	6.475542516	0.763039402
**Middle East & North Africa**	Egypt, Arab Rep.	566.307335	-	8.639179665
**Middle East & North Africa**	Iran, Islamic Rep.	878.484627	86.45308993	18.22465766
**Middle East & North Africa**	Iraq	406.502196	49.81831216	8.196378332
**Middle East & North Africa**	Israel	3109.1667	284.4572204	66.38876884
**Middle East & North Africa**	Jordan	734.378237	52.71785652	13.08868531
**Middle East & North Africa**	Kuwait	2562.47366	165.3571263	73.13131585
**Middle East & North Africa**	Lebanon	978.823197	157.0592717	27.11081721
**Middle East & North Africa**	Libya	656.933521	60.05789236	18.5636619
**Middle East & North Africa**	Malta	3870.19943	334.8122252	158.0787766
**Middle East & North Africa**	Morocco	382.634928	32.41402716	8.719907729
**Middle East & North Africa**	Oman	1228.24384	38.90080324	19.97770112
**Middle East & North Africa**	Qatar	2482.48937	202.4122884	89.16228263
**Middle East & North Africa**	Saudi Arabia	2592.29283	132.0763925	66.40978366
**Middle East & North Africa**	Syrian Arab Republic	8.099298964	-	
**Middle East & North Africa**	Tunisia	729.336457	59.07781318	23.24873632
**Middle East & North Africa**	United Arab Emirates	2811.53368	177.2949164	68.54159002
**Middle East & North Africa**	Yemen, Rep.	-	3.151144364	-
**North America**	Canada	5117.41987	317.9890891	113.0527783
**North America**	United States	10061.9304	1395.581511	413.5447446
**South Asia**	Afghanistan	260.945554	5.556707448	3.547176786
**South Asia**	Bangladesh	120.986152	3.271177483	1.73234243
**South Asia**	Bhutan	373.112787	8.7205936	5.525380405
**South Asia**	India	183.146184	7.023956793	3.532363788
**South Asia**	Maldives	1026.45816	68.88984938	12.36235387
**South Asia**	Nepal	173.140166	4.7449698	2.640702673
**South Asia**	Pakistan	153.798265	3.184554515	2.15591296

Region	Country	For all diseases	For diabetes mellitus (IDF)	For diabetes mellitus (Dieleman)
**South Asia**	Sri Lanka	519.399052	21.85742181	19.08985791
**Sub-Saharan Africa**	Angola	154.795288	12.91909908	1.399659186
**Sub-Saharan Africa**	Benin	79.3709511	3.61232744	0.566848945
**Sub-Saharan Africa**	Botswana	889.819877	85.73437253	12.46262643
**Sub-Saharan Africa**	Burkina Faso	121.52707	3.693137546	0.842138889
**Sub-Saharan Africa**	Burundi	58.7985077	1.659231304	0.345001208
**Sub-Saharan Africa**	Cabo Verde	302.251047	39.29453997	4.542018476
**Sub-Saharan Africa**	Cameroon	130.762381	5.610585391	0.90446753
**Sub-Saharan Africa**	Central African Republic	74.4074021	8.102100738	0.822007968
**Sub-Saharan Africa**	Chad	64.8021392	2.195488509	0.394349093
**Sub-Saharan Africa**	Comoros	151.154171	4.694027877	1.207756808
**Sub-Saharan Africa**	Congo, Dem. Rep.	38.3515812	2.483733881	0.35818835
**Sub-Saharan Africa**	Congo, Rep.	71.5400388	8.847086691	0.888448821
**Sub-Saharan Africa**	Cote d'Ivoire	165.001469	8.206881109	1.222534645
**Sub-Saharan Africa**	Equatorial Guinea	544.804576	23.77465296	4.963997865
**Sub-Saharan Africa**	Eritrea	-	2.149692347	-
**Sub-Saharan Africa**	Eswatini	576.398158	40.05829981	7.367190189
**Sub-Saharan Africa**	Ethiopia	73.5939439	1.384073649	0.326976699
**Sub-Saharan Africa**	Gabon	396.452828	49.31884567	6.12694679
**Sub-Saharan Africa**	Gambia, The	80.1583144	2.555607902	0.545255147
**Sub-Saharan Africa**	Ghana	190.253994	7.786183931	1.946957779
**Sub-Saharan Africa**	Guinea	106.074267	3.41773153	0.712120167
**Sub-Saharan Africa**	Guinea-Bissau	155.469717	5.502066279	1.112122908
**Sub-Saharan Africa**	Kenya	198.93586	7.71991089	1.183783157
**Sub-Saharan Africa**	Lesotho	271.054477	17.15867805	3.589233871
**Sub-Saharan Africa**	Liberia	121.528344	6.074216638	1.162960208
**Sub-Saharan Africa**	Madagascar	53.6247726	1.696412517	0.325352126
**Sub-Saharan Africa**	Malawi	118.039175	3.069192539	0.862618168
**Sub-Saharan Africa**	Mali	84.5824327	3.257803101	0.524945103
**Sub-Saharan Africa**	Mauritania	167.122295	4.679113135	0.911760817
**Sub-Saharan Africa**	Mauritius	1252.5753	87.65718455	64.29747923
**Sub-Saharan Africa**	Mozambique	102.125224	4.197719832	0.686779918
**Sub-Saharan Africa**	Namibia	742.669052	65.93999107	8.303462091
**Sub-Saharan Africa**	Niger	68.8719267	1.555359388	0.257915925
**Sub-Saharan Africa**	Nigeria	146.976122	6.856870359	0.711019713
**Sub-Saharan Africa**	Rwanda	135.458148	4.164900139	0.856258744
**Sub-Saharan Africa**	Sao Tome and Principe	214.344936	17.61373253	1.952139097

Region	Country	For all diseases	For diabetes mellitus (IDF)	For diabetes mellitus (Dieleman)
**Sub-Saharan Africa**	Senegal	138.655416	9.24246639	1.579701129
**Sub-Saharan Africa**	Seychelles	1274.40936	101.6782838	45.60317619
**Sub-Saharan Africa**	Sierra Leone	146.424266	5.706264122	0.690411887
**Sub-Saharan Africa**	Somalia	-	0.321479285	-
**Sub-Saharan Africa**	South Africa	1167.16985	85.9094155	21.21430508
**Sub-Saharan Africa**	Sudan	185.229852	6.421836377	2.638269528
**Sub-Saharan Africa**	Tanzania	95.7400264	2.42183109	0.594548284
**Sub-Saharan Africa**	Togo	126.568701	3.252270266	0.681505616
**Sub-Saharan Africa**	Uganda	83.3962497	4.295443104	0.578222421
**Sub-Saharan Africa**	Zambia	167.528375	6.03121167	1.065681368
**Sub-Saharan Africa**	Zimbabwe	251.458701	26.14384662	2.956375204
**Others**	Cook Island	-	81.71025457	-
**Others**	Niue	-	370.6837199	-

Other parameter values and data sources

Table S2 shows other parameter values and data sources in the model, where definitions for parameters are consistent with Bloom et al. (2020)[20] and Chen et al. (2019).[17,21]

Table S2. Parameter values and data sources

Parameter	Definition	Value	Source
α	Capital share	Country specific	Penn World Table[8]
δ	Depreciation rate	0.05	Grossmann et al. (2013)[22]
η_1	Mincer elasticity of education	0.091	Psacharopoulos and Patrinos (2018)[23]
η_2	First-degree Mincer elasticity of experience	0.1301	Heckman et al. (2006)[24]
η_3	Second-degree Mincer elasticity of experience	−0.0023	Heckman et al. (2006)[24]
χ_i	Fraction of treatment cost financed out of saving	Set as saving rate	World Bank (2020)[11]

We lacked some data for 60 countries as described in **Table S3**, and we additionally needed to estimate data for Romania because its projection data are volatile and unreliable.

Table S3. Missing data for 60 countries. The columns represent region, country code, World Bank country, GDP, treatment cost, education, household expenditures per capita, labor participation rate, physical capital, population, and saving rate, respectively. Each "X" indicates missing data.

Region	Code	World Bank Country	GDP	TC	EDU	HE	LAB	CAP	POP	SAV
**East Asia & Pacific**	ASM	American Samoa	X			X	X	X	X	X
	PRK	Korea, Dem. People's Rep.	X		X	X		X		X
	GUM	Guam	X			X		X		X
	KIR	Kiribati					X	X		
	MHL	Marshall Islands					X	X	X	
	FSM	Micronesia, Fed. Sts.					X	X		X

Region	Code	World Bank Country	GDP	TC	EDU	HE	LAB	CAP	POP	SAV
	MMR	Myanmar						X		
	NRU	Nauru					X	X	X	X
	MNP	Northern Mariana Islands	X			X	X	X	X	X
	PLW	Palau					X	X	X	X
	PNG	Papua New Guinea						X		X
	WSM	Samoa						X		X
	SLB	Solomon Islands						X		
	TWN	Taiwan (Province of China)	X			X				X
	TLS	Timor-Leste						X		
	TON	Tonga						X		
	TUV	Tuvalu					X	X	X	X
	VUT	Vanuatu						X		
**Europe & Central Asia**	AND	Andorra	X			X	X	X	X	X
	ROU	Romania				X				
	GRL	Greenland	X			X	X	X	X	X
	MCO	Monaco	X			X	X	X	X	X
	SMR	San Marino					X	X	X	X
	TKM	Turkmenistan								X
**Latin America & Caribbean**	ATG	Antigua and Barbuda					X			
	CUB	Cuba	X			X		X		X
	DMA	Dominica					X		X	
	GRD	Grenada					X			X
	GUY	Guyana						X		X
	HTI	Haiti						X		
	NIC	Nicaragua						X		
	PRI	Puerto Rico				X		X		X
	KNA	St. Kitts and Nevis					X		X	X
	LCA	St. Lucia								X
	VCT	St. Vincent and the Grenadines								X
	TTO	Trinidad and Tobago								X
	VIR	Virgin Islands (U.S.)	X			X		X		X
	VEN	Venezuela, RB	X			X				
**Middle East & North Africa**	DZA	Algeria						X		
	IRN	Iran, Islamic Rep.								X
	LBY	Libya						X		X
	SYR	Syrian Arab Republic	X			X				
	ARE	United Arab Emirates						X		X
	YEM	Yemen, Rep.	X			X				X
**North America**	BMU	Bermuda	X			X	X		X	

Region	Code	World Bank Country	GDP	TC	EDU	HE	LAB	CAP	POP	SAV
**South Asia**	AFG	Afghanistan						X		X
**Sub-Saharan Africa**	CAF	Central African Republic								X
	TCD	Chad								X
	GNQ	Equatorial Guinea								X
	ERI	Eritrea	X			X		X		X
	LBR	Liberia								X
	MWI	Malawi								X
	STP	Sao Tome and Principe								X
	SYC	Seychelles			X		X	X		
	SOM	Somalia				X		X		X
	SSD	South Sudan	X			X		X		
**Others**	COK	Cook Islands	X		X	X	X	X	X	X
	NIU	Niue	X		X	X	X	X	X	X
	PSE	Palestine	X		X	X		X		
	TKL	Tokelau	X		X	X	X	X	X	X

C: Imputation

For the 60 countries with incomplete data (mostly on education, physical capital, and saving rate) but reliable data for GDP and the prevalence of diabetes mellitus, we used a linear projection to approximate the economic burden of diabetes mellitus. **Table S4** shows the regression results for the different parameters. Column 1 represents the discount rate, Column 2 represents informal care hours, and Column 3 represents which GBD mortality or morbidity values were used in the approximation. We used the percentage of economic loss in total GDP from 2020 to 2050 as the dependent variable and the average DALYs rate of diabetes mellitus in 2020-2050 as the independent variable, based on the results from the 144 countries with complete data.

Table S4. Estimating the relationship between percentage of economic loss and DALYs rate

Discount	Informal care hours	Mortality/ morbidity	Coefficient of constant (p-value)	Coefficient of DALYs rate (p-value)	R-square
0	0	middle	0.0006195059843903812 (1.338e-09)	1.2780502994683395e-06 (3.121e-38)	0.69295864
0	0	upper	0.000892419096600103 (3.632e-11)	1.1728177936128536e-06 (1.210e-41)	0.72503227
0	0	lower	0.00042194622379637095 (2.982e-07)	1.479447034885139e-06 (1.675e-32)	0.63050197
0.02	0	middle	0.0005699566504427052 (9.849e-10)	1.1793776793959192e-06 (9.334e-39)	0.69811948
0.02	0	upper	0.0008214098107127205 (2.572e-11)	1.0808197111856873e-06 (4.240e-42)	0.72905303
0.02	0	lower	0.00038833837213058384 (2.221e-07)	1.365184760324695e-06 (4.562e-33)	0.63718129
0.03	0	middle	0.000543818018308231 (8.503e-10)	1.128351437878248e-06 (4.616e-39)	0.70109012
0.03	0	upper	0.0007840769633558002 (2.169e-11)	1.0332552026757346e-06 (2.304e-42)	0.73136451
0.03	0	lower	0.0003705696665456147 (1.918e-07)	1.3060715531659218e-06 (2.134e-33)	0.64102749
0.02	middle	middle	0.005767827201045445 (1.577e-09)	7.925703477827829e-06 (5.407e-23)	0.4978459
0.02	upper	middle	0.011376291528299129 (3.267e-09)	1.509960376987519e-05 (2.818e-21)	0.4693043
0.02	lower	middle	0.003036441884207883 (3.569e-10)	4.392979070327353e-06 (4.508e-26)	0.54528149

We then imputed the percentage of the economic loss in total GDP using the coefficients from the regression and calculated the economic loss for the 60 countries with incomplete data.

D: Sensitivity analyses

We provided the macroeconomic burden of diabetes due to COVID-19 and performed two types of sensitivity analyses. The main paper shows the results with a discount rate of 2% without informal care in the baseline scenario. For sensitivity analyses, we varied the discount rates, and we varied the informal care hours.

1. **Table S5** shows the additional macroeconomic burden of diabetes mellitus due to COVID-19.
2. **Table S6** and **Table S7** show the results with varying discount rates of either 0% or 3% without informal care.
3. **Table S8** shows the results with varying informal care weekly hours with 4.0 as the baseline (lower bound of 0.283 and upper bound of 8.3), using a discount rate of 2%.

Table S5. Total macroeconomic cost, per capita economic cost, and economic cost as a percentage of GDP in 2020–2050 attributable to diabetes mellitus due to COVID-19, by country and World Bank region in 2017 INT$

Region	World Bank country	Economic cost in millions of 2017 INT$	Percentage of total GDP in 2020–2050	Per capita loss in 2017 INT$
East Asia & Pacific	Australia	99213(97603-101892)	0.24(0.24-0.25)	3384(3329-3476)
	Cambodia	3715(3471-4222)	0.09(0.09-0.10)	190(177-216)
	China	2137589(2136657-2141195)	0.22(0.22-0.22)	1477(1477-1480)
	Fiji	1277(1088-1651)	0.39(0.34-0.51)	1286(1097-1663)
	Indonesia	117529(96188-156596)	0.08(0.07-0.11)	383(313-510)
	Japan	200179(196437-207126)	0.16(0.15-0.16)	1714(1682-1773)
	Korea, Rep.	172130(170045-176494)	0.23(0.23-0.24)	3437(3396-3525)
	Malaysia	54661(52632-58521)	0.15(0.14-0.16)	1471(1417-1575)
	Mongolia	944(857-1175)	0.06(0.05-0.07)	242(220-302)
	New Zealand	10825(10526-11297)	0.14(0.14-0.15)	2051(1994-2140)
	Philippines	108270(104068-116235)	0.23(0.22-0.25)	840(807-902)
	Singapore	30092(29412-31350)	0.16(0.16-0.17)	4784(4676-4984)
	Thailand	69682(67954-72813)	0.17(0.16-0.18)	1007(982-1053)
	Vietnam	52790(48420-61768)	0.10(0.10-0.12)	501(460-587)
Europe & Central Asia	Albania	663(566-841)	0.05(0.04-0.06)	246(210-312)
	Armenia	3434(3238-3783)	0.23(0.21-0.25)	1174(1107-1294)
	Austria	35985(35406-37020)	0.27(0.26-0.28)	3928(3865-4041)
	Azerbaijan	3906(3225-5580)	0.10(0.09-0.15)	361(298-516)
	Belarus	2377(2141-2877)	0.06(0.05-0.07)	262(236-317)
	Belgium	40056(38990-41930)	0.25(0.24-0.26)	3343(3254-3499)
	Bosnia and Herzegovina	4047(3759-4729)	0.24(0.23-0.29)	1343(1247-1569)
	Bulgaria	20756(19901-22521)	0.38(0.37-0.42)	3374(3235-3661)
	Croatia	4566(4206-5248)	0.12(0.11-0.14)	1218(1122-1400)
	Cyprus	4961(4923-5032)	0.34(0.34-0.35)	3832(3803-3887)
	Czech Republic	60393(57825-66116)	0.41(0.39-0.45)	5660(5419-6196)
	Denmark	27387(27021-28046)	0.27(0.27-0.28)	4521(4461-4630)
	Estonia	2667(2548-2893)	0.15(0.15-0.16)	2137(2042-2318)
	Finland	27877(27344-28854)	0.37(0.37-0.39)	5022(4926-5198)
	France	117977(115572-122043)	0.15(0.15-0.15)	1762(1727-1823)
	Georgia	3366(3137-3852)	0.15(0.14-0.18)	894(833-1023)
	Germany	537918(528247-554250)	0.45(0.44-0.46)	6527(6410-6725)
	Greece	6433(6173-6953)	0.08(0.08-0.09)	662(635-715)
	Hungary	19290(17778-22393)	0.17(0.15-0.19)	2118(1952-2459)

Region	World Bank country	Economic cost in millions of 2017 INT$	Percentage of total GDP in 2020–2050	Per capita loss in 2017 INT$
	Iceland	1007(987-1050)	0.14(0.14-0.15)	2768(2713-2886)
	Ireland	170560(168974-173680)	0.44(0.44-0.45)	31854(31558-32437)
	Italy	131372(128462-135915)	0.22(0.21-0.23)	2268(2218-2346)
	Kazakhstan	22071(20550-25517)	0.13(0.12-0.15)	1027(956-1187)
	Kyrgyz Republic	898(763-1256)	0.08(0.07-0.11)	114(97-160)
	Latvia	1671(1493-2059)	0.09(0.08-0.10)	1007(899-1240)
	Lithuania	3574(3292-4085)	0.10(0.09-0.11)	1493(1375-1707)
	Luxembourg	4929(4814-5142)	0.21(0.20-0.22)	6893(6731-7190)
	Moldova	1471(1374-1694)	0.12(0.11-0.14)	393(367-452)
	Netherlands	72639(71334-74752)	0.25(0.25-0.26)	4182(4107-4304)
	North Macedonia	1916(1676-2391)	0.17(0.15-0.22)	959(839-1197)
	Norway	57533(56973-58742)	0.57(0.56-0.58)	9501(9409-9701)
	Poland	69819(64469-79699)	0.14(0.13-0.16)	1941(1793-2216)
	Portugal	17235(16702-18273)	0.17(0.17-0.18)	1775(1720-1882)
	Russian Federation	67688(60706-80207)	0.07(0.07-0.09)	480(430-568)
	Serbia	15105(14319-16707)	0.31(0.30-0.35)	1900(1801-2101)
	Slovak Republic	9598(8924-11110)	0.17(0.15-0.19)	1816(1688-2102)
	Slovenia	4020(3859-4321)	0.14(0.13-0.15)	1985(1905-2134)
	Spain	138722(135856-143857)	0.26(0.26-0.27)	3043(2980-3155)
	Sweden	67601(66966-68705)	0.40(0.39-0.40)	6260(6202-6363)
	Switzerland	66649(65735-68266)	0.38(0.38-0.39)	7143(7045-7316)
	Turkey	102600(97711-112552)	0.10(0.10-0.11)	1122(1069-1231)
	Ukraine	589(264-1121)	0.02(0.01-0.04)	15(7-28)
	United Kingdom	326180(319892-337024)	0.39(0.38-0.40)	4571(4483-4723)
	Uzbekistan	8028(5838-12038)	0.06(0.04-0.09)	207(150-310)
**Latin America & Caribbean**	Argentina	41063(39352-44221)	0.18(0.17-0.19)	813(779-876)
	Bahamas, The	676(599-919)	0.21(0.18-0.28)	1552(1374-2108)
	Barbados	71(47-123)	0.07(0.05-0.13)	247(164-428)
	Belize	133(112-189)	0.20(0.16-0.28)	269(226-382)
	Bolivia	6325(5711-7823)	0.19(0.17-0.23)	455(411-563)
	Brazil	187951(183348-194835)	0.26(0.25-0.27)	836(815-866)
	Chile	33091(32410-34285)	0.24(0.24-0.25)	1671(1637-1732)
	Colombia	83187(81192-87025)	0.35(0.34-0.37)	1538(1501-1609)
	Costa Rica	15093(14646-16009)	0.41(0.40-0.43)	2724(2644-2890)
	Dominican Republic	15625(14175-19311)	0.16(0.15-0.20)	1300(1179-1607)
	Ecuador	23790(23195-25164)	0.49(0.48-0.52)	1149(1120-1215)
	El Salvador	3079(2833-3540)	0.19(0.17-0.22)	452(416-520)
	Guatemala	17646(16756-19388)	0.33(0.31-0.36)	779(740-856)
	Honduras	5708(5430-6431)	0.29(0.28-0.33)	474(451-534)
	Jamaica	1536(1376-1925)	0.22(0.20-0.28)	508(455-637)
	Mexico	252239(242179-267789)	0.38(0.37-0.41)	1747(1677-1854)
	Panama	15415(14926-16392)	0.34(0.33-0.36)	2989(2894-3178)
	Paraguay	7096(6685-8004)	0.23(0.22-0.26)	862(812-972)
	Peru	37932(37115-39724)	0.30(0.29-0.31)	1022(1000-1070)
	Suriname	472(430-568)	0.24(0.22-0.29)	733(668-881)
	Uruguay	2578(2503-2734)	0.12(0.12-0.13)	719(698-762)
**Middle East & North Africa**	Bahrain	7665(7470-8041)	0.33(0.32-0.35)	3687(3593-3868)
	Djibouti	168(127-303)	0.05(0.04-0.10)	145(109-261)
	Iraq	85339(83787-88958)	0.61(0.60-0.63)	1539(1511-1604)
	Israel	41778(41055-42980)	0.29(0.29-0.30)	3917(3849-4029)
	Jordan	5465(5241-5939)	0.17(0.17-0.19)	481(461-523)
	Kuwait	18628(18168-19443)	0.38(0.37-0.39)	3787(3694-3953)

Region	World Bank country	Economic cost in millions of 2017 INT$	Percentage of total GDP in 2020–2050	Per capita loss in 2017 INT$
	Lebanon	65(29-135)	0.01(0.01-0.03)	10(5-21)
	Malta	4408(4344-4539)	0.41(0.40-0.42)	9981(9838-10278)
	Morocco	19557(18841-21138)	0.22(0.21-0.24)	464(447-501)
	Oman	2330(1976-3086)	0.05(0.04-0.07)	380(322-503)
	Qatar	14458(13684-16692)	0.20(0.19-0.23)	4194(3970-4842)
	Saudi Arabia	132341(129045-139986)	0.28(0.27-0.29)	3262(3181-3450)
	Tunisia	8539(8222-9215)	0.26(0.25-0.28)	657(633-709)
**North America**	Canada	90057(88296-93087)	0.17(0.17-0.18)	2143(2101-2215)
	United States	4014424(3945564-4118734)	0.65(0.63-0.66)	11236(11043-11528)
**South Asia**	Bangladesh	17695(13582-29622)	0.03(0.02-0.05)	97(75-162)
	India	416233(373362-498896)	0.08(0.07-0.10)	271(243-325)
	Maldives	584(573-614)	0.18(0.17-0.18)	1069(1049-1123)
	Nepal	3574(3126-4492)	0.07(0.06-0.08)	106(93-134)
	Pakistan	17091(12060-26847)	0.04(0.03-0.06)	61(43-95)
	Sri Lanka	6745(5062-10037)	0.07(0.05-0.10)	307(230-457)
**Sub-Saharan Africa**	Angola	2644(2023-4165)	0.05(0.04-0.08)	50(38-78)
	Benin	962(807-1454)	0.05(0.04-0.07)	54(45-81)
	Botswana	5781(5647-6169)	0.47(0.46-0.50)	1953(1907-2084)
	Burkina Faso	2089(1856-2761)	0.09(0.08-0.12)	66(59-88)
	Cabo Verde	277(263-323)	0.22(0.21-0.26)	441(419-514)
	Cameroon	3258(2768-4376)	0.08(0.07-0.10)	86(73-115)
	Congo, Dem. Rep.	4105(3333-6118)	0.08(0.07-0.13)	30(24-44)
	Congo, Rep.	285(164-643)	0.08(0.05-0.18)	36(21-81)
	Côte d'Ivoire	6218(5777-7238)	0.07(0.07-0.08)	163(151-190)
	Eswatini	413(346-572)	0.14(0.12-0.19)	293(245-405)
	Ethiopia	6342(5188-8719)	0.03(0.02-0.04)	40(32-54)
	Gabon	2931(2780-3255)	0.29(0.27-0.32)	974(924-1082)
	Gambia, The	87(73-125)	0.03(0.03-0.05)	24(20-35)
	Ghana	5609(4678-7784)	0.07(0.06-0.09)	135(113-188)
	Guinea	687(543-1086)	0.03(0.02-0.05)	36(28-56)
	Guinea-Bissau	155(132-238)	0.09(0.08-0.14)	57(48-87)
	Kenya	10366(9691-11762)	0.09(0.09-0.11)	142(133-161)
	Lesotho	203(176-284)	0.17(0.15-0.24)	84(73-118)
	Madagascar	682(542-974)	0.04(0.03-0.06)	17(13-24)
	Mali	988(862-1305)	0.05(0.04-0.06)	32(28-42)
	Mauritania	577(518-744)	0.05(0.05-0.07)	86(77-110)
	Mozambique	4283(4144-4762)	0.23(0.23-0.26)	91(88-101)
	Namibia	1259(1198-1432)	0.22(0.21-0.25)	387(368-440)
	Niger	1261(1203-1422)	0.07(0.07-0.08)	30(28-33)
	Nigeria	12008(10478-15669)	0.04(0.03-0.05)	40(35-53)
	Rwanda	1042(874-1534)	0.06(0.05-0.08)	58(49-85)
	Senegal	7118(6815-7865)	0.22(0.21-0.24)	291(278-321)
	Sierra Leone	100(85-143)	0.03(0.02-0.04)	10(8-14)
	South Africa	40508(38153-44856)	0.21(0.19-0.23)	593(558-656)
	Sudan	5168(4657-6601)	0.10(0.09-0.13)	84(75-107)
	Togo	355(287-566)	0.04(0.03-0.06)	30(25-48)
	Uganda	4154(3752-5067)	0.08(0.07-0.10)	62(56-75)
	Zambia	1198(854-2202)	0.05(0.04-0.10)	43(30-78)
	Zimbabwe	2421(2210-2884)	0.16(0.15-0.20)	126(115-150)

Table S6. Total macroeconomic cost, per capita economic cost, and economic cost as a percentage of GDP in 2020–2050 attributable to diabetes mellitus, by country and World Bank region in 2017 international dollars, with discount rate of 0%, without informal care, and with baseline (lower bound, upper bound) mortality and morbidity.

Region	World Bank Country	Economic cost in millions of 2017 INT$	Percentage of total GDP in 2020-2050	Per capita loss in 2017 INT$
**East Asia & Pacific**	American Samoa*	168(124-230)	0.511(0.378-0.699)	3,090(2,284-4,225)
**East Asia & Pacific**	Australia	108,830(86,758-137,675)	0.193(0.154-0.244)	3,712(2,959-4,696)
**East Asia & Pacific**	Brunei Darussalam	3,463(2,257-5,094)	0.341(0.222-0.502)	7,279(4,745-10,706)
**East Asia & Pacific**	Cambodia	13,108(8,672-19,368)	0.220(0.146-0.325)	670(443-990)
**East Asia & Pacific**	China	2,425,796(1,901,335-3,106,548)	0.175(0.137-0.224)	1,676(1,314-2,147)
**East Asia & Pacific**	Fiji	3,033(1,835-4,866)	0.667(0.403-1.070)	3,056(1,848-4,903)
**East Asia & Pacific**	Guam*	782(559-1,085)	0.270(0.193-0.375)	4,248(3,039-5,900)
**East Asia & Pacific**	Indonesia	714,692(480,016-1,024,880)	0.339(0.228-0.487)	2,327(1,563-3,338)
**East Asia & Pacific**	Japan	373,249(293,901-470,651)	0.214(0.169-0.270)	3,195(2,516-4,029)
**East Asia & Pacific**	Kiribati*	71(50-99)	0.594(0.417-0.825)	482(338-669)
**East Asia & Pacific**	Korea, Dem. People's Rep.*	3,415(2,429-4,768)	0.171(0.121-0.238)	128(91-179)
**East Asia & Pacific**	Korea, Rep.	274,701(216,765-354,930)	0.268(0.212-0.347)	5,486(4,329-7,088)
**East Asia & Pacific**	Lao PDR	7,889(4,658-12,426)	0.194(0.114-0.305)	924(545-1,455)
**East Asia & Pacific**	Malaysia	94,588(63,554-138,587)	0.180(0.121-0.263)	2,546(1,711-3,730)
**East Asia & Pacific**	Marshall Islands*	51(36-73)	0.494(0.342-0.699)	757(524-1,071)
**East Asia & Pacific**	Micronesia, Fed. Sts.*	93(55-145)	0.645(0.380-1.007)	717(422-1,120)
**East Asia & Pacific**	Mongolia	1,749(1,174-2,585)	0.075(0.050-0.111)	449(301-664)
**East Asia & Pacific**	Myanmar*	40,090(32,562-51,409)	0.262(0.213-0.336)	675(548-865)
**East Asia & Pacific**	Nauru*	22(15-34)	0.385(0.260-0.588)	2,044(1,383-3,122)
**East Asia & Pacific**	New Zealand	21,020(16,114-26,911)	0.196(0.150-0.251)	3,982(3,053-5,098)
**East Asia & Pacific**	Northern Mariana Islands*	247(180-336)	0.397(0.290-0.541)	4,024(2,936-5,481)
**East Asia & Pacific**	Palau*	73(52-101)	0.755(0.535-1.040)	3,997(2,833-5,506)
**East Asia & Pacific**	Papua New Guinea*	6,401(4,611-8,975)	0.335(0.242-0.470)	552(398-774)
**East Asia & Pacific**	Philippines	155,400(106,041-211,998)	0.226(0.154-0.308)	1,205(822-1,644)
**East Asia & Pacific**	Samoa*	190(136-264)	0.344(0.247-0.479)	818(587-1,137)
**East Asia & Pacific**	Singapore	49,146(39,587-63,744)	0.188(0.152-0.244)	7,813(6,294-10,134)
**East Asia & Pacific**	Solomon Islands*	375(279-502)	0.491(0.365-0.658)	386(287-517)
**East Asia & Pacific**	Taiwan (Province of China)*	150,395(105,148-219,685)	0.263(0.184-0.384)	6,371(4,454-9,306)
**East Asia & Pacific**	Thailand	102,677(71,528-144,655)	0.176(0.123-0.249)	1,484(1,034-2,091)
**East Asia & Pacific**	Timor-Leste*	321(220-449)	0.174(0.119-0.244)	191(131-267)
**East Asia & Pacific**	Tonga*	117(84-161)	0.417(0.300-0.576)	968(697-1,339)

Region	World Bank Country	Economic cost in millions of 2017 INT$	Percentage of total GDP in 2020-2050	Per capita loss in 2017 INT$
**East Asia & Pacific**	Tuvalu*	20(13-28)	0.511(0.350-0.727)	1,410(967-2,007)
**East Asia & Pacific**	Vanuatu*	138(98-194)	0.332(0.234-0.465)	324(228-454)
**East Asia & Pacific**	Vietnam	189,569(119,232-285,886)	0.255(0.160-0.384)	1,801(1,133-2,715)
**Europe & Central Asia**	Albania	2,462(1,596-3,634)	0.132(0.085-0.195)	914(592-1,349)
**Europe & Central Asia**	Andorra*	291(209-403)	0.175(0.125-0.242)	3,756(2,688-5,193)
**Europe & Central Asia**	Armenia	5,649(4,290-7,452)	0.261(0.198-0.344)	1,932(1,467-2,549)
**Europe & Central Asia**	Austria	29,744(24,147-37,177)	0.162(0.132-0.203)	3,247(2,636-4,059)
**Europe & Central Asia**	Azerbaijan	9,289(6,252-13,770)	0.182(0.123-0.270)	858(578-1,273)
**Europe & Central Asia**	Belarus	4,475(3,162-6,376)	0.080(0.057-0.114)	492(348-702)
**Europe & Central Asia**	Belgium	37,513(29,272-49,242)	0.172(0.134-0.225)	3,130(2,443-4,109)
**Europe & Central Asia**	Bosnia and Herzegovina	7,856(5,560-11,059)	0.339(0.240-0.477)	2,607(1,845-3,670)
**Europe & Central Asia**	Bulgaria	30,144(21,469-42,459)	0.397(0.282-0.559)	4,900(3,490-6,902)
**Europe & Central Asia**	Croatia	10,584(7,613-14,830)	0.197(0.142-0.277)	2,824(2,031-3,957)
**Europe & Central Asia**	Cyprus	3,870(3,222-4,741)	0.187(0.156-0.229)	2,989(2,489-3,662)
**Europe & Central Asia**	Czech Republic	119,378(90,679-157,326)	0.577(0.438-0.761)	11,188(8,498-14,744)
**Europe & Central Asia**	Denmark	26,123(20,468-33,474)	0.185(0.145-0.238)	4,313(3,379-5,526)
**Europe & Central Asia**	Estonia	5,841(4,321-7,998)	0.236(0.175-0.323)	4,681(3,463-6,409)
**Europe & Central Asia**	Finland	24,413(19,487-30,798)	0.239(0.191-0.301)	4,398(3,510-5,548)
**Europe & Central Asia**	France	128,703(99,075-167,434)	0.118(0.091-0.154)	1,923(1,480-2,501)
**Europe & Central Asia**	Georgia	4,169(3,209-5,448)	0.134(0.103-0.175)	1,107(852-1,446)
**Europe & Central Asia**	Germany	727,015(560,519-935,786)	0.443(0.342-0.571)	8,822(6,801-11,355)
**Europe & Central Asia**	Greece	11,910(8,833-16,008)	0.114(0.084-0.153)	1,226(909-1,647)
**Europe & Central Asia**	Greenland*	199(142-278)	0.165(0.117-0.231)	3,552(2,531-4,969)
**Europe & Central Asia**	Hungary	43,603(31,478-60,219)	0.264(0.191-0.365)	4,788(3,457-6,613)
**Europe & Central Asia**	Iceland	1,765(1,246-2,466)	0.178(0.126-0.249)	4,852(3,426-6,779)
**Europe & Central Asia**	Ireland	159,808(128,702-202,003)	0.270(0.218-0.342)	29,846(24,037-37,726)
**Europe & Central Asia**	Italy	150,930(120,953-189,695)	0.185(0.149-0.233)	2,606(2,088-3,275)
**Europe & Central Asia**	Kazakhstan	38,647(28,182-52,853)	0.167(0.122-0.228)	1,798(1,311-2,459)
**Europe & Central Asia**	Kyrgyz Republic	2,487(1,725-3,520)	0.153(0.106-0.217)	316(219-447)
**Europe & Central Asia**	Latvia	5,496(3,849-7,923)	0.200(0.140-0.288)	3,311(2,319-4,773)
**Europe & Central Asia**	Lithuania	8,788(6,865-11,420)	0.167(0.130-0.217)	3,672(2,868-4,772)
**Europe & Central Asia**	Luxembourg	4,667(3,534-6,221)	0.140(0.106-0.187)	6,526(4,942-8,700)
**Europe & Central Asia**	Moldova	2,807(2,039-3,877)	0.164(0.119-0.226)	750(544-1,035)

Region	World Bank Country	Economic cost in millions of 2017 INT$	Percentage of total GDP in 2020-2050	Per capita loss in 2017 INT$
**Europe & Central Asia**	Monaco*	755(543-1,025)	0.197(0.141-0.267)	17,646(12,676-23,940)
**Europe & Central Asia**	Montenegro	1,904(1,309-2,725)	0.332(0.228-0.475)	3,097(2,130-4,433)
**Europe & Central Asia**	Netherlands	78,274(62,154-99,355)	0.197(0.156-0.250)	4,507(3,579-5,721)
**Europe & Central Asia**	North Macedonia	4,056(2,715-6,020)	0.264(0.177-0.392)	2,030(1,359-3,013)
**Europe & Central Asia**	Norway	32,675(27,385-39,107)	0.237(0.199-0.284)	5,396(4,522-6,458)
**Europe & Central Asia**	Poland	214,382(162,135-285,117)	0.306(0.231-0.407)	5,961(4,508-7,928)
**Europe & Central Asia**	Portugal	23,918(18,987-30,657)	0.172(0.137-0.221)	2,464(1,956-3,158)
**Europe & Central Asia**	Romania*	64,202(46,559-88,805)	0.198(0.144-0.274)	3,609(2,618-4,993)
**Europe & Central Asia**	Russian Federation	124,905(90,958-165,645)	0.101(0.073-0.133)	885(644-1,174)
**Europe & Central Asia**	San Marino*	148(106-205)	0.190(0.136-0.263)	4,326(3,084-5,985)
**Europe & Central Asia**	Serbia	23,565(16,960-33,694)	0.349(0.251-0.499)	2,964(2,133-4,238)
**Europe & Central Asia**	Slovak Republic	23,574(16,700-33,594)	0.289(0.205-0.412)	4,460(3,160-6,356)
**Europe & Central Asia**	Slovenia	7,397(5,523-9,893)	0.184(0.137-0.246)	3,652(2,727-4,885)
**Europe & Central Asia**	Spain	161,200(126,983-210,169)	0.218(0.171-0.284)	3,536(2,785-4,610)
**Europe & Central Asia**	Sweden	49,863(42,182-59,360)	0.212(0.179-0.252)	4,618(3,906-5,497)
**Europe & Central Asia**	Switzerland	57,080(43,961-74,610)	0.240(0.185-0.314)	6,117(4,711-7,996)
**Europe & Central Asia**	Tajikistan	9,292(6,440-12,672)	0.313(0.217-0.427)	728(505-993)
**Europe & Central Asia**	Turkey	162,094(122,406-218,177)	0.112(0.085-0.151)	1,773(1,339-2,386)
**Europe & Central Asia**	Turkmenistan*	6,795(4,560-9,727)	0.198(0.133-0.283)	961(645-1,376)
**Europe & Central Asia**	Ukraine	1,471(979-2,102)	0.045(0.030-0.064)	37(25-53)
**Europe & Central Asia**	United Kingdom	351,415(275,077-447,566)	0.306(0.240-0.390)	4,925(3,855-6,272)
**Europe & Central Asia**	Uzbekistan	62,499(39,756-92,437)	0.329(0.209-0.486)	1,610(1,024-2,382)
**Latin America & Caribbean**	Antigua and Barbuda*	326(248-429)	0.373(0.285-0.492)	3,064(2,337-4,041)
**Latin America & Caribbean**	Argentina	55,123(44,327-69,073)	0.180(0.144-0.225)	1,092(878-1,368)
**Latin America & Caribbean**	Bahamas, The	1,620(1,058-2,367)	0.364(0.238-0.532)	3,718(2,428-5,433)
**Latin America & Caribbean**	Barbados	661(415-990)	0.506(0.318-0.758)	2,310(1,448-3,455)
**Latin America & Caribbean**	Belize	355(243-502)	0.381(0.261-0.540)	719(492-1,017)
**Latin America & Caribbean**	Bolivia	10,255(6,645-15,024)	0.216(0.140-0.317)	738(478-1,081)
**Latin America & Caribbean**	Brazil	139,831(116,850-166,575)	0.144(0.120-0.171)	622(520-741)
**Latin America & Caribbean**	Chile	44,185(35,995-55,208)	0.235(0.191-0.294)	2,232(1,818-2,789)
**Latin America & Caribbean**	Colombia	81,089(58,382-113,782)	0.247(0.178-0.347)	1,499(1,079-2,103)
**Latin America & Caribbean**	Costa Rica	14,833(10,541-20,670)	0.286(0.203-0.398)	2,678(1,903-3,731)
**Latin America & Caribbean**	Cuba*	20,029(14,478-27,398)	0.292(0.211-0.400)	1,838(1,329-2,515)

Region	World Bank Country	Economic cost in millions of 2017 INT$	Percentage of total GDP in 2020-2050	Per capita loss in 2017 INT$
Latin America & Caribbean	Dominica*	130(94-179)	0.460(0.331-0.633)	1,796(1,294-2,471)
Latin America & Caribbean	Dominican Republic	51,892(29,694-83,871)	0.371(0.212-0.600)	4,317(2,471-6,978)
Latin America & Caribbean	Ecuador	10,864(8,299-14,619)	0.167(0.128-0.225)	525(401-706)
Latin America & Caribbean	El Salvador	6,342(3,662-10,365)	0.281(0.162-0.459)	931(537-1,521)
Latin America & Caribbean	Grenada*	333(258-430)	0.423(0.328-0.545)	2,885(2,235-3,716)
Latin America & Caribbean	Guatemala	20,095(14,118-28,386)	0.264(0.185-0.373)	887(623-1,253)
Latin America & Caribbean	Guyana*	9,929(6,688-14,590)	0.426(0.287-0.627)	12,080(8,137-17,752)
Latin America & Caribbean	Haiti*	3,072(2,114-4,499)	0.259(0.178-0.379)	231(159-339)
Latin America & Caribbean	Honduras	6,119(4,294-8,753)	0.221(0.155-0.316)	508(357-727)
Latin America & Caribbean	Jamaica	3,965(2,420-5,970)	0.427(0.261-0.643)	1,312(801-1,975)
Latin America & Caribbean	Mexico	341,059(255,928-440,869)	0.376(0.282-0.486)	2,362(1,772-3,053)
Latin America & Caribbean	Nicaragua*	3,563(2,531-5,082)	0.236(0.167-0.336)	464(330-662)
Latin America & Caribbean	Panama	22,105(16,579-29,589)	0.339(0.254-0.454)	4,286(3,215-5,737)
Latin America & Caribbean	Paraguay	10,774(7,111-15,913)	0.250(0.165-0.369)	1,308(863-1,932)
Latin America & Caribbean	Peru	21,246(15,336-30,178)	0.119(0.086-0.168)	572(413-813)
Latin America & Caribbean	Puerto Rico*	12,725(9,008-18,280)	0.438(0.310-0.629)	4,628(3,276-6,648)
Latin America & Caribbean	St. Kitts and Nevis*	204(148-276)	0.384(0.279-0.520)	3,649(2,649-4,949)
Latin America & Caribbean	St. Lucia*	430(328-575)	0.504(0.385-0.674)	2,301(1,757-3,078)
Latin America & Caribbean	St. Vincent and the Grenadines*	319(246-414)	0.543(0.419-0.705)	2,846(2,197-3,696)
Latin America & Caribbean	Suriname	965(666-1,368)	0.365(0.251-0.517)	1,498(1,033-2,124)
Latin America & Caribbean	Trinidad and Tobago*	6,672(3,981-9,949)	0.706(0.421-1.053)	4,785(2,855-7,135)
Latin America & Caribbean	Uruguay	3,385(2,662-4,325)	0.120(0.094-0.153)	944(742-1,206)
Latin America & Caribbean	Venezuela, RB*	41,558(26,002-62,129)	0.309(0.193-0.462)	1,218(762-1,820)
Latin America & Caribbean	Virgin Islands (U.S.)*	922(665-1,254)	0.476(0.343-0.648)	9,511(6,858-12,937)
Middle East & North Africa	Algeria*	37,377(26,490-51,496)	0.196(0.139-0.270)	707(501-974)
Middle East & North Africa	Bahrain	7,745(6,267-9,736)	0.240(0.194-0.302)	3,725(3,015-4,683)
Middle East & North Africa	Djibouti	620(357-981)	0.134(0.077-0.213)	535(308-846)
Middle East & North Africa	Egypt, Arab Rep.	172,829(119,071-249,806)	0.205(0.141-0.296)	1,321(910-1,910)
Middle East & North Africa	Iran, Islamic Rep.*	93,867(70,495-118,896)	0.216(0.162-0.274)	987(741-1,251)
Middle East & North Africa	Iraq	36,490(28,834-46,430)	0.182(0.144-0.231)	658(520-837)
Middle East & North Africa	Israel	46,314(34,929-61,887)	0.230(0.173-0.307)	4,342(3,275-5,802)
Middle East & North Africa	Jordan	5,151(3,786-7,264)	0.118(0.087-0.166)	453(333-639)
Middle East & North Africa	Kuwait	18,096(15,546-21,586)	0.272(0.234-0.325)	3,679(3,160-4,388)

Region	World Bank Country	Economic cost in millions of 2017 INT$	Percentage of total GDP in 2020-2050	Per capita loss in 2017 INT$
**Middle East & North Africa**	Lebanon	482(285-747)	0.092(0.054-0.142)	75(45-117)
**Middle East & North Africa**	Libya*	34,695(23,418-50,379)	0.213(0.144-0.309)	4,431(2,990-6,433)
**Middle East & North Africa**	Malta	5,620(4,696-6,907)	0.352(0.294-0.433)	12,727(10,635-15,641)
**Middle East & North Africa**	Morocco	19,754(15,081-26,731)	0.157(0.120-0.213)	468(358-634)
**Middle East & North Africa**	Oman	13,905(8,895-20,640)	0.215(0.137-0.318)	2,265(1,449-3,362)
**Middle East & North Africa**	Qatar	16,005(13,297-19,839)	0.162(0.134-0.200)	4,643(3,857-5,755)
**Middle East & North Africa**	Saudi Arabia	158,500(131,781-195,619)	0.242(0.202-0.299)	3,907(3,248-4,822)
**Middle East & North Africa**	Syrian Arab Republic*	4,804(3,319-6,962)	0.191(0.132-0.277)	175(121-253)
**Middle East & North Africa**	Tunisia	9,902(7,684-12,738)	0.218(0.169-0.280)	762(592-981)
**Middle East & North Africa**	United Arab Emirates*	58,341(38,404-86,995)	0.195(0.128-0.290)	5,554(3,656-8,282)
**Middle East & North Africa**	Yemen, Rep.*	4,450(3,013-6,464)	0.121(0.082-0.176)	113(76-164)
**North America**	Bermuda*	741(550-1,005)	0.283(0.210-0.384)	12,630(9,373-17,132)
**North America**	Canada	132,967(102,866-171,362)	0.188(0.145-0.242)	3,164(2,447-4,077)
**North America**	United States	3,749,807(3,215,813-4,391,682)	0.436(0.374-0.511)	10,495(9,001-12,292)
**South Asia**	Afghanistan*	4,015(2,555-5,896)	0.165(0.105-0.242)	77(49-113)
**South Asia**	Bangladesh	106,714(66,055-165,909)	0.132(0.081-0.205)	585(362-910)
**South Asia**	Bhutan	1,046(628-1,646)	0.192(0.115-0.303)	1,221(733-1,922)
**South Asia**	India	1,589,291(1,114,625-2,193,880)	0.214(0.150-0.296)	1,034(725-1,427)
**South Asia**	Maldives	697(482-1,000)	0.143(0.099-0.205)	1,276(883-1,831)
**South Asia**	Nepal	13,434(9,076-19,361)	0.175(0.118-0.252)	400(270-576)
**South Asia**	Pakistan	137,347(88,170-202,999)	0.219(0.141-0.324)	488(313-721)
**South Asia**	Sri Lanka	55,832(32,700-95,743)	0.406(0.238-0.696)	2,541(1,488-4,358)
**Sub-Saharan Africa**	Angola	7,355(4,312-11,670)	0.112(0.065-0.177)	139(81-220)
**Sub-Saharan Africa**	Benin	2,688(1,607-4,314)	0.090(0.054-0.145)	151(90-242)
**Sub-Saharan Africa**	Botswana	2,904(2,178-3,904)	0.169(0.127-0.227)	981(736-1,319)
**Sub-Saharan Africa**	Burkina Faso	4,718(2,974-7,029)	0.138(0.087-0.205)	150(94-223)
**Sub-Saharan Africa**	Burundi	263(171-391)	0.076(0.050-0.114)	14(9-22)
**Sub-Saharan Africa**	Cabo Verde	222(149-322)	0.124(0.083-0.181)	354(237-514)
**Sub-Saharan Africa**	Cameroon	6,056(3,650-9,786)	0.101(0.061-0.164)	159(96-257)
**Sub-Saharan Africa**	Central African Republic*	422(297-610)	0.165(0.116-0.239)	64(45-93)
**Sub-Saharan Africa**	Chad*	1,008(694-1,446)	0.114(0.079-0.164)	41(28-58)
**Sub-Saharan Africa**	Comoros	159(90-252)	0.122(0.069-0.194)	136(77-216)
**Sub-Saharan Africa**	Congo, Dem. Rep.	9,642(5,935-14,882)	0.137(0.084-0.212)	69(43-107)

Region	World Bank Country	Economic cost in millions of 2017 INT$	Percentage of total GDP in 2020-2050	Per capita loss in 2017 INT$
**Sub-Saharan Africa**	Congo, Rep.	591(352-943)	0.134(0.080-0.214)	74(44-118)
**Sub-Saharan Africa**	Cote d'Ivoire	10,537(6,539-16,166)	0.080(0.050-0.123)	276(171-424)
**Sub-Saharan Africa**	Equatorial Guinea*	578(395-825)	0.151(0.103-0.216)	274(187-391)
**Sub-Saharan Africa**	Eritrea*	689(474-999)	0.142(0.098-0.206)	146(101-212)
**Sub-Saharan Africa**	Eswatini	640(384-991)	0.157(0.094-0.243)	453(272-702)
**Sub-Saharan Africa**	Ethiopia	23,906(15,633-35,480)	0.074(0.048-0.110)	149(98-221)
**Sub-Saharan Africa**	Gabon	1,902(1,307-2,800)	0.134(0.092-0.197)	632(434-930)
**Sub-Saharan Africa**	Gambia, The	317(191-509)	0.088(0.053-0.141)	88(53-141)
**Sub-Saharan Africa**	Ghana	19,352(11,661-31,090)	0.162(0.097-0.260)	467(282-751)
**Sub-Saharan Africa**	Guinea	3,340(2,017-5,292)	0.100(0.061-0.159)	173(105-274)
**Sub-Saharan Africa**	Guinea-Bissau	302(195-452)	0.127(0.082-0.190)	110(71-165)
**Sub-Saharan Africa**	Kenya	12,229(8,577-17,106)	0.077(0.054-0.108)	168(118-235)
**Sub-Saharan Africa**	Lesotho	399(237-623)	0.252(0.150-0.394)	165(98-258)
**Sub-Saharan Africa**	Liberia*	366(249-536)	0.128(0.087-0.188)	51(35-75)
**Sub-Saharan Africa**	Madagascar	2,075(1,262-3,175)	0.094(0.057-0.144)	52(31-79)
**Sub-Saharan Africa**	Malawi*	2,066(1,432-2,963)	0.123(0.085-0.176)	73(51-105)
**Sub-Saharan Africa**	Mali	2,192(1,311-3,561)	0.073(0.044-0.119)	70(42-114)
**Sub-Saharan Africa**	Mauritania	868(540-1,372)	0.057(0.036-0.090)	129(80-204)
**Sub-Saharan Africa**	Mauritius	6,507(5,022-8,625)	0.557(0.430-0.738)	5,202(4,015-6,895)
**Sub-Saharan Africa**	Mozambique	3,696(2,551-5,472)	0.141(0.098-0.209)	78(54-116)
**Sub-Saharan Africa**	Namibia	814(549-1,214)	0.104(0.070-0.156)	250(169-373)
**Sub-Saharan Africa**	Niger	1,824(1,181-2,776)	0.070(0.046-0.107)	43(28-65)
**Sub-Saharan Africa**	Nigeria	16,295(11,103-23,733)	0.040(0.027-0.058)	55(37-80)
**Sub-Saharan Africa**	Rwanda	3,370(1,993-5,331)	0.126(0.074-0.199)	188(111-297)
**Sub-Saharan Africa**	Sao Tome and Principe*	55(38-79)	0.119(0.083-0.171)	183(127-263)
**Sub-Saharan Africa**	Senegal	5,900(4,047-8,559)	0.124(0.085-0.180)	241(165-349)
**Sub-Saharan Africa**	Seychelles*	458(332-626)	0.322(0.234-0.440)	4,448(3,224-6,079)
**Sub-Saharan Africa**	Sierra Leone	246(148-390)	0.050(0.030-0.079)	24(14-37)
**Sub-Saharan Africa**	Somalia*	1,301(901-1,887)	0.130(0.090-0.188)	53(37-77)
**Sub-Saharan Africa**	South Africa	42,571(34,227-52,729)	0.160(0.128-0.198)	623(501-772)
**Sub-Saharan Africa**	South Sudan*	1,255(862-1,805)	0.125(0.086-0.180)	81(56-117)
**Sub-Saharan Africa**	Sudan	8,805(6,145-12,643)	0.122(0.085-0.176)	142(99-204)
**Sub-Saharan Africa**	Tanzania	18,679(10,701-30,165)	0.138(0.079-0.223)	203(116-328)

Region	World Bank Country	Economic cost in millions of 2017 INT$	Percentage of total GDP in 2020-2050	Per capita loss in 2017 INT$
**Sub-Saharan Africa**	Togo	1,491(911-2,357)	0.114(0.069-0.180)	128(78-202)
**Sub-Saharan Africa**	Uganda	9,844(5,935-15,528)	0.129(0.078-0.203)	147(88-231)
**Sub-Saharan Africa**	Zambia	3,859(2,285-6,162)	0.125(0.074-0.200)	137(81-219)
**Sub-Saharan Africa**	Zimbabwe	2,207(1,411-3,315)	0.110(0.070-0.165)	115(73-172)
**Others**	Cook Islands*	96(72-129)	0.643(0.481-0.863)	5,533(4,141-7,426)
**Others**	Niue*	4(3-5)	0.771(0.551-1.047)	2,278(1,627-3,094)
**Others**	Palestine*	2,975(2,274-3,925)	0.230(0.176-0.303)	427(326-563)
**Others**	Tokelau*	2(1-2)	0.462(0.318-0.639)	1,208(833-1,673)

Table S7. Total macroeconomic cost, per capita economic cost, and economic cost as a percentage of GDP in 2020–2050 attributable to diabetes mellitus, by country and World Bank region in 2017 international dollars, with discount rate of 3%, without informal care, and with baseline (lower bound, upper bound) mortality and morbidity.

Region	World Bank Country	Economic cost in millions of 2017 INT$	Percentage of total GDP in 2020-2050	Per capita loss in 2017 INT$
**East Asia & Pacific**	American Samoa*	91(67-124)	0.451(0.333-0.616)	1,668(1,233-2,276)
**East Asia & Pacific**	Australia	58,943(46,914-74,593)	0.169(0.134-0.214)	2,011(1,600-2,544)
**East Asia & Pacific**	Brunei Darussalam	1,971(1,291-2,886)	0.301(0.197-0.441)	4,142(2,713-6,065)
**East Asia & Pacific**	Cambodia	6,644(4,401-9,804)	0.199(0.132-0.293)	340(225-501)
**East Asia & Pacific**	China	1,301,555(1,021,875-1,663,019)	0.156(0.123-0.200)	900(706-1,149)
**East Asia & Pacific**	Fiji	1,699(1,038-2,697)	0.614(0.375-0.974)	1,711(1,045-2,717)
**East Asia & Pacific**	Guam*	422(302-585)	0.238(0.170-0.330)	2,291(1,639-3,177)
**East Asia & Pacific**	Indonesia	368,837(248,951-526,881)	0.300(0.202-0.428)	1,201(811-1,716)
**East Asia & Pacific**	Japan	207,237(163,366-261,047)	0.185(0.146-0.233)	1,774(1,398-2,235)
**East Asia & Pacific**	Kiribati*	39(27-54)	0.524(0.368-0.726)	262(184-363)
**East Asia & Pacific**	Korea, Dem. People's Rep.*	1,840(1,309-2,567)	0.150(0.107-0.210)	69(49-97)
**East Asia & Pacific**	Korea, Rep.	148,095(116,936-191,100)	0.235(0.186-0.304)	2,957(2,335-3,816)
**East Asia & Pacific**	Lao PDR	4,056(2,397-6,382)	0.174(0.103-0.274)	475(281-747)
**East Asia & Pacific**	Malaysia	49,090(33,064-71,705)	0.159(0.107-0.232)	1,321(890-1,930)
**East Asia & Pacific**	Marshall Islands*	28(19-39)	0.436(0.301-0.616)	409(283-577)
**East Asia & Pacific**	Micronesia, Fed. Sts.*	52(30-81)	0.569(0.335-0.887)	399(235-622)
**East Asia & Pacific**	Mongolia	922(618-1,361)	0.067(0.045-0.099)	237(159-350)
**East Asia & Pacific**	Myanmar*	20,308(16,499-26,003)	0.231(0.188-0.296)	342(278-438)
**East Asia & Pacific**	Nauru*	12(8-19)	0.339(0.230-0.517)	1,141(772-1,739)
**East Asia & Pacific**	New Zealand	11,243(8,630-14,377)	0.173(0.132-0.221)	2,130(1,635-2,724)

Region	World Bank Country	Economic cost in millions of 2017 INT$	Percentage of total GDP in 2020-2050	Per capita loss in 2017 INT$
**East Asia & Pacific**	Northern Mariana Islands*	133(97-181)	0.351(0.256-0.477)	2,171(1,584-2,953)
**East Asia & Pacific**	Palau*	41(29-56)	0.667(0.472-0.916)	2,249(1,594-3,092)
**East Asia & Pacific**	Papua New Guinea*	3,389(2,441-4,744)	0.296(0.213-0.414)	292(211-409)
**East Asia & Pacific**	Philippines	78,583(54,006-106,929)	0.202(0.139-0.274)	610(419-829)
**East Asia & Pacific**	Samoa*	102(73-142)	0.304(0.218-0.421)	441(316-611)
**East Asia & Pacific**	Singapore	26,360(21,213-34,163)	0.166(0.133-0.215)	4,191(3,373-5,431)
**East Asia & Pacific**	Solomon Islands*	201(149-269)	0.433(0.322-0.579)	207(154-277)
**East Asia & Pacific**	Taiwan (Province of China)*	81,108(56,711-118,309)	0.232(0.162-0.338)	3,436(2,402-5,012)
**East Asia & Pacific**	Thailand	54,196(37,808-76,242)	0.154(0.107-0.216)	783(547-1,102)
**East Asia & Pacific**	Timor-Leste*	181(124-253)	0.154(0.105-0.215)	107(74-150)
**East Asia & Pacific**	Tonga*	64(46-88)	0.368(0.265-0.508)	531(382-734)
**East Asia & Pacific**	Tuvalu*	10(7-14)	0.451(0.309-0.640)	710(487-1,008)
**East Asia & Pacific**	Vanuatu*	74(52-103)	0.293(0.207-0.410)	173(122-242)
**East Asia & Pacific**	Vietnam	96,384(60,901-144,938)	0.231(0.146-0.347)	916(578-1,377)
**Europe & Central Asia**	Albania	1,300(844-1,915)	0.114(0.074-0.168)	482(313-711)
**Europe & Central Asia**	Andorra*	157(112-217)	0.154(0.110-0.213)	2,024(1,449-2,796)
**Europe & Central Asia**	Armenia	3,008(2,282-3,967)	0.236(0.179-0.311)	1,029(781-1,357)
**Europe & Central Asia**	Austria	16,292(13,192-20,407)	0.141(0.114-0.177)	1,779(1,440-2,228)
**Europe & Central Asia**	Azerbaijan	5,343(3,597-7,899)	0.163(0.110-0.241)	494(332-730)
**Europe & Central Asia**	Belarus	2,595(1,835-3,687)	0.071(0.050-0.101)	286(202-406)
**Europe & Central Asia**	Belgium	20,594(16,038-27,043)	0.149(0.116-0.196)	1,718(1,338-2,257)
**Europe & Central Asia**	Bosnia and Herzegovina	4,213(2,981-5,921)	0.298(0.211-0.419)	1,398(989-1,965)
**Europe & Central Asia**	Bulgaria	15,932(11,408-22,307)	0.344(0.246-0.482)	2,590(1,855-3,626)
**Europe & Central Asia**	Croatia	5,733(4,114-8,039)	0.175(0.126-0.246)	1,530(1,098-2,145)
**Europe & Central Asia**	Cyprus	1,959(1,622-2,412)	0.162(0.134-0.199)	1,513(1,253-1,863)
**Europe & Central Asia**	Czech Republic	61,907(46,969-81,624)	0.497(0.377-0.655)	5,802(4,402-7,649)
**Europe & Central Asia**	Denmark	14,264(11,174-18,273)	0.163(0.128-0.209)	2,355(1,845-3,017)
**Europe & Central Asia**	Estonia	3,093(2,285-4,233)	0.208(0.153-0.284)	2,479(1,831-3,392)
**Europe & Central Asia**	Finland	13,348(10,630-16,868)	0.207(0.165-0.262)	2,404(1,915-3,038)
**Europe & Central Asia**	France	70,039(53,942-91,034)	0.102(0.078-0.132)	1,046(806-1,360)
**Europe & Central Asia**	Georgia	2,200(1,691-2,875)	0.119(0.091-0.155)	584(449-763)
**Europe & Central Asia**	Germany	391,795(303,019-503,099)	0.378(0.293-0.486)	4,754(3,677-6,105)
**Europe & Central Asia**	Greece	6,590(4,862-8,898)	0.098(0.072-0.132)	678(500-916)

Region	World Bank Country	Economic cost in millions of 2017 INT$	Percentage of total GDP in 2020-2050	Per capita loss in 2017 INT$
**Europe & Central Asia**	Greenland*	107(76-150)	0.145(0.103-0.203)	1,914(1,364-2,675)
**Europe & Central Asia**	Hungary	23,296(16,826-32,126)	0.236(0.170-0.325)	2,558(1,848-3,528)
**Europe & Central Asia**	Iceland	928(655-1,296)	0.157(0.111-0.220)	2,552(1,801-3,563)
**Europe & Central Asia**	Ireland	77,540(62,383-98,081)	0.245(0.197-0.309)	14,481(11,651-18,318)
**Europe & Central Asia**	Italy	83,405(66,664-104,978)	0.160(0.128-0.201)	1,440(1,151-1,812)
**Europe & Central Asia**	Kazakhstan	21,049(15,372-28,733)	0.149(0.108-0.203)	980(715-1,337)
**Europe & Central Asia**	Kyrgyz Republic	1,286(893-1,817)	0.133(0.092-0.187)	164(114-231)
**Europe & Central Asia**	Latvia	2,934(2,053-4,222)	0.175(0.123-0.252)	1,768(1,237-2,543)
**Europe & Central Asia**	Lithuania	4,677(3,648-6,081)	0.146(0.114-0.190)	1,954(1,524-2,541)
**Europe & Central Asia**	Luxembourg	2,543(1,928-3,386)	0.124(0.094-0.166)	3,556(2,696-4,735)
**Europe & Central Asia**	Moldova	1,506(1,094-2,077)	0.145(0.105-0.200)	402(292-555)
**Europe & Central Asia**	Monaco*	407(292-552)	0.173(0.125-0.235)	9,512(6,834-12,889)
**Europe & Central Asia**	Montenegro	997(685-1,427)	0.290(0.199-0.415)	1,622(1,115-2,322)
**Europe & Central Asia**	Netherlands	42,629(33,830-54,113)	0.172(0.136-0.218)	2,454(1,948-3,116)
**Europe & Central Asia**	North Macedonia	2,222(1,495-3,277)	0.236(0.159-0.348)	1,112(748-1,640)
**Europe & Central Asia**	Norway	18,119(15,185-21,681)	0.207(0.174-0.248)	2,992(2,508-3,581)
**Europe & Central Asia**	Poland	112,217(84,997-148,686)	0.270(0.204-0.357)	3,120(2,363-4,134)
**Europe & Central Asia**	Portugal	12,910(10,185-16,617)	0.150(0.118-0.193)	1,330(1,049-1,712)
**Europe & Central Asia**	Romania*	33,433(24,250-46,189)	0.175(0.127-0.242)	1,880(1,363-2,597)
**Europe & Central Asia**	Russian Federation	70,432(51,390-93,237)	0.088(0.064-0.116)	499(364-661)
**Europe & Central Asia**	San Marino*	82(59-114)	0.168(0.119-0.231)	2,406(1,715-3,325)
**Europe & Central Asia**	Serbia	12,499(8,985-17,828)	0.305(0.219-0.435)	1,572(1,130-2,242)
**Europe & Central Asia**	Slovak Republic	12,271(8,703-17,429)	0.248(0.176-0.352)	2,322(1,647-3,298)
**Europe & Central Asia**	Slovenia	3,972(2,954-5,327)	0.163(0.121-0.219)	1,961(1,459-2,630)
**Europe & Central Asia**	Spain	86,066(67,486-112,568)	0.189(0.148-0.247)	1,888(1,480-2,469)
**Europe & Central Asia**	Sweden	27,096(22,895-32,286)	0.186(0.157-0.222)	2,509(2,120-2,990)
**Europe & Central Asia**	Switzerland	31,423(24,230-41,008)	0.210(0.162-0.275)	3,368(2,597-4,395)
**Europe & Central Asia**	Tajikistan	4,598(3,184-6,281)	0.274(0.190-0.375)	360(250-492)
**Europe & Central Asia**	Turkey	86,681(65,515-116,405)	0.100(0.076-0.135)	948(717-1,273)
**Europe & Central Asia**	Turkmenistan*	3,837(2,575-5,486)	0.174(0.117-0.249)	543(364-776)
**Europe & Central Asia**	Ukraine	993(665-1,410)	0.036(0.024-0.051)	25(17-36)
**Europe & Central Asia**	United Kingdom	189,641(148,131-241,809)	0.264(0.206-0.337)	2,658(2,076-3,389)
**Europe & Central Asia**	Uzbekistan	32,329(20,731-47,576)	0.295(0.189-0.434)	833(534-1,226)

Region	World Bank Country	Economic cost in millions of 2017 INT$	Percentage of total GDP in 2020-2050	Per capita loss in 2017 INT$
**Latin America & Caribbean**	Antigua and Barbuda*	169(129-223)	0.329(0.251-0.433)	1,594(1,216-2,099)
**Latin America & Caribbean**	Argentina	30,867(24,825-38,638)	0.154(0.124-0.193)	611(492-765)
**Latin America & Caribbean**	Bahamas, The	900(589-1,312)	0.319(0.209-0.465)	2,066(1,353-3,011)
**Latin America & Caribbean**	Barbados	370(234-550)	0.443(0.280-0.658)	1,292(816-1,921)
**Latin America & Caribbean**	Belize	195(134-275)	0.334(0.229-0.471)	395(272-557)
**Latin America & Caribbean**	Bolivia	5,467(3,546-8,006)	0.192(0.125-0.281)	394(255-576)
**Latin America & Caribbean**	Brazil	80,147(66,920-95,493)	0.125(0.105-0.149)	356(298-425)
**Latin America & Caribbean**	Chile	23,758(19,344-29,671)	0.202(0.164-0.252)	1,200(977-1,499)
**Latin America & Caribbean**	Colombia	43,661(31,438-61,099)	0.216(0.156-0.302)	807(581-1,129)
**Latin America & Caribbean**	Costa Rica	7,846(5,583-10,914)	0.249(0.177-0.347)	1,416(1,008-1,970)
**Latin America & Caribbean**	Cuba*	10,803(7,810-14,755)	0.258(0.186-0.352)	991(717-1,354)
**Latin America & Caribbean**	Dominica*	73(53-100)	0.406(0.292-0.557)	1,008(726-1,385)
**Latin America & Caribbean**	Dominican Republic	26,336(15,154-42,407)	0.329(0.189-0.529)	2,191(1,261-3,528)
**Latin America & Caribbean**	Ecuador	6,056(4,616-8,139)	0.145(0.110-0.195)	292(223-393)
**Latin America & Caribbean**	El Salvador	3,448(2,014-5,583)	0.246(0.144-0.399)	506(296-819)
**Latin America & Caribbean**	Grenada*	177(137-227)	0.373(0.289-0.480)	1,529(1,185-1,967)
**Latin America & Caribbean**	Guatemala	10,649(7,511-14,970)	0.231(0.163-0.325)	470(332-661)
**Latin America & Caribbean**	Guyana*	5,166(3,479-7,578)	0.376(0.253-0.552)	6,285(4,233-9,220)
**Latin America & Caribbean**	Haiti*	1,724(1,186-2,521)	0.228(0.157-0.333)	130(89-190)
**Latin America & Caribbean**	Honduras	3,221(2,254-4,613)	0.194(0.136-0.278)	267(187-383)
**Latin America & Caribbean**	Jamaica	2,178(1,343-3,260)	0.368(0.227-0.551)	721(444-1,079)
**Latin America & Caribbean**	Mexico	184,503(138,948-237,925)	0.325(0.245-0.419)	1,278(962-1,647)
**Latin America & Caribbean**	Nicaragua*	1,982(1,408-2,823)	0.208(0.148-0.296)	258(183-368)
**Latin America & Caribbean**	Panama	11,442(8,601-15,284)	0.298(0.224-0.398)	2,219(1,668-2,963)
**Latin America & Caribbean**	Paraguay	5,729(3,802-8,420)	0.217(0.144-0.319)	696(462-1,022)
**Latin America & Caribbean**	Peru	11,205(8,095-15,864)	0.103(0.074-0.145)	302(218-427)
**Latin America & Caribbean**	Puerto Rico*	7,598(5,379-10,897)	0.387(0.274-0.554)	2,763(1,956-3,963)
**Latin America & Caribbean**	St. Kitts and Nevis*	111(81-150)	0.338(0.246-0.458)	1,986(1,442-2,689)
**Latin America & Caribbean**	St. Lucia*	237(181-317)	0.444(0.339-0.593)	1,269(969-1,694)
**Latin America & Caribbean**	St. Vincent and the Grenadines*	174(134-226)	0.479(0.369-0.620)	1,554(1,200-2,014)
**Latin America & Caribbean**	Suriname	553(383-783)	0.320(0.221-0.452)	859(595-1,215)
**Latin America & Caribbean**	Trinidad and Tobago*	3,957(2,361-5,890)	0.623(0.372-0.928)	2,838(1,693-4,224)
**Latin America & Caribbean**	Uruguay	1,875(1,475-2,394)	0.104(0.082-0.133)	523(411-668)

Region	World Bank Country	Economic cost in millions of 2017 INT$	Percentage of total GDP in 2020-2050	Per capita loss in 2017 INT$
**Latin America & Caribbean**	Venezuela, RB*	22,417(14,025-33,462)	0.272(0.170-0.407)	657(411-980)
**Latin America & Caribbean**	Virgin Islands (U.S.)*	497(359-676)	0.420(0.303-0.570)	5,132(3,701-6,969)
**Middle East & North Africa**	Algeria*	20,623(14,618-28,379)	0.173(0.123-0.238)	390(277-537)
**Middle East & North Africa**	Bahrain	4,074(3,289-5,131)	0.206(0.166-0.259)	1,960(1,582-2,468)
**Middle East & North Africa**	Djibouti	321(185-508)	0.123(0.071-0.196)	277(160-438)
**Middle East & North Africa**	Egypt, Arab Rep.	89,150(61,686-128,399)	0.180(0.124-0.259)	682(472-982)
**Middle East & North Africa**	Iran, Islamic Rep.*	52,462(39,408-66,360)	0.191(0.143-0.241)	552(415-698)
**Middle East & North Africa**	Iraq	18,978(15,004-24,142)	0.161(0.127-0.204)	342(271-435)
**Middle East & North Africa**	Israel	24,726(18,699-32,918)	0.204(0.154-0.272)	2,318(1,753-3,086)
**Middle East & North Africa**	Jordan	2,862(2,101-4,023)	0.105(0.077-0.148)	252(185-354)
**Middle East & North Africa**	Kuwait	9,959(8,530-11,912)	0.232(0.198-0.277)	2,025(1,734-2,422)
**Middle East & North Africa**	Lebanon	309(183-477)	0.075(0.044-0.115)	48(29-75)
**Middle East & North Africa**	Libya*	15,273(10,309-22,149)	0.187(0.127-0.272)	1,950(1,317-2,828)
**Middle East & North Africa**	Malta	2,828(2,364-3,471)	0.314(0.263-0.386)	6,404(5,354-7,860)
**Middle East & North Africa**	Morocco	10,501(7,990-14,235)	0.137(0.104-0.186)	249(189-337)
**Middle East & North Africa**	Oman	7,350(4,702-10,894)	0.182(0.116-0.269)	1,197(766-1,775)
**Middle East & North Africa**	Qatar	8,734(7,245-10,831)	0.140(0.116-0.174)	2,534(2,102-3,142)
**Middle East & North Africa**	Saudi Arabia	85,565(70,987-105,716)	0.208(0.173-0.257)	2,109(1,750-2,606)
**Middle East & North Africa**	Syrian Arab Republic*	2,589(1,789-3,748)	0.168(0.116-0.244)	94(65-136)
**Middle East & North Africa**	Tunisia	5,363(4,150-6,922)	0.187(0.145-0.241)	413(319-533)
**Middle East & North Africa**	United Arab Emirates*	31,363(20,646-46,714)	0.172(0.113-0.255)	2,986(1,966-4,447)
**Middle East & North Africa**	Yemen, Rep.*	2,396(1,622-3,478)	0.106(0.072-0.154)	61(41-88)
**North America**	Bermuda*	400(297-541)	0.250(0.185-0.338)	6,812(5,056-9,226)
**North America**	Canada	72,820(56,324-93,797)	0.164(0.127-0.211)	1,733(1,340-2,232)
**North America**	United States	2,060,116(1,765,780-2,412,768)	0.386(0.331-0.452)	5,766(4,942-6,753)
**South Asia**	Afghanistan*	2,253(1,433-3,305)	0.145(0.092-0.213)	43(27-63)
**South Asia**	Bangladesh	54,722(34,019-84,654)	0.121(0.075-0.187)	300(187-464)
**South Asia**	Bhutan	534(321-838)	0.170(0.102-0.267)	623(374-978)
**South Asia**	India	808,636(569,291-1,112,547)	0.193(0.136-0.266)	526(370-724)
**South Asia**	Maldives	348(241-500)	0.126(0.087-0.180)	638(441-915)
**South Asia**	Nepal	6,886(4,654-9,920)	0.155(0.105-0.223)	205(138-295)
**South Asia**	Pakistan	72,760(46,842-107,202)	0.196(0.126-0.289)	258(166-381)
**South Asia**	Sri Lanka	30,278(17,881-51,258)	0.365(0.215-0.617)	1,378(814-2,333)

Region	World Bank Country	Economic cost in millions of 2017 INT$	Percentage of total GDP in 2020-2050	Per capita loss in 2017 INT$
**Sub-Saharan Africa**	Angola	4,331(2,553-6,838)	0.100(0.059-0.158)	82(48-129)
**Sub-Saharan Africa**	Benin	1,440(863-2,303)	0.083(0.050-0.132)	81(48-129)
**Sub-Saharan Africa**	Botswana	1,561(1,167-2,104)	0.149(0.111-0.200)	527(394-711)
**Sub-Saharan Africa**	Burkina Faso	2,448(1,545-3,645)	0.124(0.078-0.184)	78(49-116)
**Sub-Saharan Africa**	Burundi	150(98-225)	0.068(0.044-0.101)	8(5-12)
**Sub-Saharan Africa**	Cabo Verde	119(80-172)	0.111(0.075-0.162)	189(127-274)
**Sub-Saharan Africa**	Cameroon	3,253(1,965-5,238)	0.092(0.055-0.148)	86(52-138)
**Sub-Saharan Africa**	Central African Republic*	222(156-320)	0.146(0.102-0.210)	34(24-49)
**Sub-Saharan Africa**	Chad*	576(396-826)	0.101(0.069-0.144)	23(16-33)
**Sub-Saharan Africa**	Comoros	86(49-136)	0.108(0.062-0.172)	74(42-117)
**Sub-Saharan Africa**	Congo, Dem. Rep.	5,174(3,192-7,965)	0.126(0.077-0.193)	37(23-57)
**Sub-Saharan Africa**	Congo, Rep.	379(226-602)	0.121(0.073-0.193)	48(28-76)
**Sub-Saharan Africa**	Cote d'Ivoire	5,383(3,340-8,252)	0.073(0.045-0.112)	141(88-216)
**Sub-Saharan Africa**	Equatorial Guinea*	383(262-547)	0.133(0.091-0.190)	181(124-259)
**Sub-Saharan Africa**	Eritrea*	371(255-538)	0.125(0.086-0.181)	79(54-114)
**Sub-Saharan Africa**	Eswatini	367(219-568)	0.144(0.086-0.223)	260(155-402)
**Sub-Saharan Africa**	Ethiopia	12,135(7,982-17,905)	0.069(0.045-0.102)	76(50-112)
**Sub-Saharan Africa**	Gabon	1,045(718-1,536)	0.118(0.081-0.174)	347(239-510)
**Sub-Saharan Africa**	Gambia, The	167(101-267)	0.079(0.048-0.127)	46(28-74)
**Sub-Saharan Africa**	Ghana	10,304(6,226-16,481)	0.148(0.090-0.237)	249(150-398)
**Sub-Saharan Africa**	Guinea	1,696(1,025-2,681)	0.091(0.055-0.144)	88(53-139)
**Sub-Saharan Africa**	Guinea-Bissau	161(104-241)	0.116(0.075-0.173)	59(38-88)
**Sub-Saharan Africa**	Kenya	6,433(4,526-8,965)	0.069(0.048-0.096)	88(62-123)
**Sub-Saharan Africa**	Lesotho	229(137-358)	0.221(0.132-0.345)	95(57-148)
**Sub-Saharan Africa**	Liberia*	207(141-303)	0.113(0.077-0.165)	29(20-43)
**Sub-Saharan Africa**	Madagascar	1,116(680-1,706)	0.084(0.051-0.129)	28(17-42)
**Sub-Saharan Africa**	Malawi*	1,087(754-1,558)	0.108(0.075-0.155)	39(27-55)
**Sub-Saharan Africa**	Mali	1,161(697-1,874)	0.067(0.040-0.107)	37(22-60)
**Sub-Saharan Africa**	Mauritania	461(287-727)	0.052(0.032-0.081)	68(43-108)
**Sub-Saharan Africa**	Mauritius	3,445(2,652-4,564)	0.488(0.376-0.647)	2,754(2,120-3,649)
**Sub-Saharan Africa**	Mozambique	1,927(1,330-2,847)	0.125(0.086-0.185)	41(28-60)
**Sub-Saharan Africa**	Namibia	462(311-688)	0.092(0.062-0.137)	142(96-211)
**Sub-Saharan Africa**	Niger	932(605-1,415)	0.063(0.041-0.095)	22(14-33)

Region	World Bank Country	Economic cost in millions of 2017 INT$	Percentage of total GDP in 2020-2050	Per capita loss in 2017 INT$
**Sub-Saharan Africa**	Nigeria	9,853(6,710-14,344)	0.038(0.026-0.055)	33(23-48)
**Sub-Saharan Africa**	Rwanda	1,724(1,023-2,721)	0.115(0.068-0.182)	96(57-152)
**Sub-Saharan Africa**	Sao Tome and Principe*	29(20-42)	0.105(0.073-0.150)	98(68-140)
**Sub-Saharan Africa**	Senegal	3,014(2,069-4,364)	0.111(0.076-0.161)	123(84-178)
**Sub-Saharan Africa**	Seychelles*	240(174-327)	0.284(0.206-0.388)	2,324(1,685-3,172)
**Sub-Saharan Africa**	Sierra Leone	142(85-224)	0.044(0.027-0.070)	14(8-21)
**Sub-Saharan Africa**	Somalia*	689(478-999)	0.114(0.079-0.166)	28(19-41)
**Sub-Saharan Africa**	South Africa	24,023(19,224-29,818)	0.141(0.113-0.175)	352(281-436)
**Sub-Saharan Africa**	South Sudan*	676(464-972)	0.110(0.076-0.159)	44(30-63)
**Sub-Saharan Africa**	Sudan	4,877(3,403-6,999)	0.108(0.075-0.154)	79(55-113)
**Sub-Saharan Africa**	Tanzania	9,611(5,543-15,444)	0.126(0.073-0.202)	105(60-168)
**Sub-Saharan Africa**	Togo	797(487-1,258)	0.105(0.064-0.165)	68(42-108)
**Sub-Saharan Africa**	Uganda	5,148(3,112-8,102)	0.117(0.071-0.184)	77(46-121)
**Sub-Saharan Africa**	Zambia	2,134(1,270-3,389)	0.114(0.068-0.181)	76(45-121)
**Sub-Saharan Africa**	Zimbabwe	1,252(802-1,875)	0.098(0.063-0.147)	65(42-97)
**Others**	Cook Islands*	52(39-70)	0.567(0.424-0.760)	2,987(2,235-4,000)
**Others**	Niue*	2(1-3)	0.680(0.486-0.922)	1,230(878-1,667)
**Others**	Palestine*	1,604(1,226-2,114)	0.202(0.155-0.267)	230(176-303)
**Others**	Tokelau*	1(1-1)	0.407(0.281-0.563)	652(450-901)

Table S8. Total macroeconomic cost, per capita economic cost, and economic cost as a percentage of GDP in 2020–2050 attributable to diabetes mellitus, by country and World Bank region in 2017 international dollars, with discount rate of 2%, with baseline mortality and morbidity, and with 4.0 baseline (0.283 lower bound-8.3 upper bound) informal care hours weekly.

Region	World Bank Country	Economic cost in millions of 2017 INT$	Percentage of total GDP in 2020-2050	Per capita loss in 2017 INT$
**East Asia & Pacific**	American Samoa*	792(55-1,519)	3.364(0.253,6.448)	14,553(1,107,27,893)
**East Asia & Pacific**	Australia	508,145(37,206-975,571)	1.249(0.096,2.397)	17,333(1,111,33,278)
**East Asia & Pacific**	Brunei Darussalam	19,030(1,451-36,884)	2.529(0.160,4.902)	39,996(3,049,77,521)
**East Asia & Pacific**	Cambodia	64,560(4,678-124,930)	1.602(0.113,3.099)	3,301(255,6,388)
**East Asia & Pacific**	China	11,008,850(799,300-21,111,462)	1.123(0.080,2.153)	7,608(594,14,590)
**East Asia & Pacific**	Fiji	14,186(1,103-27,061)	4.371(0.337,8.338)	14,291(959,27,263)
**East Asia & Pacific**	Guam*	3,869(301-7,452)	1.866(0.134,3.594)	21,028(1,444,40,499)
**East Asia & Pacific**	Indonesia	1,963,047(144,474-3,573,983)	1.342(0.101,2.444)	6,393(512,11,639)
**East Asia & Pacific**	Japan	2,042,232(139,826-3,960,570)	1.584(0.114,3.072)	17,482(1,142,33,903)

**East Asia & Pacific**	Kiribati*	336(26-643)	3.879(0.256,7.428)	2,264(173,4,336)
**East Asia & Pacific**	Korea, Dem. People's Rep.*	17,916(1,323-34,688)	1.251(0.083,2.423)	674(51.767564,1,305)
**East Asia & Pacific**	Korea, Rep.	1,308,158(87,667-2,513,942)	1.779(0.120,3.419)	26,124(1,748,50,204)
**East Asia & Pacific**	Lao PDR	35,407(2,645-67,991)	1.269(0.090,2.437)	4,147(286,7,963)
**East Asia & Pacific**	Malaysia	512,273(37,250-996,050)	1.397(0.103,2.717)	13,788(1,048,26,809)
**East Asia & Pacific**	Marshall Islands*	242(18-465)	3.255(0.217,6.24)	3,572(276,6,848)
**East Asia & Pacific**	Micronesia, Fed. Sts.*	441(33-845)	4.193(0.274,8.028)	3,405(257,6,518)
**East Asia & Pacific**	Mongolia	8,629(652-16,667)	0.531(0.041,1.026)	2,216(160,4,281)
**East Asia & Pacific**	Myanmar*	190,996(12,529-367,952)	1.817(0.118,3.5)	3,215(240,6,194)
**East Asia & Pacific**	Nauru*	110(8-211)	2.579(0.166,4.952)	10,029(693,19,258)
**East Asia & Pacific**	New Zealand	111,624(8,143-216,597)	1.461(0.102,2.834)	21,148(1,389,41,036)
**East Asia & Pacific**	Northern Mariana Islands*	1,181(83-2,268)	2.657(0.180,5.1)	19,258(1,408,36,971)
**East Asia & Pacific**	Palau*	347(25-664)	4.877(0.315,9.329)	18,994(1,352,36,337)
**East Asia & Pacific**	Papua New Guinea*	30,663(2,173-58,941)	2.273(0.170,4.368)	2,645(200,5,084)
**East Asia & Pacific**	Philippines	449,245(35,256-825,188)	0.959(0.067,1.761)	3,484(255,6,400)
**East Asia & Pacific**	Samoa*	919(61-1,766)	2.328(0.166,4.474)	3,955(305,7,600)
**East Asia & Pacific**	Singapore	216,371(14,215-413,551)	1.161(0.082,2.219)	34,399(2,604,65,748)
**East Asia & Pacific**	Solomon Islands*	1,762(135-3,378)	3.238(0.212,6.207)	1,812(135.8216,3,474)
**East Asia & Pacific**	Taiwan (Province of China)*	746,490(50,414-1,438,040)	1.824(0.131,3.514)	31,623(2,342,60,919)
**East Asia & Pacific**	Thailand	690,407(45,357-1,354,504)	1.667(0.111,3.27)	9,980(658,19,579)
**East Asia & Pacific**	Timor-Leste*	1,730(129-3,348)	1.275(0.093,2.467)	1,027(65.91817573,1,987)
**East Asia & Pacific**	Tonga*	563(42-1,080)	2.777(0.194,5.329)	4,670(359,8,962)
**East Asia & Pacific**	Tuvalu*	88(6-169)	3.361(0.239,6.442)	6,343(457,12,158)
**East Asia & Pacific**	Vanuatu*	668(45-1,284)	2.253(0.176,4.331)	1,564(104.1807121,3,006)
**East Asia & Pacific**	Vietnam	841,876(61,390-1,616,434)	1.672(0.130,3.21)	7,997(587,15,354)
**Europe & Central Asia**	Albania	15,741(1,069-30,907)	1.179(0.077,2.316)	5,842(370,11,471)
**Europe & Central Asia**	Andorra*	1,523(113-2,948)	1.279(0.092,2.475)	19,630(1,350,37,993)
**Europe & Central Asia**	Armenia	44,154(3,040-87,433)	2.922(0.195,5.785)	15,101(989,29,903)
**Europe & Central Asia**	Austria	206,256(13,353-405,856)	1.543(0.108,3.036)	22,517(1,492,44,307)
**Europe & Central Asia**	Azerbaijan	68,231(4,743-134,571)	1.807(0.117,3.563)	6,306(444,12,438)
**Europe & Central Asia**	Belarus	29,511(1,918-57,875)	0.704(0.047,1.381)	3,248(230,6,369)
**Europe & Central Asia**	Belgium	305,156(18,532-604,980)	1.911(0.128,3.788)	25,464(1,662,50,483)
**Europe & Central Asia**	Bosnia and Herzegovina	56,273(4,039-110,926)	3.399(0.238,6.7)	18,676(1,310,36,815)
**Europe & Central Asia**	Bulgaria	99,998(7,264-186,064)	1.843(0.150,3.429)	16,256(1,098,30,248)
**Europe & Central Asia**	Croatia	103,764(7,323-207,324)	2.71(0.183,5.415)	27,685(1,972,55,316)

**Europe & Central Asia**	Cyprus	21,507(1,407-41,853)	1.494(0.105,2.907)	16,614(1,055,32,330)
**Europe & Central Asia**	Czech Republic	536,156(39,261-1,024,600)	3.66(0.237,6.993)	50,246(3,465,96,021)
**Europe & Central Asia**	Denmark	166,823(10,334-327,039)	1.639(0.106,3.213)	27,540(1,979,53,988)
**Europe & Central Asia**	Estonia	21,054(1,555-39,552)	1.201(0.087,2.256)	16,871(1,343,31,695)
**Europe & Central Asia**	Finland	165,106(11,277-324,325)	2.212(0.143,4.345)	29,741(2,033,58,422)
**Europe & Central Asia**	France	880,031(57,984-1,731,979)	1.104(0.070,2.173)	13,147(816,25,875)
**Europe & Central Asia**	Georgia	15,269(1,234-28,751)	0.697(0.050,1.312)	4,053(291,7,632)
**Europe & Central Asia**	Germany	4,013,173(261,136-7,787,985)	3.347(0.255,6.495)	48,697(3,358,94,501)
**Europe & Central Asia**	Greece	116,069(7,034-230,942)	1.501(0.100,2.986)	11,943(776,23,763)
**Europe & Central Asia**	Greenland*	1,049(68-2,032)	1.215(0.082,2.353)	18,739(1,350,36,300)
**Europe & Central Asia**	Hungary	208,902(15,198-402,108)	1.792(0.138,3.449)	22,941(1,474,44,158)
**Europe & Central Asia**	Iceland	12,453(773-24,585)	1.787(0.127,3.528)	34,227(2,424,67,568)
**Europe & Central Asia**	Ireland	876,568(64,225-1,709,450)	2.258(0.150,4.403)	163,709(10,625,319,258)
**Europe & Central Asia**	Italy	982,387(71,655-1,922,969)	1.635(0.118,3.2)	16,959(1,232,33,196)
**Europe & Central Asia**	Kazakhstan	266,908(16,898-524,672)	1.61(0.109,3.164)	12,420(862,24,415)
**Europe & Central Asia**	Kyrgyz Republic	15,754(1,132-30,919)	1.377(0.094,2.703)	2,003(132,3,931)
**Europe & Central Asia**	Latvia	24,385(1,725-46,649)	1.243(0.097,2.378)	14,688(1,047,28,099)
**Europe & Central Asia**	Lithuania	31,646(2,152-59,405)	0.844(0.061,1.585)	13,223(1,027,24,822)
**Europe & Central Asia**	Luxembourg	65,360(4,589-131,769)	2.736(0.168,5.516)	91,400(5,977,184,265)
**Europe & Central Asia**	Moldova	19,958(1,449-39,374)	1.637(0.101,3.23)	5,329(387,10,513)
**Europe & Central Asia**	Monaco*	3,882(272-7,502)	1.413(0.103,2.732)	90,697(6,444,175,279)
**Europe & Central Asia**	Montenegro	9,071(636-17,461)	2.238(0.167,4.309)	14,756(973,28,404)
**Europe & Central Asia**	Netherlands	426,830(31,023-828,559)	1.479(0.109,2.871)	24,576(1,739,47,706)
**Europe & Central Asia**	North Macedonia	30,410(1,860-60,091)	2.762(0.182,5.458)	15,220(1,118,30,075)
**Europe & Central Asia**	Norway	190,170(12,861-370,104)	1.882(0.127,3.663)	31,406(2,023,61,121)
**Europe & Central Asia**	Poland	1,156,835(76,825-2,246,489)	2.351(0.172,4.565)	32,166(2,426,62,465)
**Europe & Central Asia**	Portugal	203,086(12,594-402,924)	2.024(0.134,4.016)	20,917(1,288,41,500)
**Europe & Central Asia**	Romania*	322,189(22,809-622,595)	1.423(0.107,2.75)	18,114(1,182,35,002)
**Europe & Central Asia**	Russian Federation	876,544(54,419-1,726,011)	0.952(0.059,1.875)	6,210(411,12,228)
**Europe & Central Asia**	San Marino*	780(50-1,509)	1.371(0.099,2.652)	22,807(1,499,44,095)
**Europe & Central Asia**	Serbia	109,999(7,319-211,164)	2.288(0.162,4.391)	13,836(995,26,561)
**Europe & Central Asia**	Slovak Republic	97,952(6,723-186,529)	1.686(0.123,3.21)	18,533(1,292,35,292)
**Europe & Central Asia**	Slovenia	52,077(3,364-102,680)	1.821(0.129,3.59)	25,714(1,705,50,701)
**Europe & Central Asia**	Spain	1,038,038(64,156-2,034,586)	1.95(0.138,3.822)	22,768(1,579,44,626)
**Europe & Central Asia**	Sweden	243,710(16,216-469,390)	1.435(0.093,2.763)	22,569(1,752,43,469)

**Europe & Central Asia**	Switzerland	299,472(22,540-579,640)	1.73(0.130,3.348)	32,094(2,059,62,120)
**Europe & Central Asia**	Tajikistan	49,272(3,614-95,293)	2.443(0.159,4.725)	3,861(295,7,468)
**Europe & Central Asia**	Turkey	979,741(67,946-1,915,290)	0.962(0.063,1.881)	10,715(705,20,947)
**Europe & Central Asia**	Turkmenistan*	36,001(2,760-69,571)	1.418(0.091,2.74)	5,092(382,9,840)
**Europe & Central Asia**	Ukraine	18,630(1,208-37,363)	0.641(0.039,1.285)	472(31.82360863,947)
**Europe & Central Asia**	United Kingdom	2,381,413(162,915-4,673,457)	2.855(0.210,5.604)	33,374(2,414,65,495)
**Europe & Central Asia**	Uzbekistan	284,077(19,803-545,728)	2.169(0.149,4.168)	7,320(556,14,062)
**Latin America & Caribbean**	Antigua and Barbuda*	1,529(99-2,936)	2.506(0.175,4.813)	14,386(1,049,27,631)
**Latin America & Caribbean**	Argentina	263,277(18,609-504,592)	1.148(0.084,2.201)	5,214(397,9,992)
**Latin America & Caribbean**	Bahamas, The	6,551(488-12,399)	2.008(0.147,3.8)	15,035(1,176,28,457)
**Latin America & Caribbean**	Barbados	2,938(218-5,597)	3.049(0.205,5.808)	10,260(675,19,544)
**Latin America & Caribbean**	Belize	1,099(82-2,023)	1.623(0.125,2.987)	2,228(166,4,101)
**Latin America & Caribbean**	Bolivia	39,011(2,574-73,686)	1.164(0.084,2.199)	2,808(203,5,304)
**Latin America & Caribbean**	Brazil	822,037(57,619-1,599,212)	1.126(0.076,2.19)	3,655(255,7,111)
**Latin America & Caribbean**	Chile	212,864(16,460-408,989)	1.558(0.115,2.993)	10,752(736,20,658)
**Latin America & Caribbean**	Colombia	405,521(30,882-782,861)	1.719(0.126,3.318)	7,496(476,14,472)
**Latin America & Caribbean**	Costa Rica	88,765(6,204-173,462)	2.403(0.159,4.696)	16,023(1,076,31,311)
**Latin America & Caribbean**	Cuba*	98,363(7,345-189,297)	2.006(0.140,3.86)	9,027(679,17,373)
**Latin America & Caribbean**	Dominica*	634(43-1,215)	3.044(0.221,5.838)	8,741(586,16,763)
**Latin America & Caribbean**	Dominican Republic	135,099(10,021-244,773)	1.409(0.099,2.553)	11,240(946,20,365)
**Latin America & Caribbean**	Ecuador	41,429(2,987-77,935)	0.861(0.069,1.619)	2,001(145,3,764)
**Latin America & Caribbean**	El Salvador	33,609(2,472-65,099)	2.061(0.146,3.991)	4,932(356,9,554)
**Latin America & Caribbean**	Grenada*	1,570(116-3,013)	2.814(0.219,5.401)	13,586(1,022,26,070)
**Latin America & Caribbean**	Guatemala	71,465(4,936-133,808)	1.32(0.093,2.472)	3,155(234,5,908)
**Latin America & Caribbean**	Guyana*	46,202(3,078-88,653)	2.837(0.212,5.443)	56,213(3,612,107,863)
**Latin America & Caribbean**	Haiti*	15,683(1,038-30,216)	1.796(0.130,3.461)	1,181(87.84790524,2,276)
**Latin America & Caribbean**	Honduras	29,698(2,242-57,270)	1.517(0.097,2.925)	2,466(159,4,755)
**Latin America & Caribbean**	Jamaica	14,231(1,050-26,612)	2.083(0.150,3.896)	4,708(372,8,804)
**Latin America & Caribbean**	Mexico	1,255,214(91,081-2,349,757)	1.904(0.137,3.564)	8,691(596,16,270)
**Latin America & Caribbean**	Nicaragua*	18,277(1,300-35,249)	1.654(0.115,3.189)	2,380(174,4,590)
**Latin America & Caribbean**	Panama	75,725(5,106-141,372)	1.663(0.123,3.105)	14,682(1,109,27,410)
**Latin America & Caribbean**	Paraguay	35,845(2,423-66,735)	1.161(0.090,2.162)	4,352(326,8,103)
**Latin America & Caribbean**	Peru	88,545(6,435-168,388)	0.692(0.052,1.316)	2,385(179,4,536)
**Latin America & Caribbean**	Puerto Rico*	64,727(4,473-124,178)	2.91(0.190,5.582)	23,540(1,645,45,162)
**Latin America & Caribbean**	St. Kitts and Nevis*	983(65-1,888)	2.572(0.179,4.94)	17,611(1,182,33,818)

**Latin America & Caribbean**	St. Lucia*	2,056(141-3,940)	3.316(0.214,6.356)	11,003(811,21,092)
**Latin America & Caribbean**	St. Vincent and the Grenadines*	1,508(112-2,889)	3.557(0.273,6.815)	13,462(902,25,794)
**Latin America & Caribbean**	Suriname	5,589(381-10,861)	2.822(0.192,5.483)	8,678(613,16,864)
**Latin America & Caribbean**	Trinidad and Tobago*	32,934(2,457-63,022)	4.572(0.330,8.75)	23,618(1,561,45,196)
**Latin America & Caribbean**	Uruguay	26,164(1,799-51,675)	1.258(0.089,2.484)	7,297(519,14,411)
**Latin America & Caribbean**	Venezuela, RB*	203,045(15,112-390,565)	2.108(0.146,4.055)	5,949(419,11,443)
**Latin America & Caribbean**	Virgin Islands (U.S.)*	4,360(321-8,360)	3.146(0.246,6.031)	44,977(3,165,86,242)
**Middle East & North Africa**	Algeria*	195,187(13,965-377,231)	1.409(0.095,2.723)	3,693(276,7,137)
**Middle East & North Africa**	Bahrain	32,596(2,397-61,805)	1.409(0.096,2.672)	15,678(1,181,29,728)
**Middle East & North Africa**	Djibouti	2,632(171-5,031)	0.841(0.066,1.607)	2,269(156,4,338)
**Middle East & North Africa**	Egypt, Arab Rep.	1,168,511(76,457-2,294,017)	1.986(0.146,3.899)	8,933(611,17,537)
**Middle East & North Africa**	Iran, Islamic Rep.*	488,096(37,435-942,233)	1.534(0.110,2.961)	5,134(346,9,911)
**Middle East & North Africa**	Iraq	180,487(12,872-347,861)	1.288(0.091,2.483)	3,255(247,6,274)
**Middle East & North Africa**	Israel	172,059(12,701-324,001)	1.206(0.088,2.271)	16,130(1,199,30,375)
**Middle East & North Africa**	Jordan	53,516(3,225-106,952)	1.688(0.110,3.374)	4,710(334,9,413)
**Middle East & North Africa**	Kuwait	60,661(4,875-112,163)	1.228(0.097,2.271)	12,332(1,018,22,802)
**Middle East & North Africa**	Lebanon	8,200(512-16,548)	1.842(0.115,3.718)	1,282(83.27191807,2,586)
**Middle East & North Africa**	Libya*	154,794(11,596-298,877)	1.511(0.102,2.918)	19,767(1,330,38,166)
**Middle East & North Africa**	Malta	26,341(1,743-50,754)	2.431(0.165,4.684)	59,651(4,261,114,935)
**Middle East & North Africa**	Morocco	217,597(13,419-435,515)	2.422(0.169,4.848)	5,158(340,10,324)
**Middle East & North Africa**	Oman	104,234(6,771-206,070)	2.216(0.151,4.381)	16,980(1,248,33,569)
**Middle East & North Africa**	Qatar	61,433(4,582-115,312)	0.851(0.064,1.597)	17,821(1,279,33,451)
**Middle East & North Africa**	Saudi Arabia	684,771(47,916-1,299,983)	1.437(0.095,2.728)	16,878(1,302,32,041)
**Middle East & North Africa**	Syrian Arab Republic*	24,793(1,682-47,932)	1.378(0.099,2.663)	901(63.85753867,1,741)
**Middle East & North Africa**	Tunisia	74,726(5,034-147,384)	2.25(0.148,4.437)	5,753(385,11,346)
**Middle East & North Africa**	United Arab Emirates*	299,801(21,740-579,469)	1.4(0.096,2.705)	28,542(2,133,55,167)
**Middle East & North Africa**	Yemen, Rep.*	24,832(1,865-48,330)	0.942(0.063,1.834)	631(40.91491089,1,227)
**North America**	Bermuda*	3,649(256-7,025)	1.949(0.144,3.752)	62,219(3,990,119,775)
**North America**	Canada	787,776(55,658-1,536,797)	1.526(0.112,2.977)	18,743(1,251,36,563)
**North America**	United States	16,538,637(1,132,070-31,548,891)	2.66(0.196,5.075)	46,289(3,116,88,301)
**South Asia**	Afghanistan*	21,742(1,589-42,115)	1.216(0.093,2.355)	416(26.53180469,805)
**South Asia**	Bangladesh	657,358(46,717-1,290,233)	1.204(0.085,2.362)	3,606(267,7,077)
**South Asia**	Bhutan	5,102(366-9,862)	1.361(0.095,2.63)	5,955(408,11,511)
**South Asia**	India	11,439,017(706,349-22,593,731)	2.272(0.168,4.488)	7,441(505,14,697)
**South Asia**	Maldives	5,082(345-10,059)	1.529(0.111,3.026)	9,304(652,18,415)

**South Asia**	Nepal	66,939(4,631-129,608)	1.26(0.097,2.44)	1,991(131,3,855)
**South Asia**	Pakistan	623,765(45,016-1,197,246)	1.422(0.109,2.729)	2,214(144,4,250)
**South Asia**	Sri Lanka	412,865(28,889-815,194)	4.23(0.268,8.352)	18,791(1,366,37,103)
**Sub-Saharan Africa**	Angola	28,445(2,065-53,469)	0.574(0.046,1.08)	536(38.06741648,1,007)
**Sub-Saharan Africa**	Benin	11,751(760-22,477)	0.568(0.043,1.087)	658(43.87470383,1,259)
**Sub-Saharan Africa**	Botswana	7,321(548-13,108)	0.595(0.049,1.066)	2,473(183,4,428)
**Sub-Saharan Africa**	Burkina Faso	16,765(1,155-31,513)	0.71(0.052,1.335)	532(36.18379314,1,001)
**Sub-Saharan Africa**	Burundi	811(67-1,488)	0.318(0.023,0.584)	45(3.440789744,82)
**Sub-Saharan Africa**	Cabo Verde	1,500(96-2,954)	1.192(0.080,2.348)	2,390(177,4,707)
**Sub-Saharan Africa**	Cameroon	18,619(1,468-34,333)	0.444(0.034,0.82)	490(39.2585932,903)
**Sub-Saharan Africa**	Central African Republic*	2,190(147-4,241)	1.217(0.086,2.357)	333(22.25378779,644)
**Sub-Saharan Africa**	Chad*	5,924(397-11,540)	0.901(0.061,1.756)	239(15.87517583,465)
**Sub-Saharan Africa**	Comoros	540(37-1,007)	0.581(0.039,1.084)	463(37.15165915,863)
**Sub-Saharan Africa**	Congo, Dem. Rep.	39,861(3,061-75,864)	0.814(0.062,1.55)	287(19.49773028,546)
**Sub-Saharan Africa**	Congo, Rep.	2,641(181-5,007)	0.758(0.054,1.437)	332(24.3929967,629)
**Sub-Saharan Africa**	Cote d'Ivoire	55,203(3,897-107,216)	0.622(0.042,1.208)	1,447(96.68767702,2,810)
**Sub-Saharan Africa**	Equatorial Guinea*	3,557(242-6,898)	1.129(0.078,2.189)	1,686(112.0520488,3,269)
**Sub-Saharan Africa**	Eritrea*	3,727(259-7,236)	1.073(0.075,2.083)	792(59.57992423,1,538)
**Sub-Saharan Africa**	Eswatini	2,345(165-4,390)	0.793(0.057,1.484)	1,660(120.3663162,3,108)
**Sub-Saharan Africa**	Ethiopia	75,383(5,314-140,096)	0.352(0.025,0.654)	471(35.92891153,875)
**Sub-Saharan Africa**	Gabon	7,437(533-14,028)	0.724(0.049,1.365)	2,472(184,4,662)
**Sub-Saharan Africa**	Gambia, The	1,410(100-2,703)	0.563(0.043,1.079)	391(28.86018962,750)
**Sub-Saharan Africa**	Ghana	67,650(5,358-126,709)	0.817(0.064,1.529)	1,634(125.6993303,3,060)
**Sub-Saharan Africa**	Guinea	13,397(966-25,507)	0.596(0.042,1.135)	694(54.55002485,1,322)
**Sub-Saharan Africa**	Guinea-Bissau	963(77-1,785)	0.582(0.041,1.079)	352(24.268936,652)
**Sub-Saharan Africa**	Kenya	34,820(2,850-63,678)	0.314(0.026,0.575)	478(36.71098666,874)
**Sub-Saharan Africa**	Lesotho	1,097(89-1,978)	0.925(0.071,1.67)	455(38.52968745,820)
**Sub-Saharan Africa**	Liberia*	2,088(149-4,060)	0.987(0.074,1.92)	293(19.2090398,570)
**Sub-Saharan Africa**	Madagascar	8,025(605-15,178)	0.514(0.035,0.973)	199(14.39199722,377)
**Sub-Saharan Africa**	Malawi*	11,321(749-22,028)	0.954(0.064,1.857)	401(28.15416616,780)
**Sub-Saharan Africa**	Mali	11,633(787-22,596)	0.56(0.043,1.088)	373(24.68980133,724)
**Sub-Saharan Africa**	Mauritania	5,276(359-10,330)	0.497(0.036,0.973)	783(58.86598533,1,533)
**Sub-Saharan Africa**	Mauritius	20,617(1,551-38,026)	2.485(0.177,4.583)	16,483(1,256,30,401)
**Sub-Saharan Africa**	Mozambique	9,296(671-16,708)	0.509(0.039,0.914)	196(15.92223275,353)
**Sub-Saharan Africa**	Namibia	3,298(218-6,240)	0.572(0.042,1.082)	1,013(75.70299587,1,916)

**Sub-Saharan Africa**	Niger	9,542(689-18,530)	0.536(0.037,1.041)	224(16.33486824,434)
**Sub-Saharan Africa**	Nigeria	171,458(11,548-343,159)	0.569(0.039,1.139)	575(40.45898985,1,151)
**Sub-Saharan Africa**	Rwanda	9,544(776-17,490)	0.528(0.043,0.968)	532(38.35147237,974)
**Sub-Saharan Africa**	Sao Tome and Principe*	307(23-597)	0.931(0.064,1.813)	1,021(71.71661036,1,987)
**Sub-Saharan Africa**	Senegal	25,074(1,904-47,928)	0.771(0.052,1.474)	1,023(68.63163068,1,956)
**Sub-Saharan Africa**	Seychelles*	2,185(152-4,202)	2.19(0.144,4.212)	21,203(1,360,40,770)
**Sub-Saharan Africa**	Sierra Leone	1,080(78-2,057)	0.294(0.019,0.559)	103(6.662123554,196)
**Sub-Saharan Africa**	Somalia*	7,083(497-13,770)	0.997(0.069,1.938)	287(19.65982196,558)
**Sub-Saharan Africa**	South Africa	181,135(13,943-344,258)	0.92(0.065,1.749)	2,651(196,5,038)
**Sub-Saharan Africa**	South Sudan*	6,952(501-13,521)	0.971(0.073,1.888)	451(30.93244112,878)
**Sub-Saharan Africa**	Sudan	73,089(5,250-145,155)	1.39(0.086,2.761)	1,182(85.42352035,2,347)
**Sub-Saharan Africa**	Tanzania	49,047(4,168-88,900)	0.533(0.046,0.967)	534(41.40448571,967)
**Sub-Saharan Africa**	Togo	5,708(403-10,791)	0.629(0.049,1.189)	488(36.38952219,923)
**Sub-Saharan Africa**	Uganda	30,832(2,533-57,119)	0.586(0.047,1.085)	459(33.96357567,851)
**Sub-Saharan Africa**	Zambia	11,099(857-20,243)	0.506(0.040,0.923)	395(29.35247042,721)
**Sub-Saharan Africa**	Zimbabwe	8,712(590-16,445)	0.592(0.041,1.118)	452(35.31257294,853)
**Others**	Cook Islands*	449(34-859)	4.179(0.315,8.001)	25,752(1,979,49,300)
**Others**	Niue*	18(1-34)	4.973(0.326,9.513)	10,522(806,20,128)
**Others**	Palestine*	14,993(1,003-28,923)	1.617(0.120,3.119)	2,152(143,4,151)
**Others**	Tokelau*	8(1-16)	3.055(0.227,5.859)	5,723(370,10,977)

We also provided the aggregated results for World Bank regions and income groups. **Table S9** and **Table S10** show the results with varying discount rates of 0% and 3% without informal care. **Table S11** shows the results with varying weekly informal care hours with 4.0 as the baseline (lower bound of 0.283 and upper bound of 8.3), using a discount rate of 2%.

Table S9. Total macroeconomic cost, economic cost as a percentage of total GDP in 2020–2050, and per capita economic cost attributable to diabetes mellitus mortality and morbidity by World Bank region and by World Bank income group in 2017 international dollars (INT$), with discount rate of 0%, without informal care, and with baseline (lower bound-upper bound) mortality and morbidity.

Region/income group	Economic cost in billions of 2017 INT$	Percentage of total GDP in 2020-2050	Per capita loss in 2017 INT$
**By World Bank region**			
**East Asia & Pacific**	4,742(3,560-6,299)	0.205(0.154-0.273)	1,927(1,447-2,560)
**Europe & Central Asia**	3,070(2,356-4,009)	0.231(0.178-0.302)	3,299(2,532-4,308)
**Latin America & Caribbean**	947(702-1,264)	0.252(0.187-0.336)	1,319(978-1,760)
**Middle East & North Africa**	745(556-1,002)	0.210(0.156-0.282)	1,326(989-1,784)
**North America**	3,884(3,319-4,564)	0.417(0.357-0.491)	9,724(8,311-11,428)
**South Asia**	1,908(1,314-2,686)	0.210(0.144-0.295)	904(623-1,273)
**Sub-Saharan Africa**	247(165-363)	0.103(0.069-0.152)	150(101-221)

Region/income group	Economic cost in billions of 2017 INT$	Percentage of total GDP in 2020-2050	Per capita loss in 2017 INT$
By World Bank income group			
Low income	100(65-150)	0.107(0.070-0.161)	104(67-155)
Lower middle income	3,582(2,444-5,081)	0.221(0.151-0.314)	910(621-1,290)
Upper middle income	4,057(3,103-5,307)	0.180(0.137-0.235)	1,531(1,171-2,003)
High income	7,762(6,335-9,587)	0.315(0.257-0.389)	6,266(5,114-7,739)
Total	15,546(11,975-20,191)	0.241(0.186-0.313)	1,761(1,356-2,287)

Table S10. Total macroeconomic cost, economic cost as a percentage of total GDP in 2020–2050, and per capita economic cost attributable to diabetes mellitus mortality and morbidity by World Bank region and by World Bank income group in 2017 international dollars (INT$), with discount rate of 3%, without informal care, and with baseline (lower bound-upper bound) mortality and morbidity.

Region/income group	Economic cost in billions of 2017 INT$	Percentage of total GDP in 2020-2050	Per capita loss in 2017 INT$
By World Bank region			
East Asia & Pacific	2,524(1,901-3,342)	0.182(0.137-0.240)	1,026(773-1,358)
Europe & Central Asia	1,644(1,263-2,145)	0.201(0.154-0.262)	1,767(1,357-2,305)
Latin America & Caribbean	515(384-685)	0.217(0.162-0.289)	718(534-955)
Middle East & North Africa	395(296-530)	0.183(0.137-0.246)	704(526-944)
North America	2,133(1,822-2,507)	0.369(0.315-0.433)	5,342(4,563-6,278)
South Asia	976(675-1,370)	0.189(0.131-0.266)	463(320-649)
Sub-Saharan Africa	133(90-195)	0.094(0.063-0.137)	81(55-119)
By World Bank income group			
Low income	53(34-79)	0.098(0.064-0.147)	55(36-82)
Lower middle income	1,846(1,265-2,610)	0.198(0.136-0.280)	469(321-663)
Upper middle income	2,180(1,671-2,844)	0.159(0.122-0.207)	823(631-1,073)
High income	4,221(3,446-5,209)	0.276(0.225-0.340)	3,407(2,781-4,204)
Total	8,324(6,431-10,777)	0.213(0.165-0.276)	943(728-1,221)

Table S11. Total macroeconomic cost, economic cost as a percentage of total GDP in 2020–2050, and per capita economic cost attributable to diabetes mellitus mortality, morbidity, and informal care, by World Bank region and by World Bank income group in 2017 international dollars (INT$), with discount rate of 2%, with baseline mortality and morbidity, and with 4.0 baseline (0.283 lower bound-8.3 upper bound) weekly informal care hours.

Region/income group	Economic cost in billions of 2017 INT$	Percentage of total GDP in 2020-2050	Per capita loss in 2017 INT$
By World Bank region			
East Asia & Pacific	20,793(1,490-39,756)	1.271(0.091-2.430)	8,449(606-16,154)
Europe & Central Asia	18,181(1,256-35,464)	1.902(0.132-3.709)	19,538(1,351-38,109)
Latin America & Caribbean	4,147(298-7,903)	1.508(0.108-2.874)	5,775(415-11,006)
Middle East & North Africa	4,035(282-7,817)	1.595(0.111-3.089)	7,183(502-13,915)
North America	17,330(1,244-33,093)	2.573(0.184-4.913)	43,392(3,114-82,860)
South Asia	13,232(897-26,088)	2.136(0.145-4.211)	6,269(425-12,360)
Sub-Saharan Africa	1,068(77-2,039)	0.636(0.046-1.213)	650(47-1,242)

**By World Bank income group**			
**Low income**	436(31-834)	0.680(0.049-1.301)	453(33-867)
**Lower middle income**	19,943(1,384-38,824)	1.789(0.124-3.484)	5,064(352-9,859)
**Upper middle income**	19,286(1,369-37,087)	1.197(0.085-2.302)	7,278(517-13,995)
**High income**	38,918(2,747-75,023)	2.184(0.154-4.211)	31,414(2,220-60,557)
**Total**	78,801(5,549-152,189)	1.720(0.121-3.322)	8,924(628-17,235)

E: Contribution of treatment costs and informal labor costs

Figure S7 shows the contribution of informal labor costs to the total macroeconomic burden of diabetes mellitus by country income group for the baseline scenario.

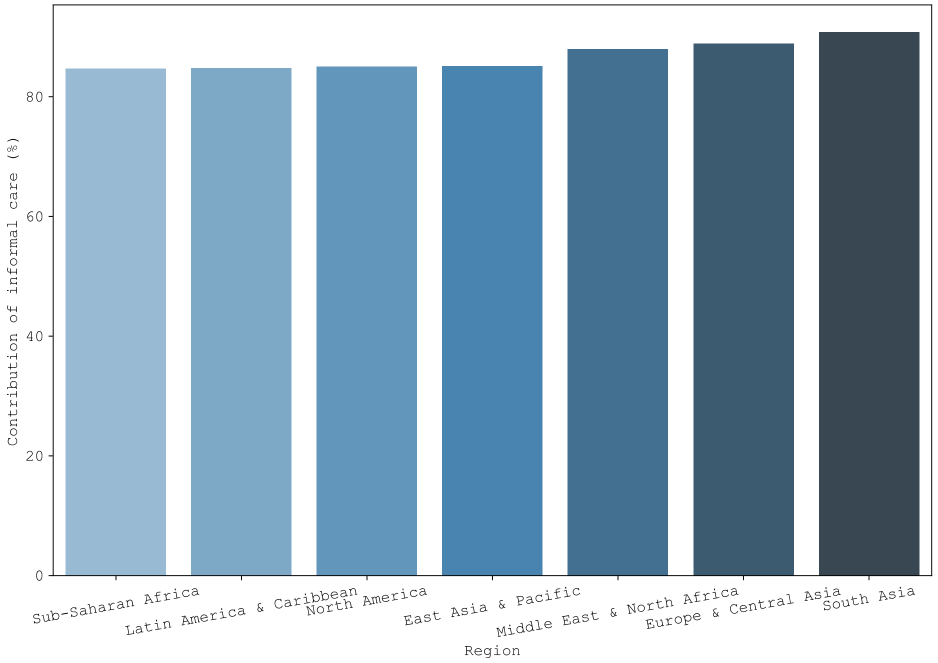

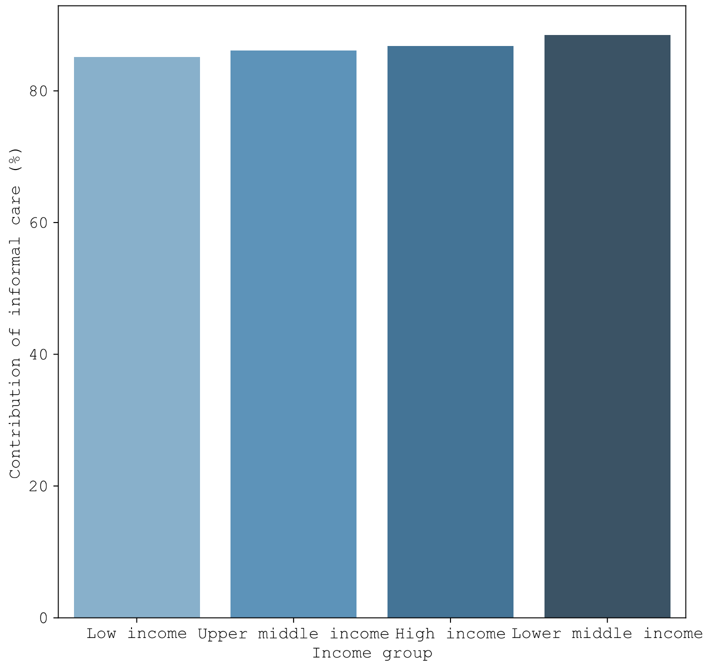

Figure S7. Contribution of informal labor costs to the total economic loss from diabetes mellitus by World Bank region (top) and income group (bottom)

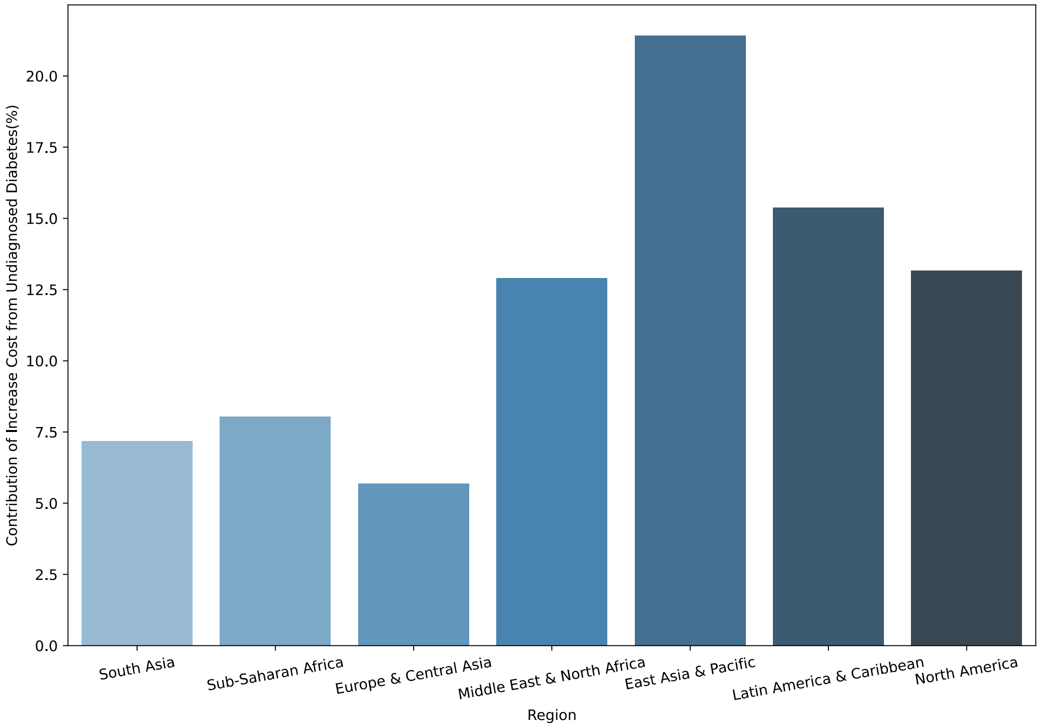

Figure S8. Contribution of increase cost from undiagnosed diabetes

F: Strengths and limitations

Strengths

- Our framework is the first to consider economic adjustment mechanisms (substitution of labor lost because of diabetes mellitus and savings responses to diabetes mellitus healthcare costs) explicitly in analyzing the economic burden of diabetes mellitus; the lack of such consideration is a key limitation of previous studies on diabetes mellitus.
- This study is the first to account simultaneously for the influence of diabetes mellitus on economic growth through informal care, mortality, morbidity, and the effect of treatment expenditures for 204 countries worldwide.
- This study is the first to incorporate age-specific human capital to account for education-related productivity differences among members of different cohorts affected by diabetes mellitus in 204 countries worldwide.
- Our work shows the causal impact of diabetes mellitus on GDP; it avoids issues of reverse causality because we do not estimate the relationship but construct it from our simulated production function.
- We provide a detailed, step-by-step description of our methods and the data sources and specific parameters used in our analysis in the SI Appendix.
- We performed sensitivity analyses to account for underlying uncertainty by adjusting the mortality and morbidity data based on the upper and lower bounds of the GBD data. In addition, we performed sensitivity analyses concerning the discount rate and the informal care load.
- We calculated and compared the lifetime health burden of diabetes mellitus with its economic burden to show global inequalities.
- We calculated the economic burden of new cases of and deaths from diabetes mellitus due to COVID-19.

Limitations

- We used results on diabetes mellitus–related health expenditures from Dieleman et al. (2020), which may either underestimate or overestimate country-specific treatment costs of diabetes mellitus. For reliability, we compared these data with projections from the International Diabetes Federation. The treatment cost values from the International Diabetes Federation are about 1.14-9.96 times our projections. Therefore, our treatment costs could be underestimations.
- Due to missing data, we had to impute the economic burden of diabetes mellitus for 60 out of 204 countries. However, this does not significantly compromise our results, given that the 144 countries for which we had complete data which account for more than 92.7% of global population. Different countries report health expenditure, demographic, and economic data using varying methodologies and timelines. For example, some countries provided recent, detailed data, while others relied on global database estimates, which may influence the precision of individual country results. To address these uncertainties, we used sensitivity analyses (Table S4) to evaluate the impact of imputations and variability in data sources on global estimates. These analyses showed deviations of less than 30%, confirming the robustness of the global results. Further details on missing data are provided in Table S3, and the imputation process is described in Table S4.
- We did not include the burden of undiagnosed diabetes mellitus, which accounts for about 44.7% of total diabetes mellitus cases.[19] Undiagnosed patients may not need treatment because they are unaware of their disease, but their productivity might be lower than that of others due to morbidity. Thus, we underestimated the labor loss due to undiagnosed illness.

Table S12. Strengths and limitations

Strengths	Limitations
**First framework to incorporate economic adjustment mechanisms (labor substitution and savings responses) in estimating the economic burden of diabetes.**	Treatment cost estimates may be underestimated, as values from Dieleman et al. (2020) are lower than those from the IDF.
**Simultaneous consideration of mortality, morbidity, treatment costs, and informal care across 204 countries.**	Imputation required for 60 countries (7.3% of global population); however, sensitivity analyses showed robustness (<30% deviation).
**Incorporation of age-specific human capital to capture**	Cross-country variation in reporting methods and

**education-related productivity differences.**	timelines may affect precision.
**Causal inference through simulated production function, avoiding reverse causality.**	Exclusion of undiagnosed diabetes (~44.7% of cases), potentially underestimating labor losses.
**Transparent methodological description and extensive sensitivity analyses (mortality, morbidity, discount rate, informal care).**	—
**Comparison of lifetime health vs. economic burden, and estimation of COVID-19–related diabetes burden.**	—

References

1. GBD 2019 Diseases and Injuries Collaborators. Global burden of 369 diseases and injuries in 204 countries and territories, 1990-2019: a systematic analysis for the Global Burden of Disease Study 2019. *Lancet* 2020; **396**(10258): 1204-22.
2. Brauer M, Roth GA, Aravkin AY, et al. Global burden and strength of evidence for 88 risk factors in 204 countries and 811 subnational locations, 1990–2021: a systematic analysis for the Global Burden of Disease Study 2021. *The Lancet* 2024; **403**(10440): 2162-203.
3. Barro RJ, Lee JW. A new data set of educational attainment in the world, 1950–2010. *Journal of Development Economics* 2013; **104**(September 2013): 184–98.
4. Vos T, Lim SS, Abbafati C, et al. Global burden of 369 diseases and injuries in 204 countries and territories, 1990-2019: a systematic analysis for the Global Burden of Disease Study 2019. *The Lancet* 2020; **396**(10258): 1204-22.
5. World Bank. World Bank database, GDP (constant 2017 international $). 2022. https://data.worldbank.org/indicator/NY.GDP.MKTP.PP.KD?view=chart. (accessed Aug 7 2022).
6. International Monetary Fund. World Economic Outlook Database. 2020. https://www.imf.org/external/pubs/ft/weo/2017/01/weodata/download.aspx (accessed Sept 1 2020).
7. Agency CI. The World Factbook-Real GDP (purchasing power parity). 2022. https://www.cia.gov/the-world-factbook/field/real-gdp-purchasing-power-parity/country-comparison (accessed 28 April 2022).
8. University of Groningen and University of California. Share of Labour Compensation in GDP at Current National Prices for United States [LABSHPUSA156NRUG], retrieved from FRED, Federal Reserve Bank of St. Louis Davis. 2021. https://fred.stlouisfed.org/series/LABSHPUSA156NRUG (accessed Jan 10 2021).
9. International Labour Organization. Labour force by sex and age (thousands). 2020. http://ilo.org/global/statistics-and-databases/lang--en/index.htm (accessed Sept 1 2020).
10. United Nations. World Population Prospects 2019. 28 August 2019 2019. https://population.un.org/wpp/Download/Standard/CSV/2022).
11. World Bank. World Bank database, gross savings (% of GDP). 2022. https://data.worldbank.org/indicator/NY.GNS.ICTR.ZS (accessed Aug 7 2022).
12. Langa KM, Vijan S, Hayward RA, et al. Informal caregiving for diabetes and diabetic complications among elderly americans. *J Gerontol B Psychol Sci Soc Sci* 2002; **57**(3): S177-86.
13. International Labour Organization. Average hours and prevalence of excessive working time. 2022. https://ilostat.ilo.org/topics/working-time/# (accessed April 29 2022).
14. Dieleman JL, Cao J, Chapin A, et al. US Health Care Spending by Payer and Health Condition, 1996-2016. *JAMA* 2020; **323**(9): 863-84.
15. Bloom DE, Cafiero E, Jané-Llopis E, et al. The global economic burden of noncommunicable diseases. Geneva, Switzerland: World Economic Forum, 2011.
16. Ding D, Lawson KD, Kolbe-Alexander TL, et al. The economic burden of physical inactivity: a global analysis of major non-communicable diseases. *The Lancet* 2016; **388**(10051): 1311-24.
17. Chen S, Kuhn M, Prettner K, Bloom DE. The global macroeconomic burden of road injuries: estimates and projections for 166 countries. *The Lancet Planetary Health* 2019; **3**(9): e390-e8.
18. Williams R, Karuranga S, Malanda B, et al. Global and regional estimates and projections of diabetes-related health expenditure: Results from the International Diabetes Federation Diabetes Atlas, 9th edition. *Diabetes Res Clin Pract* 2020; **162**: 108072.
19. International Diabetes Federation. IDF Diabetes Atlas, 10th edition. 2021. 2021. http://www.diabetesatlas.org (accessed July 27 2022).
20. Bloom DE, Chen S, Kuhn M, McGovern ME, Oxley L, Prettner K. The economic burden of chronic diseases: Estimates and projections for China, Japan, and South Korea. *The Journal of the Economics of Ageing* 2020; **17**(2020): 10016.
21. Chen S, Kuhn M, Prettner K, Bloom DE. Noncommunicable Diseases Attributable To Tobacco Use In China: Macroeconomic Burden And Tobacco Control Policies. *Health Affairs* 2019; **38**(11): 1832-9.
22. Grossmann V, Steger T, Trimborn T. Dynamically optimal R&D subsidization. *Journal of Economic Dynamics and Control* 2013; **37**(3): 516-34.
23. Psacharopoulos G, Patrinos HA. Returns to investment in education: a decennial review of the global literature. *Education Economics* 2018; **26**(5): 445-58.
24. Heckman JJ, Lochner LJ, Todd PE. Earnings functions, rates of return and treatment effects: The Mincer equation and beyond. *Handbook of the Economics of Education* 2006; **1**(2006): 307–458.
