# Supplementary material for: The global macroeconomic burden of diabetes mellitus
Source: Nat Med. 2025 Dec 29;32(1):126–38. doi: 10.1038/s41591-025-04027-5 (PMC12823416; doi:10.1038/s41591-025-04027-5)
Supplement: Supplementary file 2 — Reporting Summary [file 41591_2025_4027_MOESM2_ESM.pdf]

nature portfolio

Corresponding author(s): Simiao Chen

Last updated by author(s): Sep 19, 2025

Reporting Summary

Nature Portfolio wishes to improve the reproducibility of the work that we publish. This form provides structure for consistency and transparency in reporting. For further information on Nature Portfolio policies, see our Editorial Policies and the Editorial Policy Checklist.

Statistics

For all statistical analyses, confirm that the following items are present in the figure legend, table legend, main text, or Methods section.

n/a	Confirmed	
☒	☐	The exact sample size (n) for each experimental group/condition, given as a discrete number and unit of measurement
☒	☐	A statement on whether measurements were taken from distinct samples or whether the same sample was measured repeatedly
☒	☐	The statistical test(s) used AND whether they are one- or two-sided<br>*Only common tests should be described solely by name; describe more complex techniques in the Methods section.*
☒	☐	A description of all covariates tested
☐	☒	A description of any assumptions or corrections, such as tests of normality and adjustment for multiple comparisons
☐	☒	A full description of the statistical parameters including central tendency (e.g. means) or other basic estimates (e.g. regression coefficient) AND variation (e.g. standard deviation) or associated estimates of uncertainty (e.g. confidence intervals)
☒	☐	For null hypothesis testing, the test statistic (e.g. F, t, r) with confidence intervals, effect sizes, degrees of freedom and P value noted<br>*Give P values as exact values whenever suitable.*
☒	☐	For Bayesian analysis, information on the choice of priors and Markov chain Monte Carlo settings
☐	☒	For hierarchical and complex designs, identification of the appropriate level for tests and full reporting of outcomes
☐	☒	Estimates of effect sizes (e.g. Cohen's d, Pearson's r), indicating how they were calculated

Our web collection on statistics for biologists contains articles on many of the points above.

Software and code

Policy information about availability of computer code

Data collection	The analysis of this study used publicly available summary data from international knowledge bases and previously published sources.
Data analysis	All code for the descriptive tables and the primary and safety end point analyses is publicly available at https://github.com/caozhong14/HMM diabetes.git

For manuscripts utilizing custom algorithms or software that are central to the research but not yet described in published literature, software must be made available to editors and reviewers. We strongly encourage code deposition in a community repository (e.g. GitHub). See the Nature Portfolio guidelines for submitting code & software for further information.

Data

Policy information about availability of data

All manuscripts must include a data availability statement. This statement should provide the following information, where applicable:

- Accession codes, unique identifiers, or web links for publicly available datasets
- A description of any restrictions on data availability
- For clinical datasets or third party data, please ensure that the statement adheres to our policy

All data used in this study are publicly available from existing repositories and databases. Detailed descriptions of the data sources, access links, and processing procedures are provided in the Methods section and the. No new datasets were generated for this study. Source data underlying the figures are provided with the paper.

Research involving human participants, their data, or biological material

Policy information about studies with human participants or human data. See also policy information about sex, gender (identity/presentation), and sexual orientation and race, ethnicity and racism.

Reporting on sex and gender	The study uses publicly available,aggregated"age-sex-specific" data for labor force projections,education,and population. Sex is treated as a biological variable in the analysis. No individual-level data was collected.
Reporting on race, ethnicity, or other socially relevant groupings	The study does not use race or ethnicity for categorization. The primary groupings are geographical and economic.
Population characteristics	This study is a macroeconomic analysis using aggregated public data from 204 countries;it did not recruit human participants. Population characteristics are defined at an aggregate level,including data on demographics, mortality, morbidity, education, and labor force participation.
Recruitment	NO participants were recruited for this study.
Ethics oversight	Ethics approval was not required. No individual-level human data were collected.

Note that full information on the approval of the study protocol must also be provided in the manuscript.

Field-specific reporting

Please select the one below that is the best fit for your research. If you are not sure, read the appropriate sections before making your selection.

☐ Life sciences ☒ Behavioural & social sciences ☐ Ecological, evolutionary & environmental sciences

For a reference copy of the document with all sections, see nature.com/documents/nr-reporting-summary-flat.pdf

Behavioural & social sciences study design

All studies must disclose on these points even when the disclosure is negative.

Study description	This is a quantitative, macroeconomic modeling study.
Research sample	The study uses aggregated, publicly available data for 204 countries and territories, Datasets, were sourced form the Global Burden of Disease GBD)2021 Study, the World Bank, and the International Labor Organization,among others.
Sampling strategy	The study did not use sampling.
Data collection	No new data was collected for this study.
Timing	The study projects the economic burden from 2020 to 2050.The authors accessed the various existing datasets used for the analysis between June 2017 and August 2022.
Data exclusions	No data were excluded from the analyses.
Non-participation	Not applicable.
Randomization	Participants were not allocated to experimental groups.

Reporting for specific materials, systems and methods

We require information from authors about some types of materials, experimental systems and methods used in many studies. Here, indicate whether each material, system or method listed is relevant to your study. If you are not sure if a list item applies to your research, read the appropriate section before selecting a response.

Materials & experimental systems

n/a	Involved in the study
☒	☐ Antibodies
☒	☐ Eukaryotic cell lines
☒	☐ Palaeontology and archaeology
☒	☐ Animals and other organisms
☐	☒ Clinical data
☒	☐ Dual use research of concern
☒	☐ Plants

Methods

n/a	Involved in the study
☒	☐ ChIP-seq
☒	☐ Flow cytometry
☒	☐ MRI-based neuroimaging

Clinical data

Policy information about clinical studies

All manuscripts should comply with the ICMJE guidelines for publication of clinical research and a completed CONSORT checklist must be included with all submissions.

Clinical trial registration	Not applicable.
Study protocol	Not applicable.
Data collection	This study did not involve primary data collection.
Outcomes	The primary outcome was the macroeconomic burden of diabetes mellitus,calculated as the cumulative difference in projected GDP between"status quo"scenario and a"counter factual" scenario over the period2020-2050.

Plants

Seed stocks	Not applicable.
Novel plant genotypes	Not applicable.
Authentication	Not applicable.
